# Multipotent Basal Stem Cells, Maintained in Localized Proximal Niches, Support Directed Long-Ranging Epithelial Flows in Human Prostates

**DOI:** 10.1016/j.celrep.2017.07.061

**Published:** 2017-08-15

**Authors:** Mohammad Moad, Edouard Hannezo, Simon J. Buczacki, Laura Wilson, Amira El-Sherif, David Sims, Robert Pickard, Nicholas A. Wright, Stuart C. Williamson, Doug M. Turnbull, Robert W. Taylor, Laura Greaves, Craig N. Robson, Benjamin D. Simons, Rakesh Heer

**Affiliations:** 1 Northern Institute for Cancer Research, Newcastle University, Newcastle upon Tyne NE2 4AD, UK; 2 Cavendish Laboratory, Department of Physics, University of Cambridge, J.J. Thomson Avenue, Cambridge CB3 0HE, UK; 3 Wellcome Trust/Cancer Research UK Gurdon Institute, University of Cambridge, Tennis Court Road, Cambridge CB2 1QN, UK; 4 Cancer Research UK, Cambridge Institute, University of Cambridge, Cambridge CB2 0RE, UK; 5 Department of Histopathology, Royal Victoria Infirmary, Newcastle upon Tyne NE1 4LP, UK; 6 Department of Pathology, Faculty of Medicine, Menoufia University, Menoufia, Egypt; 7 Computational Genomics Analysis and Training (CGAT), MRC Functional Genomics Unit, Department of Physiology, Anatomy and Genetics, University of Oxford, Oxford OX1 3PT, UK; 8 Institute of Cellular Medicine, Medical School, Newcastle University, Newcastle upon Tyne NE2 4HH, UK; 9 Barts Cancer Institute, Barts and the London School of Medicine and Dentistry, Queen Mary University of London, London EC1M 6BQ, UK; 10 Clinical and Experimental Pharmacology Group, University of Manchester, Manchester M13 9PL, UK; 11 Wellcome Trust Centre for Mitochondrial Research, Institute of Neuroscience, Newcastle University, Newcastle upon Tyne NE2 4HH, UK; 12 Newcastle Centre for Ageing and Vitality, Newcastle University, Newcastle upon Tyne NE2 4HH, UK; 13 Wellcome Trust/Medical Research Council Stem Cell Institute, Cambridge CB2 1QR, UK

**Keywords:** stem cell, niche, prostate, epithelium, basal, luminal, branch, DLK1, Notch, organoids, prostate cancer

## Abstract

Sporadic mitochondrial DNA mutations serve as clonal marks providing access to the identity and lineage potential of stem cells within human tissues. By combining quantitative clonal mapping with 3D reconstruction of adult human prostates, we show that multipotent basal stem cells, confined to discrete niches in juxta-urethral ducts, generate bipotent basal progenitors in directed epithelial migration streams. Basal progenitors are then dispersed throughout the entire glandular network, dividing and differentiating to replenish the loss of apoptotic luminal cells. Rare lineage-restricted luminal stem cells, and their progeny, are confined to proximal ducts and provide only minor contribution to epithelial homeostasis. In situ cell capture from clonal maps identified delta homolog 1 (DLK1) enrichment of basal stem cells, which was validated in functional spheroid assays. This study establishes significant insights into niche organization and function of prostate stem and progenitor cells, with implications for disease.

## Highlights

• Discrete, proximal stem cell niche domains in juxta-urethral ducts
• Basal stem cells generate large-scale, directed, and cohesive cell migration streams
• Bipotent basal progenitors maintain the luminal compartment
• DLK1 marks stem cells in situ that recapitulate gland histology in 3D culture

## Graphical Abstract

**Figure:**
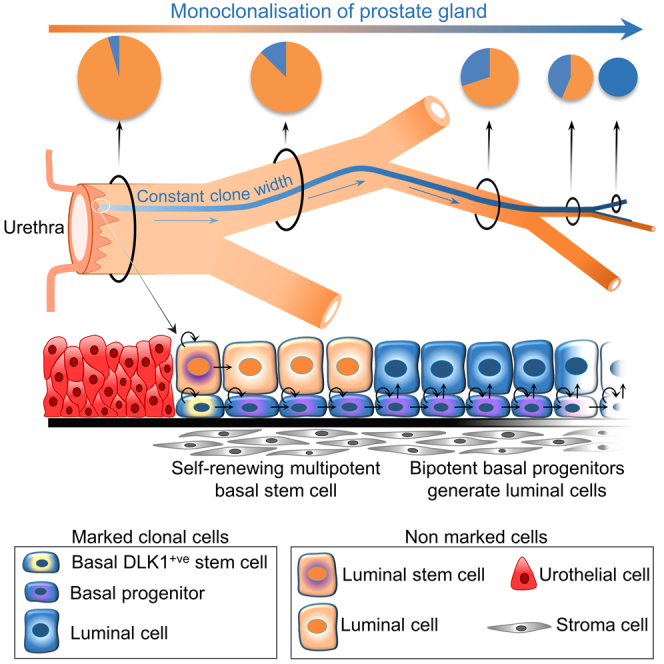

## Teaser

Moad et al. find that multipotent prostate basal stem cells, marked by delta homolog 1 (DLK1), reside in proximal ducts and generate directed large-scale epithelial flows traversing the entire length of the branching gland network. This work describes mechanisms underlying 3D epithelial homeostasis in a complex branching tissue.

## Introduction

**Figure 1:**
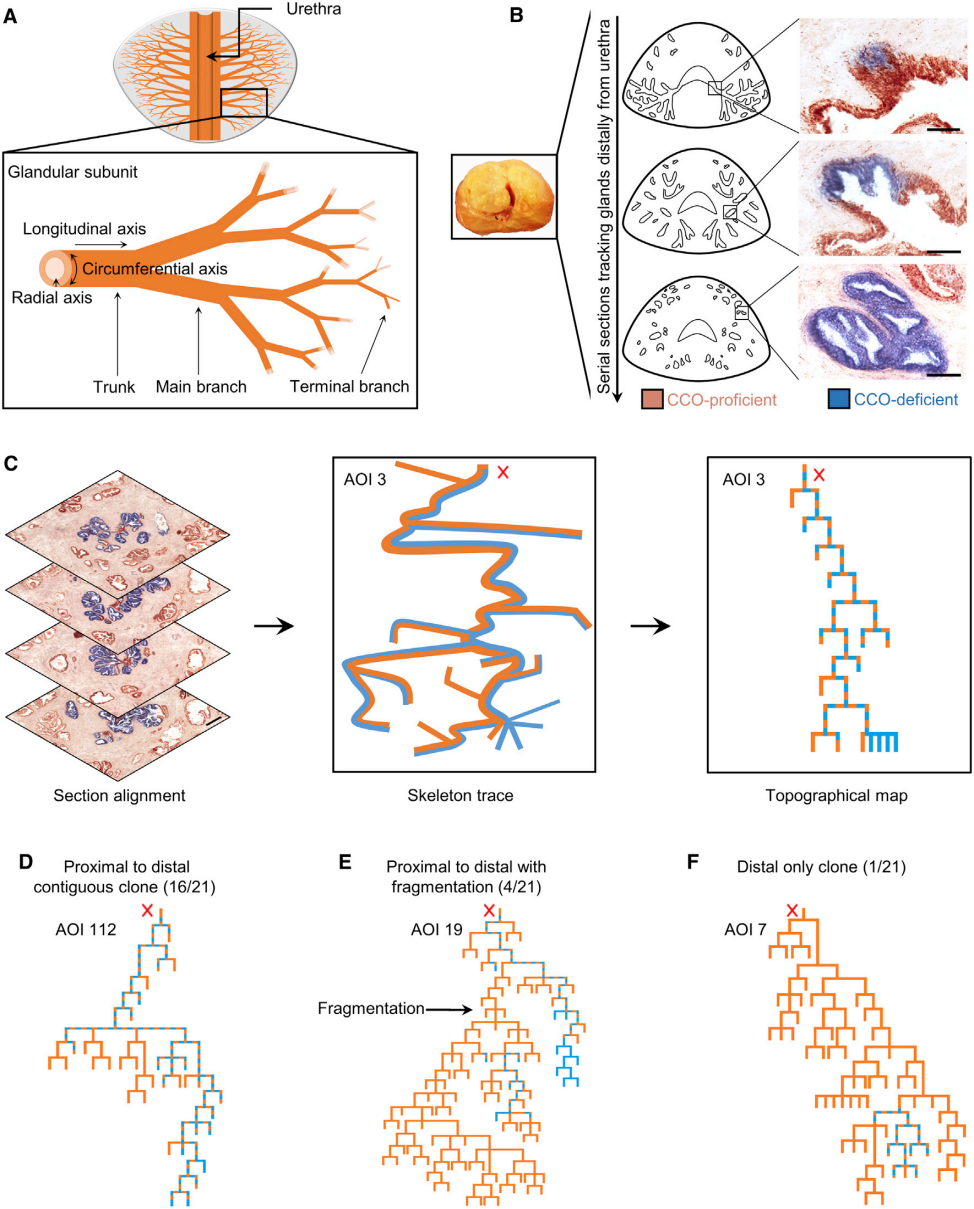
Transmission of mtDNA Identifies Long-Ranging Clones Spanning the Entire Prostate from Proximal Juxta-Urethra Ducts to Distal Acini (A) The prostate comprises 12–18 paired glandular subunits independently draining into the urethra. (B) Two-color enzyme histochemistry simultaneously detects activity of the mtDNA-encoded CCO and nuclear-DNA-encoded succinate dehydrogenase (SDH), with CCO-deficient cells appearing blue and CCO-proficient cells appearing brown. Scale bars, 50 μm. (C) Serial sections are aligned in reconstruction software to generate a 3D wire-frame reconstruction of glandular subunit. The reconstruction is converted to a topographical representation to more clearly illustrate spread of the CCO-deficient patch through ductal epithelium (solid brown line represents homogeneous CCO proficiency; blue-brown dashed line, mosaic CCO-deficiency; solid blue line, homogeneous CCO-deficiency). “X” marks common duct opening onto the urethra. Scale bar, 200 μm. (D–F) Three types of clone pattern distributions were identified: long-ranging clones (95%) composed of proximal to distal contiguous patches (75%) (left), occasional proximal to distal clones with fragmentation (20%) (center), and a single rare example of a distal-only clone (5%) (right).

The prostate consists of distinct glandular subunits that independently drain proximally into the prostatic urethra (McNeal, 1968) (Figure 1A). The glandular subunits comprise a complex branching ductal network of stratified epithelia composed of basal, luminal, and sparse neuroendocrine cells (Shen and Abate-Shen, 2010). In situ genetic labeling studies in mouse suggest that the adult prostate is largely maintained by the slow turnover of distinct lineage-restricted cytokeratin 5 (CK5)-expressing basal and CK8/CK18-expressing luminal cells (Choi et al., 2012, Ousset et al., 2012), while a minority of basal cells show evidence of bipotency (Lu et al., 2013, Wang et al., 2013). Lineage tracing studies of tissue regeneration, following cyclical androgen deprivation, also point to rare bipotent castration-resistant luminal stem cells (Wang et al., 2009, Wang et al., 2013, Chua et al., 2014). However, differences in both lifespan and histological organization in mice question the applicability of these findings to the human prostate, where the identity of stem cells is unknown (Huang and Witte, 2010).

In humans, bipotent basal progenitors have been primarily characterized by ex vivo selection of putative stem cell-enriching markers and a combination of in vitro culture and in vivo xenograft regeneration assays (Cunha, 1972, Xin et al., 2003, Richardson et al., 2004, Goldstein et al., 2008, Leong et al., 2008). However, rare bipotent human luminal cells are described in vitro, but not in xenograft assays (Goldstein et al., 2008, Karthaus et al., 2014). Although ex vivo regeneration assays were developed as a model to study homeostatic biology within the human prostate, emerging evidence suggests that cell behaviors during regeneration may be markedly different to normal physiology (Donati and Watt, 2015, Tetteh et al., 2015). However, in contrast to animal studies, the in situ lineage potential of human prostate cells is currently poorly defined.

Previously, using mtDNA mutations as a surrogate lineage tracing mark, we showed in vivo evidence for local cohesive clonal patches containing both basal and luminal cells, along with neuroendocrine cells (Blackwood et al., 2011). Whether this reflects multipotency of luminal and/or basal lineages remains unknown, while key questions concerning the existence, identity, and function of the stem cell compartment within the human prostate remain subject to conjecture. Here, using a combination of quantitative lineage tracing studies, 3D glandular reconstructions, proliferation kinetics, and functional assays of differentiation, we define the location, lineage potential, and functional behavior of stem cells and their progeny in the adult human prostate.

## Results

### mtDNA Mutations Mark Long-Ranging Clones In Situ and Function as a Neutral Marker of Cells in the Human Prostate

To trace the cellular dynamics of in the human prostate, we employed a lineage labeling strategy based on the sporadic acquisition of mtDNA mutations (Taylor et al., 2003, Blackwood et al., 2011, Gaisa et al., 2011) using histocytochemical cytochrome *c* oxidase (CCO) deficiency as a reporter (Supplemental Experimental Procedures). 3D glandular reconstruction of the enzyme histochemistry using serial sections of entire human prostates characterized the topology of the epithelial branching network as well as the size and spatial organization of CCO-deficient clones (Figures 1B and 1C; Movie S1). Alongside small clonal patches (of 4–6 cell diameters), marking progenitor cell progeny that were seen to be dispersed sporadically and widely throughout the prostate (Blackwood et al., 2011, Gaisa et al., 2011), 3D glandular reconstructions revealed rare and large cohesive CCO-deficient patches, typically consisting of hundreds of thousands of cells and spanning entire individual glandular subunits (Figures 1D–1F).

To address the implications of such long-ranging clones, we first assessed whether mtDNA mutation serves as a neutral marker in the human prostate in light of previous studies raising concerns about a bias affecting cell fate through altered proliferation, differentiation, and apoptosis (Payne et al., 2005). Measuring both the proliferation and apoptosis rates of CCO-deficient and CCO-proficient epithelial cells, we found no statistically significant differences between them (Figures S1A and S1B). Moreover, CCO-deficient cells were present in both basal and luminal differentiated layers in a ratio statistically equivalent to that of the CCO-proficient epithelium (Figures S1C and S1D). Further evidence for the utility of CCO deficiency as a clonal tracer in prostate comes from the incidence of this mark within the gland. We found that the prostates examined were organized into 26 ± 2 (mean ± SD, n = 10 prostates) independent branching structures or subunits, as previously described (McNeal, 1968), which open separately into the urethra. The overwhelming majority (86% ± 4%) of prostate subunits did not contain extended CCO-deficient patches (Figures S1E and S1F), providing quantitative evidence that patches arise from discrete clonal events (Figure S1G; Supplemental Experimental Procedures). Moreover, older patients displayed a larger fraction of labeled subunits, in a manner quantitatively consistent with stochastic clonal induction occurring at a constant rate throughout the lifetime of the adult prostate.

### Stem Cells Localized at the Proximal Junction of Glandular Units Give Rise to Progeny that Migrate in Coherent Steams along the Proximal-Distal Axis

The spatial organization and extension of labeled patches of cells along the proximal-distal axis question their origin. In principle, such an arrangement could derive from the competition and turnover of equipotent stem cells distributed throughout the prostate, leading to bidirectional expansion of labeled clones along the ducts. Alternatively, such clonal structures might derive from the unidirectional flow of migratory cells from a localized stem cell niche domain, analogous to that characterized in the intestinal crypt (Winton and Ponder, 1990, Barker et al., 2007, Lopez-Garcia et al., 2010, Snippert et al., 2010, Baker et al., 2014).

**Figure 2:**
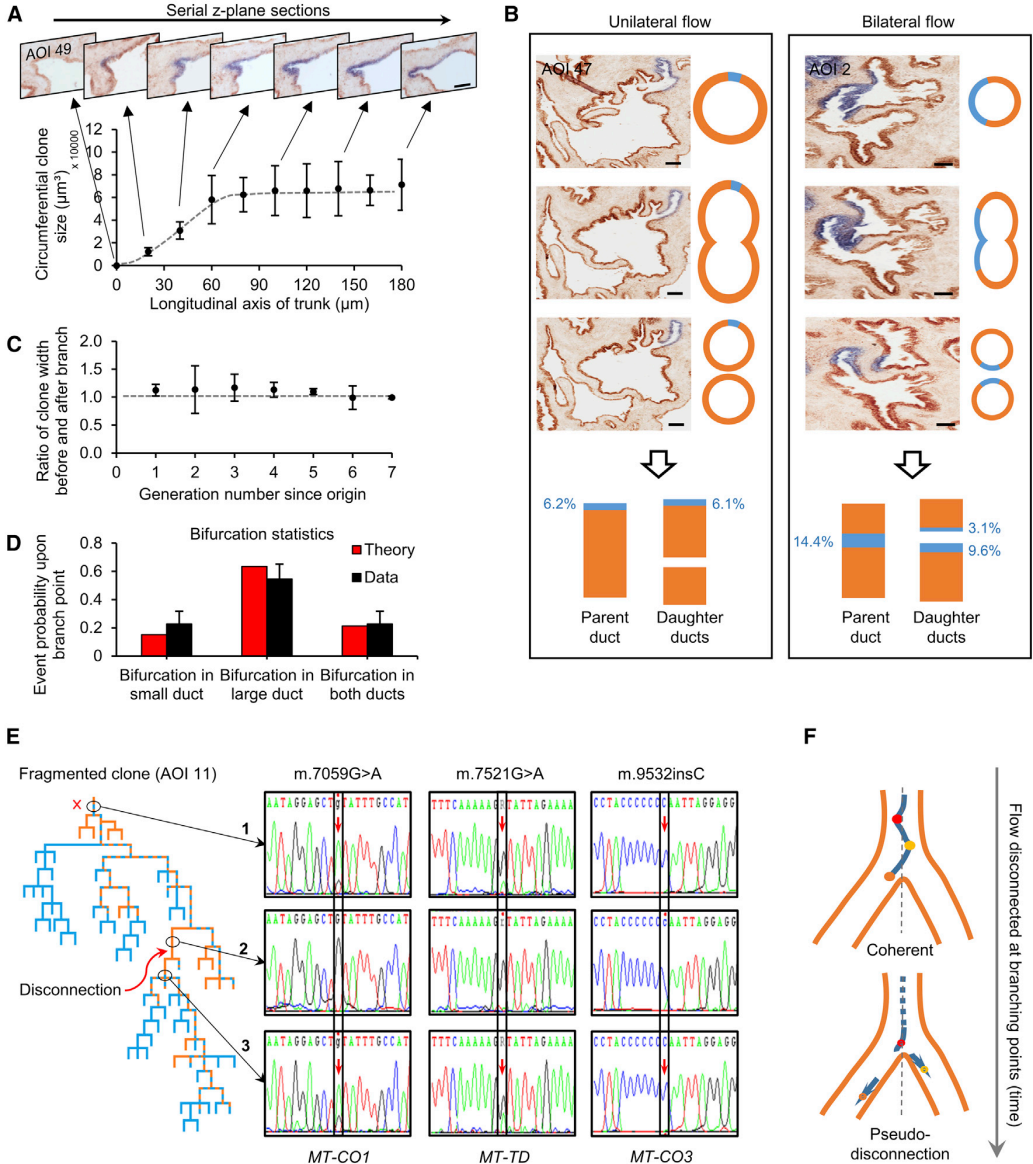
Directed Flow of Coherent Streams Reveals Proximal Juxta-Urethral Stem Cell Niche Domains (A) Constant circumferential widths of individual clonal patches are generated in the common trunks following a short region of transient expansion (n = 5 clones; n = 3 prostates; error bars represent SEM). A representative histology “filmstrip” is shown, capturing the start of a CCO-deficient patch in sequential z-plane images. Scale bar, 100 μm. (B) Two patterns of clone transmission are seen at duct branching, either unilateral or bilateral flow, with the clone width in parent duct always shared proportionately into the daughter ducts, consistent with directed flow. Scale bars, 100 μm. (C) Proportion of clone width in parent duct compared with the proportion in daughter ducts is maintenance throughout the branching tree. A ratio of cumulative clone width before and after branching is presented. Error bars, SD. (D) Passive and random flow of streams into daughter branches was theoretically modeled according to duct size and fraction of parent duct occupied by the duct. The predicted probabilities of outcome upon branching were consistent with observed data, showing that streams behave and branch neutrally. (E) Laser capture microdissection of a fragmented clone (arrow marking a break in the mosaic competent of the clone) and mitochondrial genome sequencing from “disconnected” CCO-deficient regions (areas 1 and 3) show identical mtDNA mutations affecting components of the respiratory chain that would be consistent with measurable CCO: m.7059G<A (encoding MT-COI), m.7521G<A (encoding MT-TD) and C9532ins (encoding MT-CO3). These mutations were absent in the CCO-proficient region (area 2). (F) Disconnected clones are consistent with continuous proximal to distal streams, which can generate “pseudo-disconnections” as illustrated, due to small proximal “oscillation” of the clone path, which result in different parts of the streams flowing in different branches as the clone migrates distally.

Considering the spatial distribution of CCO-deficient patches along the ductal tree of the prostate, we found that marked cells were organized in narrow cohesive streams that start in the proximal duct, frequently span the entire proximal to distal axis, and remained of near-constant width (Figure 2A). Significantly, at ductal branching points, clonal streams either flow entirely into a single duct or become segregated, with progeny flowing into both ducts (Figures 2B and 2C). From direct quantitative measures, supported by theoretical modeling, we confirmed that this fate is solely dependent upon the positioning of the stream along the circumference at the bifurcation point and the relative circumference of the two ductal branches, arguing again that the flow pattern of the clones is representative of the rest of the tissue, as no biased selection is evident (Figure 2D). Invariable and proportionate segregation of streams at branch points is consistent with directional proximal to distal flow of cells without significant lateral expansion. Additionally, such behavior implies that during migration along the proximal to distal axis, lateral competition between progenitors must be minimal.

Probing the spatial origin of these cellular streams, we found that for the overwhelming majority of long-ranging clones, their proximal portion overlay the juxta-urethral main trunk of the prostate glandular unit (95%, n = 20/21; Figures 1D, 1E, and S2). Thus, based on directional flow, we reasoned that clonal patches of labeled cells must originate from a self-renewing stem cell population located in the most proximal portion of the main trunk of each glandular unit. Indeed, such organization is consistent with the enrichment of stem cells reported to occur in proximal ducts in animal prostate models (Tsujimura et al., 2002, Goto et al., 2006).

Although, in most instances, patches of CCO-deficient cells remained contiguous along the length of the branching glandular unit, occasional neighboring patches appeared to be disconnected along the proximal-distal axis (20%, n = 4/21) (Figure 1E). With stem cells localized at the proximal region, we posited that these disconnected long-ranging patches were clonally related, with disconnections resulting from small rotational adjustments or drifts causing streams to switch their course transiently at more distal branching points (Movie S2). To test this hypothesis, we performed mtDNA sequencing and confirmed that disconnected CCO-deficient patches were indeed clonally related (Figures 2E and 2F). Furthermore, we found no evidence of disconnected CCO-deficient patches in the main proximal trunk (Figure S2), suggesting that the observed clonal fragmentations seen further along the network are not a reflection of stem cells switching in and out of quiescence. However, we did observe a single example of a distal-only CCO-deficient patch (5%) (Figure 1F), explained potentially by the infrequent loss of stem cell activity related to an irreversible detachment of the clone from the niche.

### Multiplicity and Fate Behavior of Human Prostate Stem Cells

**Figure 3:**
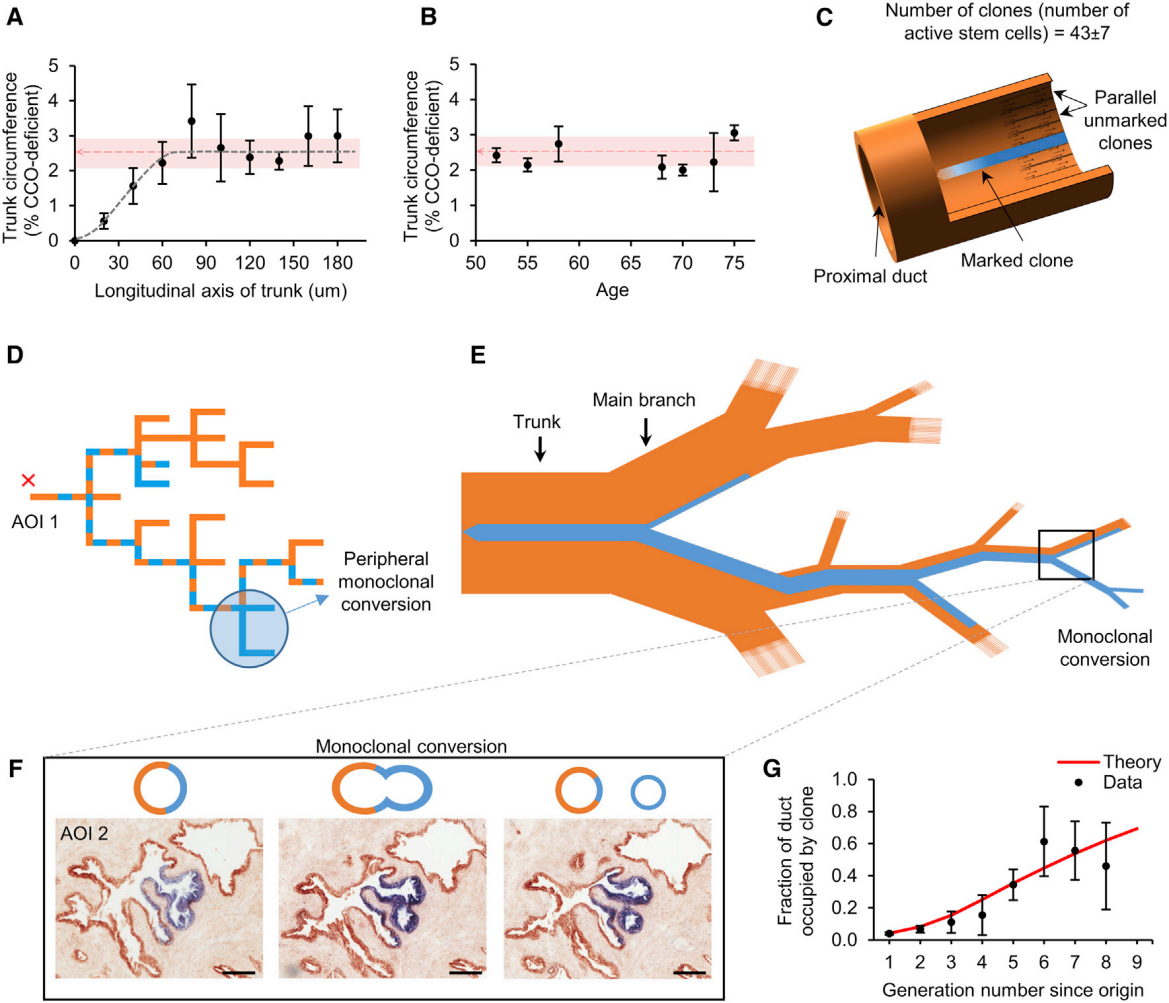
Multiplicity of Stem Cells and Peripheral Monoclonal Conversion (A) The ductal fraction occupied by a clonal stream saturates to a constant value along the main trunk length (n = 5 clones; n = 3 prostates; error bars represent SD; shaded area shows combined SD across all prostates). (B) The ductal fractions occupied by clonal streams were also relatively constant across patients of different ages (n = 7 clones; n = 7 prostates; error bars, SD). (C) A mean clone width of 2.4% of the total duct equates to 43 (37–50) circumferentially distributed active stem cells generating epithelial streams in cohesive longitudinal “laminar flow.” (D) Despite initial narrow ribbons of clonal streams in the main trunk, peripheral parts of a prostatic subunit can show entire monoclonal conversion. (E) Schematic describing the flow pattern of a clonal stream throughout the prostate and spatially restricted monoclonal enforcement. (F) Total clonal fraction is maintained before and after branching, but the ever-diminishing diameter of ducts proximally causes successive rounds of clonal enrichment in individual branches and thus monoclonal conversion. Scale bars, 100 μm. (G) Theoretic modeling of random redistribution of streams into identical daughter ducts mirrored the observed data, tending toward monoclonality peripherally as a function of increasing branch generation. Error bars, SD.

Next, we sought to determine the number of “functional” stem cells in a glandular unit and establish their mode of division. As clones exit the niche, stem cell progeny form a narrow ribbon of cells of near-constant width in proportion to the main trunk circumference of the glandular unit (Figure 3A). As other unmarked stem cells have the same dynamics and are predicted to produce streams of similar characteristics, we reasoned that the fraction of duct occupied by a given marked stream must be proportional to the fraction of proximal functional stem cells from which it is maintained (Lopez-Garcia et al., 2010). Strikingly, we found that this fraction was remarkably constant across patients of different ages and that the distribution of fractions was rather peaked around the average value (∼2.4% ± 0.4%; mean ± SD) (Figure 3B). These two observations provide further strong evidence both for CCO deficiency serving as a neutral marker and for minimal neutral drift caused by stem cell loss and replacement at the niche, as both would predict an increasing clone width as a function of patient age (Supplemental Experimental Procedures).

Combined with the minimal lateral dispersion of continuous streams along the proximal-distal axis, these results thus point at an asymmetric mode of stem cell division (Blanpain and Simons, 2013). In this paradigm, each migration stream is associated with a single functional stem cell, so that each glandular subunit must be supported by ∼43 ± 7 (mean ± SD) such stem cells lining the circumference of each proximal duct (Figure 3C).

Interestingly, although proximal sections of clones spanned only small fractions of ducts, in the distal region, clonal streams often occupied the entire ductal circumference, indicative of a monoclonal conversion process occurring along the ductal network (Figure 3D). At first sight, this behavior may seem at odds with the width of migration streams remaining approximately constant along individual ductal segments (i.e., an absence of cell competition). However, a transition of clonal patches toward monoclonality can derive passively as a result of longitudinal flow through serial branching events (Figure 3E). Indeed, as the epithelial volume of the parent duct and its clone is shared between daughter ducts throughout the glandular tree (Figure S3A), the relative contribution of the clonal stream is prediction to rise in a logistic manner until clones fully occupy individual ducts (Figure 3F). Based on this paradigm, analysis of the clonal data showed quantitative agreement with theoretical modeling of cohesive streams bifurcating randomly at successive branch points (Figure 3G). This behavior explains why substantial segmental volumes of the distal prostate progress to monoclonality and how entire peripheral epithelial domains may be maintained by a single functional stem cell.

### Identification of Bipotent Basal Stem Cells and Unipotent Luminal Stem Cells

**Figure 4:**
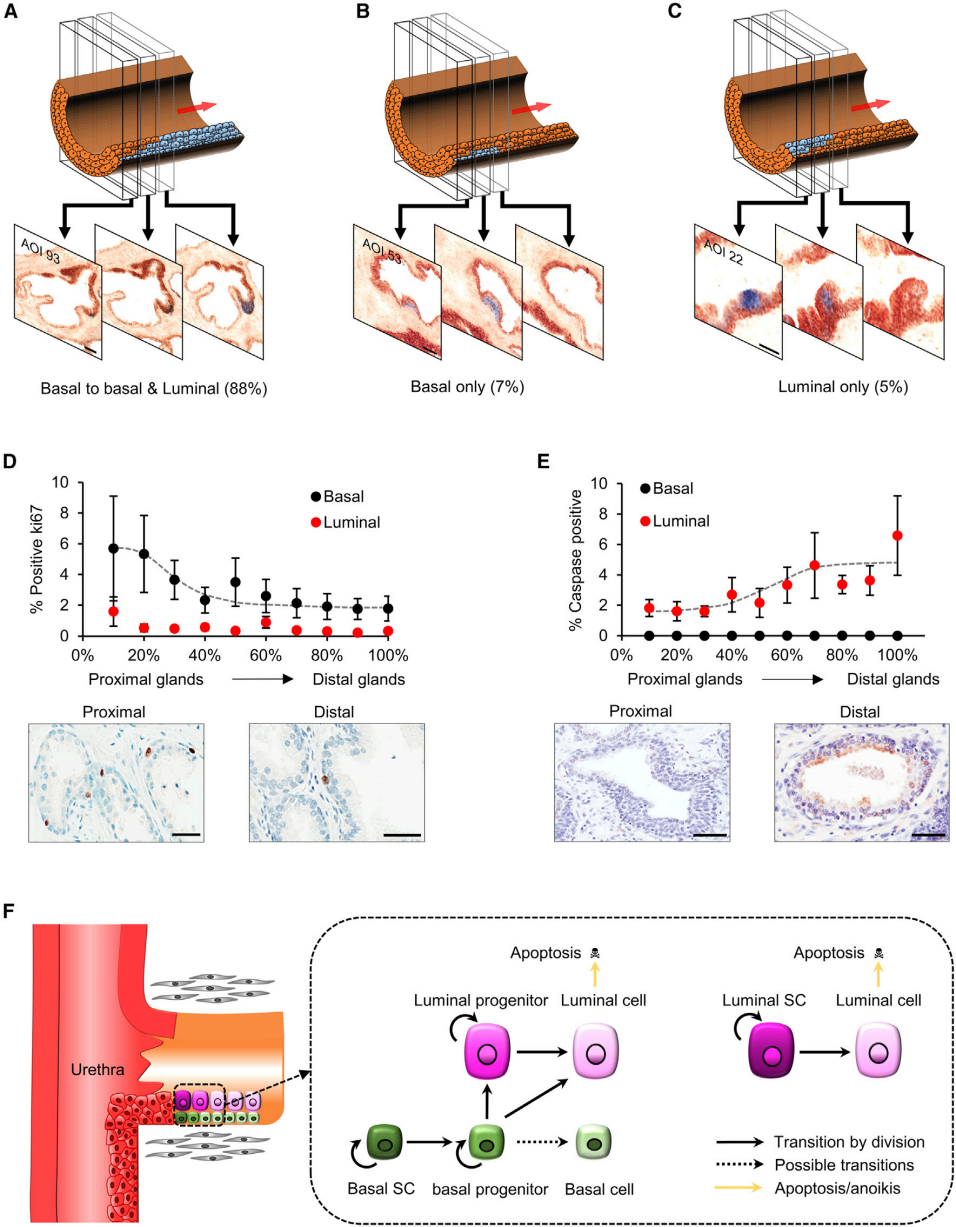
Clonal Mapping Reveals Proximal Multipotent Basal Stem Cells and Unipotent Luminal Stem Cells, as well as Basal Progenitors Located throughout the Prostate (A) Proximal clones in the truck start within the basal layer and then expand to basal and luminal compartments (n = 37/42). Scale bars, 50 μm. (B and C) Rarer patterns of clonal patch distribution exclusively in the basal (B; n = 3/42) (scale bars, 50 μm) or luminal layers (C; n = 2/42) were also observed and noted to be restricted to the proximal trunk. Scale bars, 50 μm. (D) Proliferation (Ki76) is predominantly confined to basal cells and displays a proximodistal gradient. Some proliferative activity remained in the distal regions, indicative of progenitor activity. Error bars, SD. Scale bars, 50 μm. (E) Cleaved caspase-3 measures showed apoptosis almost exclusively in the luminal compartment cells. Error bars, SD. Scale bars, 50 μm. (F) Proposed lineage hierarchy of homeostasis in the prostate. The progeny of proximal juxta-urethral basal stem cells generate a “conveyer belt” of migratory bipotent basal progenitors, which divide and differentiate to compensate the continual loss of luminal cells through apoptosis. A minor luminal unipotent stem cell pool maintains the most proximal luminal cells, proving only a small contribution to the entire duct, limited by the predominating apoptotic program of daughter luminal cells.

We next sought to define the fate of stem cell progeny to study the lineage hierarchy of basal and luminal cells. In particular, since cellular streams flow from the proximal to distal end of the glandular subunits, cells located more proximally are younger in the hierarchy, allowing the conversion of spatial into temporal information. In this way, we could assess the cell of origin of the cellular streams as well as the history of cell fate decisions. In the majority of cases (n = 37/42 of all proximal clones identified), we found that the most proximal portion of clones in the main trunk is initially restricted to the basal layer and only subsequently expands into both the basal and luminal layers (within 6–8 cell diameters along the proximal-distal axis) (Figure 4A). This observation suggests that adult prostatic epithelium is maintained by multipotent basal stem cells located in the most proximal region of the main trunk, giving rise to bipotent basal progenitors that produce luminal cells only later in the stream. To verify that this organization was specific to the stem cell niche, we examined the proximal boundary of distal disconnected clones and found indeed that distal clonal fragments always began as a bilayer (100%, n = 5) containing both basal and luminal cells (Figures S3B and S3C). Furthermore, a striking feature of the long clonal patches was the tightly overlapping spatial registration of the basal and luminal compartments throughout the clonal network (Figure S3C). This argues strongly against independent self-renewal of the basal and luminal fractions by lineage-restricted progenitors. Indeed, this conclusion was further reinforced by theoretical modeling, which showed that if basal and luminal compartments were maintained independently by symmetric divisions, clones would slowly diffuse out of register due to loss-replacement dynamics (Figure S3D).

Although examples of juxta-urethral small basal-only clonal patches (n = 3/42) were seen (Figure 4B), consistent with emerging clones having not yet expanded though the full thickness of the epithelium, it was notable that basal stem cells never gave rise to luminal cells in the most proximal regions of the prostate. In a complementary fashion, rare examples of luminal-only clonal patches in proximal trunks were identified (n = 2) (Figure 4C), which were invariably short (6–8 cell diameters along the proximal-distal axis) and were consistent with the existence of unipotent proximate luminal stem cells. However, these luminal stem cells make minor contributions to the epithelium volume and argue for the supremacy of multipotent basal stem cells in maintaining the bulk of prostate homeostasis.

To further characterize cellular heterogeneity, we correlated our clonal observations with kinetic measurements. Proliferative activity (assessed via ki67 staining) was predominantly localized to basal cells (Figure 4D) and most frequent in the proximal region while decaying increasingly slowly toward the distal region. Significantly, proliferative activity did not decay entirely to zero distally, indicating the existence of active basal progenitors along the entire proximal-distal axis of the glandular units. Interestingly, we found that the spatial patterning of apoptosis (assessed via activated caspase-3 staining) showed the opposite trend, increasing steadily along the proximal-distal axis, and was predominantly confined to the luminal layer (Figure 4E). These data strongly argue that the luminal compartment is not self-sustaining but is constantly renewed by the proliferation and differentiation of bipotent distal basal progenitors, which are themselves the product of multipotent proximal basal stem cells derived through streaming (Figure 4F).

### Delta Homolog 1 (DLK1) Enriches Human Prostate Basal Epithelial Stem Cells In Situ

**Figure 5:**
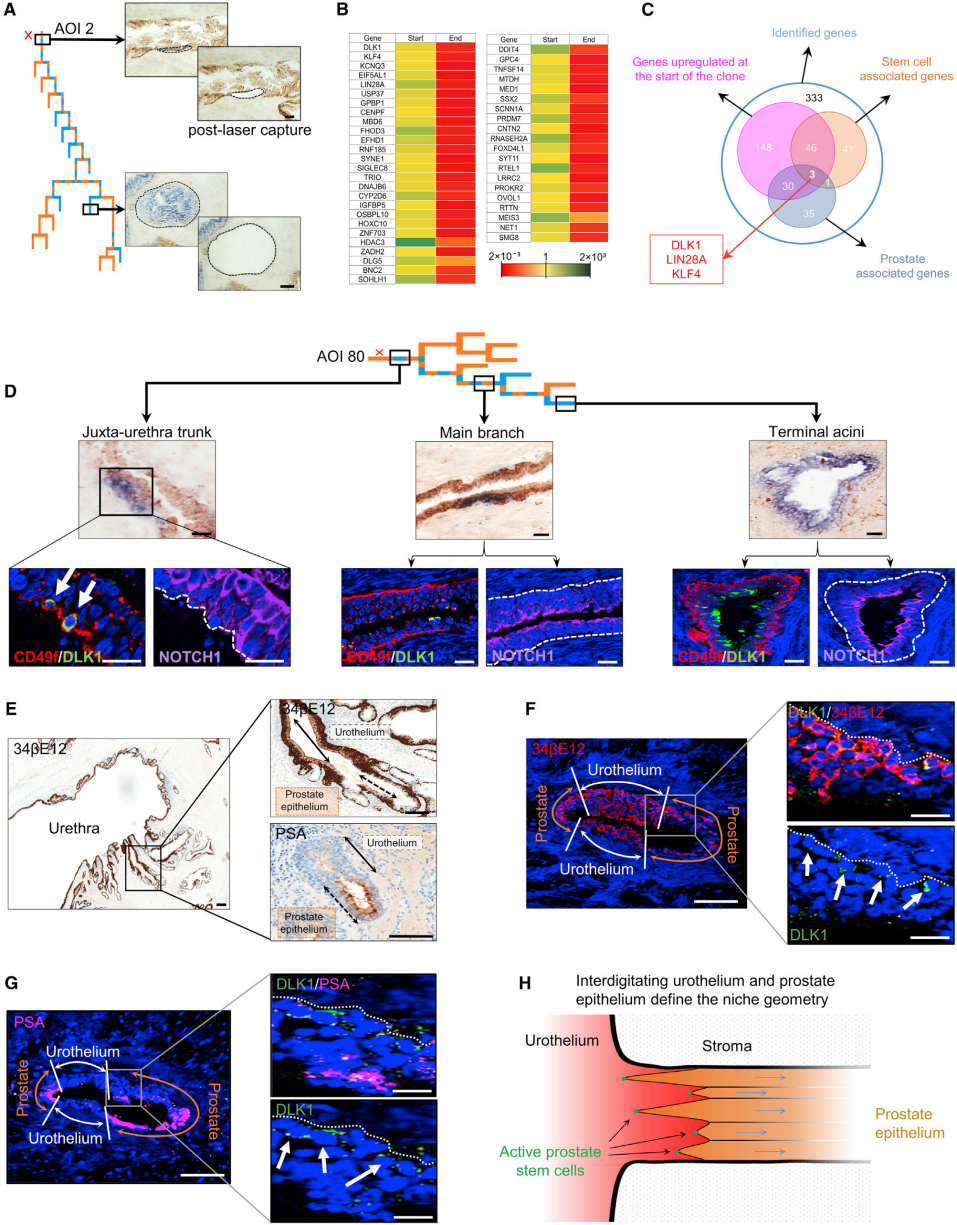
DLK1 Marks Basal Prostate Stem Cells and Defines Niche Microarchitecture In Situ (A) An example of focal cell laser capture from the proximal and distal end of a clonal patch. Scale bars, 50 μm. (B) Pooled RNA sequencing (n = 3) revealed that the proximal start of the clone is associated with marked upregulation of known stem cell markers, including a previously documented putative candidate marker for prostate stem cells (DLK1). (C) A systematic review of published literature of top upregulated gene expressions highlighted stem cell-pathway-associated markers in prostate studies. DLK1 is a cell-surface marker and was therefore selected as candidate for live-cell sorting. (D) Immunofluorescence of CD49f, DLK1, and NOTCH1 expression in juxta-urethra trunk (which co-localized with the start of CCO-deficient clone), an intermediate duct, and terminal acinus reveals distinct patterns of expression within basal and luminal cells. Dashed line indicates the epithelial basement membrane. Scale bars, 20 μm. (E) The juxta-urethral prostate ducts show variable encroachment of urothelium along the longitudinal axis, marked by 34betaE12 (expressed in all layers of urothelium but basal only in the prostate epithelium) and, in the next sequential slide in the z-plane, PSA (prostate luminal cells only). Scale bars, 100 μm. (F and G) Two consecutive sections of the same gland illustrate the urothelial-prostate epithelium boundary, described by (F) 34betaE12 and (G) PSA immunofluorescence in the radial axis, and demonstrate an interdigitating pattern on which DLK1^+ve^ basal prostate stem cells are positioned. Scale bars, 20 μm. (H) Sketch of the spatial arrangement of cells types at the niche (cross-section along the longitudinal axis of the proximal truck). Prostate stem cells are localized in between urethral and prostatic epithelial interdigitation, giving rise to transiently expanding clonal streams.

We then hypothesized that the capture of stem cells directly from their in situ niche domain would allow the identification of candidate markers to permit functional validation. We thus performed laser capture microdissection of the most proximal boundary of CCO-deficient patches to characterize the transcriptome of putative stem cells by comparative analysis with profiles from distal differentiated epithelium (Figure 5A). mRNA sequencing revealed upregulated expression of many previously described stem cell markers at the proximal boundary of clonal patches (Figure 5B; Table S1). Among the most highly upregulated transcripts was DLK1 (Figure 5C), which was previously reported as a putative human prostate stem cell marker in situ (Ceder et al., 2008). DLK1 encodes a cell-surface protein that serves as a “dead” ligand to Notch, a known regulator of homeostasis in the prostate epithelium (Wang et al., 2006, Valdez et al., 2012). Expression of DLK1 in putative basal stem cells was validated using immunofluorescence, demonstrating that the proximal boundary of clonal patches (n = 3) displayed focal DLK1 expression, which co-localized with α6-integrin (CD49f) expression, an established basal cell marker (Höfner et al., 2015, Drost et al., 2016) and associated Notch1 receptors (Figure 5D). Notch is an established regulator of basal progenitor differentiation (Valdez et al., 2012, Zhang et al., 2016), and the histological patterning of DLK1 and Notch1 revealed a distinct spatial expression profile in the niche, consistent with DLK1 providing an inhibitory signal to oppose basal progenitor differentiation. In contrast, DLK1 was expressed in luminal cells only in peripheral acini, where we had identified the greatest apoptotic activity, consistent with its role in the inhibition of the reported Notch-regulated resistance to cell death through anoikis in the luminal compartment (Kwon et al., 2014).

As these findings underline a close geographical connection between the stem cell niche and the interface of the urethra and prostate epithelium, we sought to characterize this boundary in further detail. Focal prostate basal epithelial DLK1 expression was situated at the transition boundary between prostate epithelium and urothelium (urethral epithelium) that typically extends from the urethra into the proximal prostatic ducts in an interdigitated fashion (Figures 5E–5G). The resultant summative “zigzag” pattern of prostate epithelial clone apices around the circumference of the duct, formed from initial lateral expansions of the clonal streams, is “backfilled” by the interdigitating encroachments of urothelium to define the histological geometry of the niche (Figure 5H).

### DLK1-Enriched Basal Epithelial Stem Cells Generate Fully Differentiated Prostate Gland Architecture

**Figure 6:**
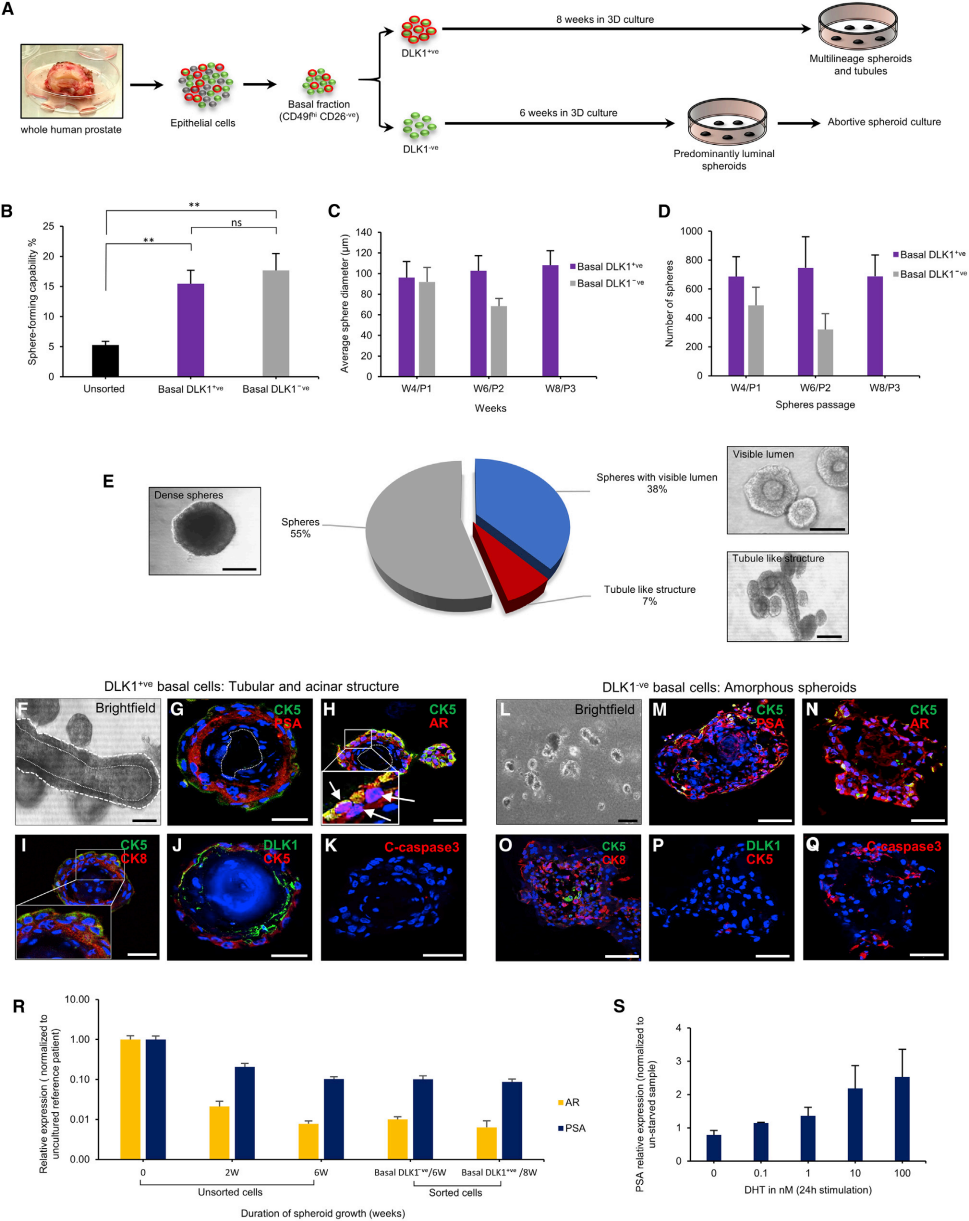
DLK1 Basal Cells Demonstrate Stem Cell Function by Generating Prostate Spheroids Ex Vivo (A) Schema outlining the 3D growth potential of selected cells from whole-prostate epithelium sorted into basal DLK1^+ve^ and DLK1^−ve^ populations. Sorted basal DLK1^−ve^ cells were viable for 6 weeks before becoming exhausted. (B) Sphere-forming capacity of whole-prostate basal DLK1^+ve^ and DLK1^−ve^ sorted cells, error bars, SEM (^∗∗^p ≤ 0.01). (C) Size of spheroid regeneration of basal cells through serial passage. Error bars, SEM. (D) Number of spheres from basal cells through serial passage; P1, first-generation spheroids; P2, second-generation spheroids; P3, third-generation spheroids. Error bars, SEM. (E) Histological patterning of DLK1^+ve^ basal cell-derived spheroids. Scale bars, 100 μm. (F–Q) Bright-field and immunofluorescence for DLK1, basal (CK5), luminal (PSA, AR, CK8), and apoptosis (cleaved caspase-3) markers for DLK1^+ve^ basal cells at 8 weeks, scale bars, 50 μm (F–K) and DLK1^−ve^ basal cells at 6 weeks (L–Q). Scale bars, 50 μm. (R) In the spheroids derived from unsorted cells and sorted fractions DLK1^−ve^ basal cells (6 weeks) and DLK1^+ve^ basal cells (8 weeks), AR mRNA was confirmed, albeit at low levels, and is associated with downstream readout of PSA expression. Error bars, SEM. (S) To further validate the functionality of AR, dose-dependent induction of PSA was demonstrated in transiently androgen-starved spheroid culture. Error bars, SEM.

To challenge the identity and multipotency of the basal stem cell compartment, we turned to 3D in vitro differentiation and serial culture assays. To ensure purity of cell selections, we used a robust set of established cell-surface markers for luminal (CD49f^lo/−^ CD26^+ve^) and basal (CD49f^hi^ CD26^−ve^) cells (Liu et al., 2004, Karthaus et al., 2014) (Figures 6A, S4, and S5). We chose “spheroid” (Garraway et al., 2010) over “organoid” (Karthaus et al., 2014) culture, as the former does not support differentiation from purified luminal cells, minimizing the potential for luminal cell contamination in sorted basal cells. A modified spheroid culture was employed with androgen (dihydrotestosterone [DHT] 10 nM) supplementation to support androgen receptor (AR)-regulated luminal differentiation from basal cells (Lang et al., 2001, Heer et al., 2007, Xin et al., 2007, Lamb et al., 2010, Lukacs et al., 2010). Both DLK1^+ve^ and DLK1^−ve^ basal cells were equally efficient at establishing first-generation spheroids, at an average rate of 15% ± 6% (Figure 6B). However, only spheres from the basal DLK1^+ve^ fraction could be serially passaged beyond 3 generations with sphere formation consistent with original founder generation in size and number (Figures 6C and 6D). In contrast, the size and numbers of spheres regenerated from the basal DLK1^−ve^ fraction diminished through serial passages before finally exhausting at 6–8 weeks of culture, consistent with a more limited self-renewal capacity of a purely progenitor population (Figure S6A).

DLK1^+ve^ basal cell-derived spheroids could be maintained beyond 8 weeks, mimicking both acinar-like and ductal-like structures, with visible lumens that only manifest after 6 weeks of culture (Figure 6E). Furthermore, DLK1^+ve^ basal cell derived spheroids at 8 weeks maintained renewal of DLK1^+ve^ cells and faithfully recapitulated the differentiated organization of prostate epithelium, in keeping with in situ histology composed of distinct polarization of epithelial layers expressing differentiation-specific markers for basal cells (CK5) and luminal cells (prostate specific antigen [PSA], AR, and CK8) (Figures 6F–6K and S6B–S6E). In common with spheroids obtained from DLK1^+ve^ cells, 3D cultures from DLK1^−ve^ basal cells generated amorphous spheroids up to 6 weeks but, in contrast, were unable to mature into histologically differentiated spheroids, as they became abortive with visible apoptosis (Figure 6L). At this point, spheroids derived from the DLK1^−ve^ basal cells were unable to recover basal cell (CK5) DLK1 expression and lost their original basal phenotype to predominantly express luminal markers (PSA, AR, and CK8) before exhaustion (Figures 6M–6Q). Partial AR nuclear localization was noted in spheroids from both basal cell fractions, consistent with previous reports using in vitro culture (Lang et al., 2001, Xin et al., 2007, Lamb et al., 2010), and functional nuclear localization was further confirmed with immunohistochemistry and PSA expression, in keeping with de facto terminally differentiated luminal cells (Figures 6R, 6S, and S6F–S6H).

## Discussion

In situ lineage tracing shows that the maintenance of adult prostate epithelium relies upon multipotent basal stem cells that occupy discrete niche domains positioned at the junction between proximal ducts and the urethra. These basal stem cells undergo a constant process of self-renewal, giving rise to bipotent basal progenitors that flow in coherent migration streams along the proximal-distal axis of ducts. As they migrate along the ductal network, basal progenitors self-renew, giving rise to luminal cells that replenish those lost continually through apoptosis. A second minority population of lineage-restricted stem cells replaces luminal cells lost in the close vicinity of the basal stem cell niche. Molecular profiling of cells captured by in situ laser microdissection identified DLK1 as a marker that overlaps with basal stem cells, a finding supported by spheroid culture assays. These findings contrast with studies of adult mice, where luminal and basal compartments are largely lineage restricted and locally maintained (Lawson et al., 2007, Wang et al., 2009, Lawson et al., 2010, Choi et al., 2012, Ousset et al., 2012).

Although the application of regeneration assays, which form the basis of previous human prostate studies, for homeostatic characterizations requires some caution (Donati and Watt, 2015, Tetteh et al., 2015), we note that xenograft recombination assays show a privileged capacity of basal-only cells to generate prostate-like grafts (Goldstein et al., 2008) and, in common with our in situ and in vitro differentiation assays, confirm basal cell derived luminal differentiation. However, in contrast to xenograft work, our in situ data also provide evidence for lineage-restricted luminal stem cells. Recent findings show that rare luminal cells can be grown in specialized ex vivo culture conditions (Karthaus et al., 2014), but, unlike our in situ observations of unipotency, these in vitro organoid studies reveal bipotency. These findings raise the potential of plasticity in luminal cell fate, which would not have been apparent in the current basal cell-focused spheroid culture. Further work is required to identify a marker for luminal stem cells in situ to better characterize their functional role in prostate regeneration.

The discovery of long-ranging epithelial flows emanating from discrete (closed) stem cell niche domains, organized in an interdigitating pattern at the juxta-urethral boundary, echoes the small intestine, where multiple crypts support single villi (Winton and Ponder, 1990, Barker et al., 2007, Lopez-Garcia et al., 2010, Snippert et al., 2010, Baker et al., 2014). Here, the epithelium is also maintained by discrete pockets of multipotent intestinal stem cells that localize to the base of crypts. In the course of turnover, these stem cells give rise to differentiating progeny that move in cohesive cell migration streams along the axis of the crypt and onto villi. The mechanical and molecular regulatory mechanisms that support such cohesive (non-mixing), directional, and long-ranged cellular flow patterns raise intriguing questions requiring further investigation. Whether each prostatic niche mimics the organization of the intestinal niche (Ritsma et al., 2014), playing host to multiple neutrally competing cells with stem cell potential, or whether each domain is associated with just a single stem cell anchored at the proximal tip remains undefined. Indeed, stem cell-derived clones that survive competition with neighbors will rapidly expand to fully occupy the niche, erasing information on the multiplicity of functional stem cells in each domain (niche succession). However, the rate of monoclonal conversion within each prostate niche would need to be sufficiently rapid for the resulting frequency of partially labeled proximal domains to lie beyond the resolution of the current assay and would account for the rare example of a distal-only CCO-deficient patch.

Beyond the closed-niche organization, the arrangement of the prostate epithelium into discrete long-ranging flows feeding sub-tree regions in a monoclonal fashion may provide insight into the concurrent observations of two frequent but apparently divergent phenomena of field characterization and inter-tumoral heterogeneity within cases of multifocal prostate cancer (Boyd et al., 2012, Boutros et al., 2015, Cooper et al., 2015). Given that long-lived, actively dividing stem cells are the cells most likely to accumulate age-related stochastic genomic DNA mutations (Alexandrov et al., 2015), the transmission of these mutations to progeny that clonally expand to occupy entire peripheral domains of the gland tree would account for the observed field change in cancer. These field changes could provide a substrate on which further mutations can act, leading to multifocal lesions once threshold events have accumulated. In the gastrointestinal tract, the segregation of epithelia into discrete glandular niche structures is thought to provide a measure of protection against the effects of deleterious mutations that confer a proliferative advantage on stem cells, inhibiting field change of the tissue. Mutant clones that derive from the transit-amplifying cell compartments may be “flushed” away through the constant turnover of tissue, while mutant clones derived from the resident stem cell compartment become restricted by the confines of the intestinal crypts. Whether the observed compartmentalization of the human prostate epithelium into discrete stem cell niches, segregated by tongues of urothelium analogous to the walls between adjacent intestinal crypts, provides the same degree of protection and, indeed, how the niche organization becomes disrupted or even subverted in the transition to neoplasia remains an interesting open question.

## Experimental Procedures

### Patient Samples

Whole clinically benign prostates from cystectomy surgery for bladder cancer were collected from the Freeman Hospital, Newcastle upon Tyne with appropriate ethical review, informed consent, and regulatory approvals (Newcastle REC 2003/11 and Human Tissue Authority License 12534) (Table S2).

### Whole-Prostate Sectioning and Marking of CCO-Deficient Epithelia

Whole clinically benign prostates were sectioned and every serial slide used for reconstruction of clonal patches was inspected by a clinical uropathologist to confirm normal histology (for further details, see Supplemental Experimental Procedures). Whole prostates were snap frozen and sequential CCO/succinate dehydrogenase (SDH) enzyme histochemistry was performed as previously described (Blackwood et al., 2011).

### Formal 3D Reconstruction of Clonal Mapping of CCO-Deficient Clones

Sections were scanned into the Aperio virtual pathology system (Leica Microsystems, UK) and then imported into Reconstruct (v1.1.0.0; GNU General Public License) to allow formal 3D reconstruction to accurately describe proximal to distal polarity of the complex ductal systems. In total, 42 long-ranging CCO-deficient clonal patches originating in the proximal duct were identified, and 21 were fully mapped into detailed 3D reconstructions from 12 whole-prostate samples.

### Laser Capture Microdissection (LCM) and RNA and mtDNA Sequencing

Areas of interest were captured using PALM MicroBeam laser micro-dissection microscope (Leica Microsystems, UK). Sequencing of the entire mitochondrial genome was undertaken using a two-stage amplification workflow, as previously described (Taylor et al., 2003). AROS Applied Biotechnology (Denmark) provided RNA sequencing using the Illumina HiSeq2500 platform. A Clontech Laboratories SMARTer (switching mechanism at 5′ end of RNA template, oligo dT primed) and PCR amplification of cDNA protocol was employed.

### Whole-Prostate Epithelia Extraction and FACS

Whole human prostate samples were rapidly processed to release epithelial cells as previously described (Heer et al., 2006). Single cells were labeled with as described in Supplemental Experimental Procedures and sorted using the BD fluorescence-activated cell sorting (FACS) ARIA II cell sorter and DIVA software for analysis (BD Biosciences, UK).

### 3D Spheroid Culture

Sorted epithelial cells were transferred to a 1:1 mixture of Matrigel (BD, growth factor reduced, phenol-red-free) and PrEGM (Lonza) (Goldstein et al., 2011), which was modified to support differentiation by supplementation with 10 nM DHT (replenished every 48 hr).

### Statistical Analyses

Data are expressed as means ± SEM. Statistical differences between experimental and control groups were determined by Student’s t test (unpaired, two tailed) unless otherwise specified. Excel was used for these statistical analyses. As further detailed in the Supplemental Experimental Procedures, least-squared fitting was used to deduce the rate of acquiring CCO-deficient mutations within prostatic sub-trees. The experimentally measured fractions of CCO-deficient epithelia per duct before and after bifurcation were used compute the probability of bifurcating into a large versus small duct or both and were compared to the analytical criteria derived in section 3.2 of the Supplemental Experimental Procedures.

## Author Contributions

R.H., R.P., R.W.T., and D.M.T. conceived the project. M.M., E.H., S.J.B., L.W., D.S., L.G., A.E., N.W., S.C.W., C.N.R., R.W.T., D.M.T., B.D.S., and R.H. designed the experiments. M.M., L.W., L.G., and S.C.W. performed the experiments. M.M., E.H., S.J.B., L.W., A.E., B.D.S., and R.H. performed data analysis. All authors participated in manuscript writing.

## Supplemental Information

Document S1. Supplemental Experimental Procedures and Figures S1–S7 and Table S2

Table S1. Differential Gene Expression, Related to Figure 5 A list of the genes from RNA sequencing (n=3) that upregulated at the proximal start of the clone, including a previously documented putative candidate marker for stem cells (LIN28A, and KLF4, and prostate stem cells (DLK1).

Movie S1. 3D Clone Reconstructions, Related to Figure 1 Workflow illustrating 3-D clone reconstructions and reduction to topographical maps.

Movie S2. “Pseudo-Disconnection,” Related to Figure 2 Diagrammatic cartoon of clone streams disconnections resulting from small rotational drifts. The movie shows how, over time, long ranging streams with fractional rotations culminating in sufficient marginal drift account for “pseudo-disconnection”. Subsequent temporal oscillation in the circumferential rotation can reroute the clone stream back into the original duct.

Document S2. Article plus Supplemental Information
