# Supplementary figures and images for: Multipotent Basal Stem Cells, Maintained in Localized Proximal Niches, Support Directed Long-Ranging Epithelial Flows in Human Prostates
Source: Cell Rep. 2017 Aug 15;20(7):1609–22. doi: 10.1016/j.celrep.2017.07.061 (PMC5565638; doi:10.1016/j.celrep.2017.07.061)

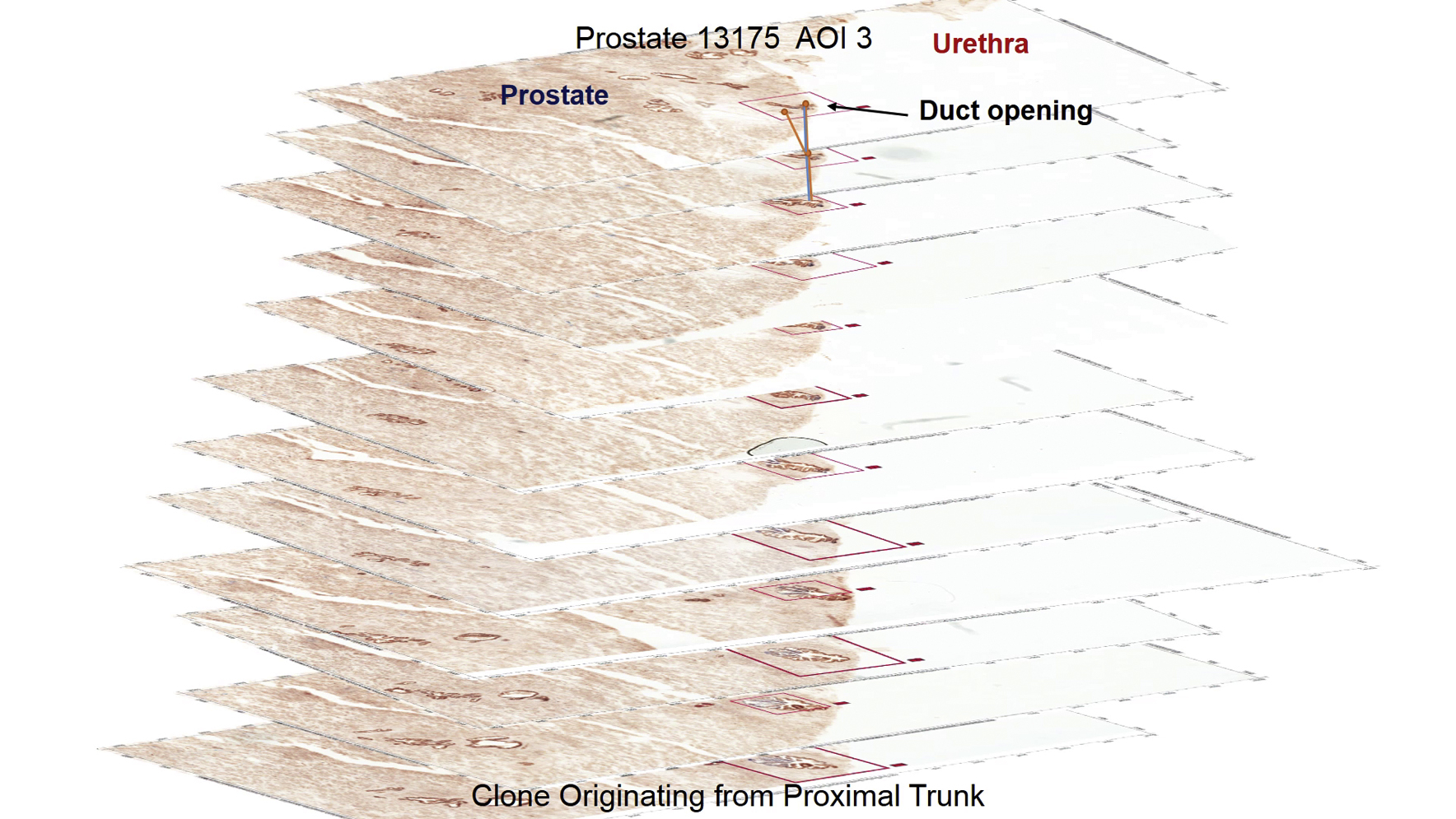

Supplement: Movie S1. 3D Clone Reconstructions, Related to Figure 1 — Workflow illustrating 3-D clone reconstructions and reduction to topographical maps. [file mmc3.jpg]

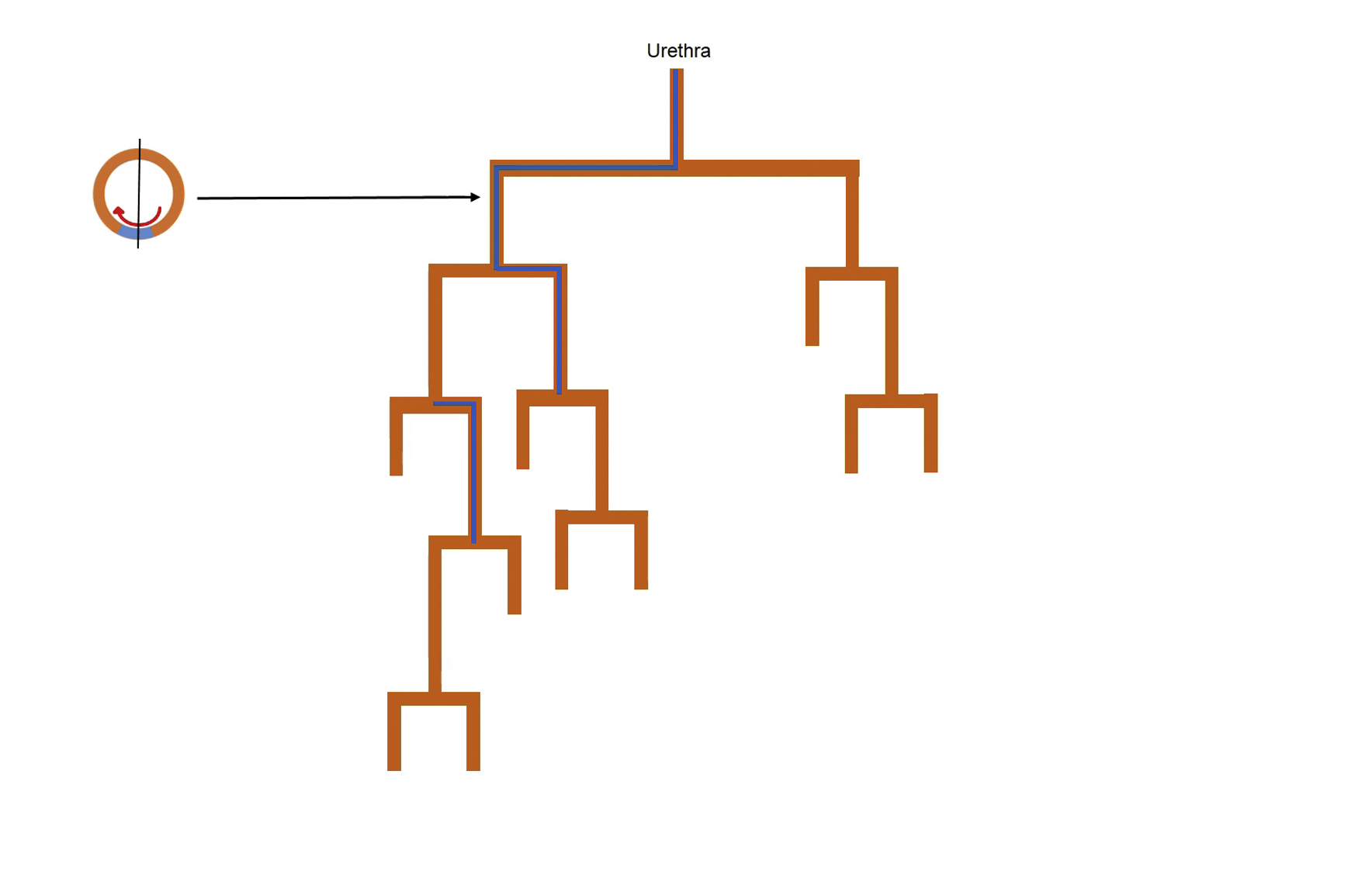

Supplement: Movie S2. “Pseudo-Disconnection,” Related to Figure 2 — Diagrammatic cartoon of clone streams disconnections resulting from small rotational drifts. The movie shows how, over time, long ranging streams with fractional rotations culminating in sufficient marginal drift account for “pseudo-disconnection”. Subsequent temporal oscillation in the circumferential rotation can reroute the clone stream back into the original duct. [file mmc4.jpg]
