# Supplementary material for: Multipotent Basal Stem Cells, Maintained in Localized Proximal Niches, Support Directed Long-Ranging Epithelial Flows in Human Prostates
Source: Cell Rep. 2017 Aug 15;20(7):1609–22. doi: 10.1016/j.celrep.2017.07.061 (PMC5565638; doi:10.1016/j.celrep.2017.07.061)
Supplement: Document S2. Article plus Supplemental Information [file mmc5.pdf]

Cell Reports

Article

Multipotent Basal Stem Cells, Maintained in Localized Proximal Niches, Support Directed Long-Ranging Epithelial Flows in Human Prostates

Graphical Abstract

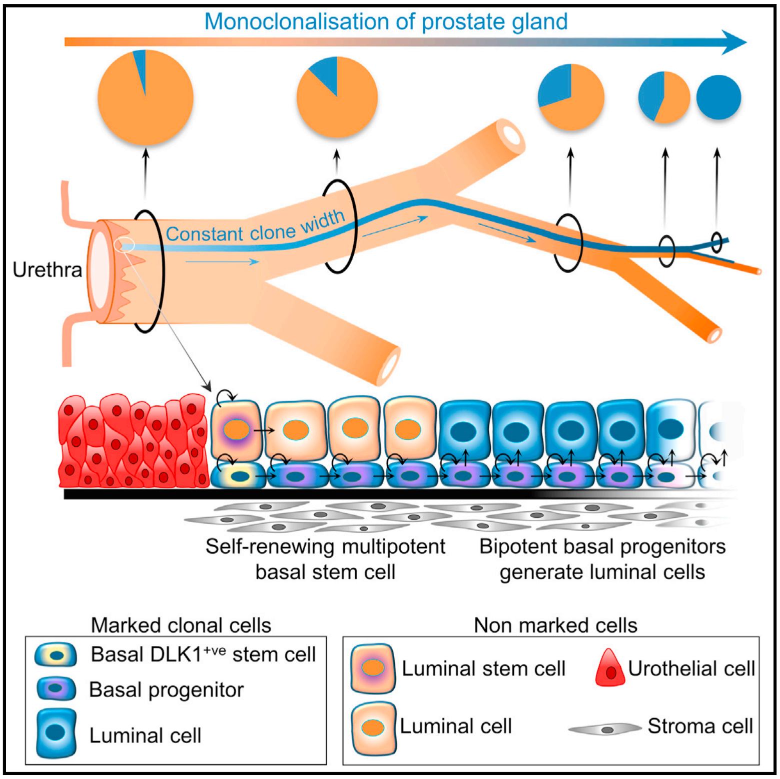

Highlights

- Discrete, proximal stem cell niche domains in juxta-urethral ducts
- Basal stem cells generate large-scale, directed, and cohesive cell migration streams
- Bipotent basal progenitors maintain the luminal compartment
- DLK1 marks stem cells in situ that recapitulate gland histology in 3D culture

Authors

Mohammad Moad, Edouard Hannezo, Simon J. Buczacki, ..., Craig N. Robson, Benjamin D. Simons, Rakesh Heer

Correspondence

bds10@cam.ac.uk (B.D.S.),
rakesh.heer@ncl.ac.uk (R.H.)

In Brief

Moad et al. find that multipotent prostate basal stem cells, marked by delta homolog 1 (DLK1), reside in proximal ducts and generate directed large-scale epithelial flows traversing the entire length of the branching gland network. This work describes mechanisms underlying 3D epithelial homeostasis in a complex branching tissue.

Moad et al., 2017, Cell Reports *20*, 1609–1622
August 15, 2017 © 2017 The Author(s).
http://dx.doi.org/10.1016/j.celrep.2017.07.061

OPEN ACCESS
CellPress

Multipotent Basal Stem Cells, Maintained in Localized Proximal Niches, Support Directed Long-Ranging Epithelial Flows in Human Prostates

Mohammad Moad,[1,14] Edouard Hannezo,[2,3,14] Simon J. Buczacki,[4] Laura Wilson,[1] Amira El-Sherif,[5,6] David Sims,[7] Robert Pickard,[8] Nicholas A. Wright,[9] Stuart C. Williamson,[10] Doug M. Turnbull,[11,12] Robert W. Taylor,[11] Laura Greaves,[11,12] Craig N. Robson,[1] Benjamin D. Simons,[2,3,13,*] and Rakesh Heer[1,15,*]

[1]Northern Institute for Cancer Research, Newcastle University, Newcastle upon Tyne NE2 4AD, UK
[2]Cavendish Laboratory, Department of Physics, University of Cambridge, J.J. Thomson Avenue, Cambridge CB3 0HE, UK
[3]Wellcome Trust/Cancer Research UK Gurdon Institute, University of Cambridge, Tennis Court Road, Cambridge CB2 1QN, UK
[4]Cancer Research UK, Cambridge Institute, University of Cambridge, Cambridge CB2 0RE, UK
[5]Department of Histopathology, Royal Victoria Infirmary, Newcastle upon Tyne NE1 4LP, UK
[6]Department of Pathology, Faculty of Medicine, Menoufia University, Menoufia, Egypt
[7]Computational Genomics Analysis and Training (CGAT), MRC Functional Genomics Unit, Department of Physiology, Anatomy and Genetics, University of Oxford, Oxford OX1 3PT, UK
[8]Institute of Cellular Medicine, Medical School, Newcastle University, Newcastle upon Tyne NE2 4HH, UK
[9]Barts Cancer Institute, Barts and the London School of Medicine and Dentistry, Queen Mary University of London, London EC1M 6BQ, UK
[10]Clinical and Experimental Pharmacology Group, University of Manchester, Manchester M13 9PL, UK
[11]Wellcome Trust Centre for Mitochondrial Research, Institute of Neuroscience, Newcastle University, Newcastle upon Tyne NE2 4HH, UK
[12]Newcastle Centre for Ageing and Vitality, Newcastle University, Newcastle upon Tyne NE2 4HH, UK
[13]Wellcome Trust/Medical Research Council Stem Cell Institute, Cambridge CB2 1QR, UK
[14]These authors contributed equally
[15]Lead Contact
*Correspondence: bds10@cam.ac.uk (B.D.S.), rakesh.heer@ncl.ac.uk (R.H.)
http://dx.doi.org/10.1016/j.celrep.2017.07.061

SUMMARY

Sporadic mitochondrial DNA mutations serve as clonal marks providing access to the identity and lineage potential of stem cells within human tissues. By combining quantitative clonal mapping with 3D reconstruction of adult human prostates, we show that multipotent basal stem cells, confined to discrete niches in juxta-urethral ducts, generate bipotent basal progenitors in directed epithelial migration streams. Basal progenitors are then dispersed throughout the entire glandular network, dividing and differentiating to replenish the loss of apoptotic luminal cells. Rare lineage-restricted luminal stem cells, and their progeny, are confined to proximal ducts and provide only minor contribution to epithelial homeostasis. In situ cell capture from clonal maps identified delta homolog 1 (DLK1) enrichment of basal stem cells, which was validated in functional spheroid assays. This study establishes significant insights into niche organization and function of prostate stem and progenitor cells, with implications for disease.

INTRODUCTION

The prostate consists of distinct glandular subunits that independently drain proximally into the prostatic urethra (McNeal, 1968) (Figure 1A). The glandular subunits comprise a complex branching ductal network of stratified epithelia composed of basal, luminal, and sparse neuroendocrine cells (Shen and Abate-Shen, 2010). In situ genetic labeling studies in mouse suggest that the adult prostate is largely maintained by the slow turnover of distinct lineage-restricted cytokeratin 5 (CK5)-expressing basal and CK8/CK18-expressing luminal cells (Choi et al., 2012; Ousset et al., 2012), while a minority of basal cells show evidence of bipotency (Lu et al., 2013; Wang et al., 2013). Lineage tracing studies of tissue regeneration, following cyclical androgen deprivation, also point to rare bipotent castration-resistant luminal stem cells (Wang et al., 2009, 2013; Chua et al., 2014). However, differences in both lifespan and histological organization in mice question the applicability of these findings to the human prostate, where the identity of stem cells is unknown (Huang and Witte, 2010).

In humans, bipotent basal progenitors have been primarily characterized by ex vivo selection of putative stem cell-enriching markers and a combination of in vitro culture and in vivo xenograft regeneration assays (Cunha, 1972; Xin et al., 2003; Richardson et al., 2004; Goldstein et al., 2008; Leong et al., 2008). However, rare bipotent human luminal cells are described in vitro, but not in xenograft assays (Goldstein et al., 2008; Karthaus et al., 2014). Although ex vivo regeneration assays were developed as a model to study homeostatic biology within the human prostate, emerging evidence suggests that cell behaviors during regeneration may be markedly different to normal physiology (Donati and Watt, 2015; Tetteh et al., 2015). However, in contrast to animal studies, the in situ lineage potential of human prostate cells is currently poorly defined.

This is an open access article under the CC BY license (http://creativecommons.org/licenses/by/4.0/).

OPEN ACCESS
CellPress

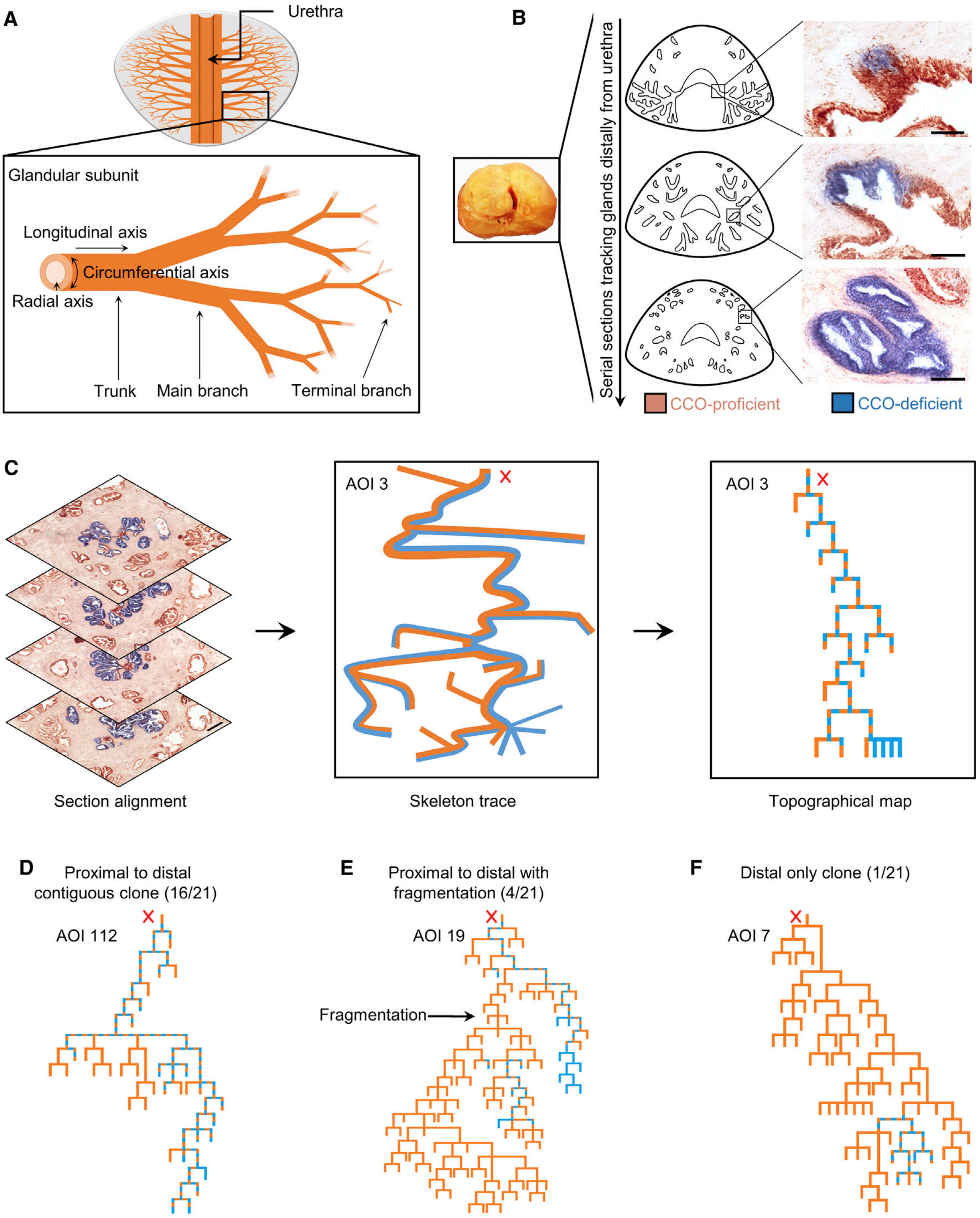

Figure 1. Transmission of mtDNA Identifies Long-Ranging Clones Spanning the Entire Prostate from Proximal Juxta-Urethra Ducts to Distal Acini

(A) The prostate comprises 12–18 paired glandular subunits independently draining into the urethra.

(B) Two-color enzyme histochemistry simultaneously detects activity of the mtDNA-encoded CCO and nuclear-DNA-encoded succinate dehydrogenase (SDH), with CCO-deficient cells appearing blue and CCO-proficient cells appearing brown. Scale bars, 50 μm.

(C) Serial sections are aligned in reconstruction software to generate a 3D wire-frame reconstruction of glandular subunit. The reconstruction is converted to a topographical representation to more clearly illustrate spread of the CCO-deficient patch through ductal epithelium (solid brown line represents homogeneous

(legend continued on next page)

Previously, using mtDNA mutations as a surrogate lineage tracing mark, we showed in vivo evidence for local cohesive clonal patches containing both basal and luminal cells, along with neuroendocrine cells (Blackwood et al., 2011). Whether this reflects multipotency of luminal and/or basal lineages remains unknown, while key questions concerning the existence, identity, and function of the stem cell compartment within the human prostate remain subject to conjecture. Here, using a combination of quantitative lineage tracing studies, 3D glandular reconstructions, proliferation kinetics, and functional assays of differentiation, we define the location, lineage potential, and functional behavior of stem cells and their progeny in the adult human prostate.

RESULTS

mtDNA Mutations Mark Long-Ranging Clones In Situ and Function as a Neutral Marker of Cells in the Human Prostate

To trace the cellular dynamics of in the human prostate, we employed a lineage labeling strategy based on the sporadic acquisition of mtDNA mutations (Taylor et al., 2003; Blackwood et al., 2011; Gaisa et al., 2011) using histocytochemical cytochrome *c* oxidase (CCO) deficiency as a reporter (Supplemental Experimental Procedures). 3D glandular reconstruction of the enzyme histochemistry using serial sections of entire human prostates characterized the topology of the epithelial branching network as well as the size and spatial organization of CCO-deficient clones (Figures 1B and 1C; Movie S1). Alongside small clonal patches (of 4–6 cell diameters), marking progenitor cell progeny that were seen to be dispersed sporadically and widely throughout the prostate (Blackwood et al., 2011; Gaisa et al., 2011), 3D glandular reconstructions revealed rare and large cohesive CCO-deficient patches, typically consisting of hundreds of thousands of cells and spanning entire individual glandular subunits (Figures 1D–1F).

To address the implications of such long-ranging clones, we first assessed whether mtDNA mutation serves as a neutral marker in the human prostate in light of previous studies raising concerns about a bias affecting cell fate through altered proliferation, differentiation, and apoptosis (Payne et al., 2005). Measuring both the proliferation and apoptosis rates of CCO-deficient and CCO-proficient epithelial cells, we found no statistically significant differences between them (Figures S1A and S1B). Moreover, CCO-deficient cells were present in both basal and luminal differentiated layers in a ratio statistically equivalent to that of the CCO-proficient epithelium (Figures S1C and S1D). Further evidence for the utility of CCO deficiency as a clonal tracer in prostate comes from the incidence of this mark within the gland. We found that the prostates examined were organized into 26 ± 2 (mean ± SD, n = 10 prostates) independent branching structures or subunits, as previously described (McNeal, 1968), which open separately into the urethra. The overwhelming majority (86% ± 4%) of prostate subunits did not contain extended CCO-deficient patches (Figures S1E and S1F), providing quantitative evidence that patches arise from discrete clonal events (Figure S1G; Supplemental Experimental Procedures). Moreover, older patients displayed a larger fraction of labeled subunits, in a manner quantitatively consistent with stochastic clonal induction occurring at a constant rate throughout the lifetime of the adult prostate.

Stem Cells Localized at the Proximal Junction of Glandular Units Give Rise to Progeny that Migrate in Coherent Steams along the Proximal-Distal Axis

The spatial organization and extension of labeled patches of cells along the proximal-distal axis question their origin. In principle, such an arrangement could derive from the competition and turnover of equipotent stem cells distributed throughout the prostate, leading to bidirectional expansion of labeled clones along the ducts. Alternatively, such clonal structures might derive from the unidirectional flow of migratory cells from a localized stem cell niche domain, analogous to that characterized in the intestinal crypt (Winton and Ponder, 1990; Barker et al., 2007; Lopez-Garcia et al., 2010; Snippert et al., 2010; Baker et al., 2014).

Considering the spatial distribution of CCO-deficient patches along the ductal tree of the prostate, we found that marked cells were organized in narrow cohesive streams that start in the proximal duct, frequently span the entire proximal to distal axis, and remained of near-constant width (Figure 2A). Significantly, at ductal branching points, clonal streams either flow entirely into a single duct or become segregated, with progeny flowing into both ducts (Figures 2B and 2C). From direct quantitative measures, supported by theoretical modeling, we confirmed that this fate is solely dependent upon the positioning of the stream along the circumference at the bifurcation point and the relative circumference of the two ductal branches, arguing again that the flow pattern of the clones is representative of the rest of the tissue, as no biased selection is evident (Figure 2D). Invariable and proportionate segregation of streams at branch points is consistent with directional proximal to distal flow of cells without significant lateral expansion. Additionally, such behavior implies that during migration along the proximal to distal axis, lateral competition between progenitors must be minimal.

Probing the spatial origin of these cellular streams, we found that for the overwhelming majority of long-ranging clones, their proximal portion overlay the juxta-urethral main trunk of the prostate glandular unit (95%, n = 20/21; Figures 1D, 1E, and S2). Thus, based on directional flow, we reasoned that clonal patches of labeled cells must originate from a self-renewing stem cell population located in the most proximal portion of the main trunk of each glandular unit. Indeed, such organization is consistent with the enrichment of stem cells reported to occur in proximal ducts in animal prostate models (Tsujimura et al., 2002; Goto et al., 2006).

CCO proficiency; blue-brown dashed line, mosaic CCO-deficiency; solid blue line, homogeneous CCO-deficiency). "X" marks common duct opening onto the urethra. Scale bar, 200 μm.

(D–F) Three types of clone pattern distributions were identified: long-ranging clones (95%) composed of proximal to distal contiguous patches (75%) (left), occasional proximal to distal clones with fragmentation (20%) (center), and a single rare example of a distal-only clone (5%) (right).

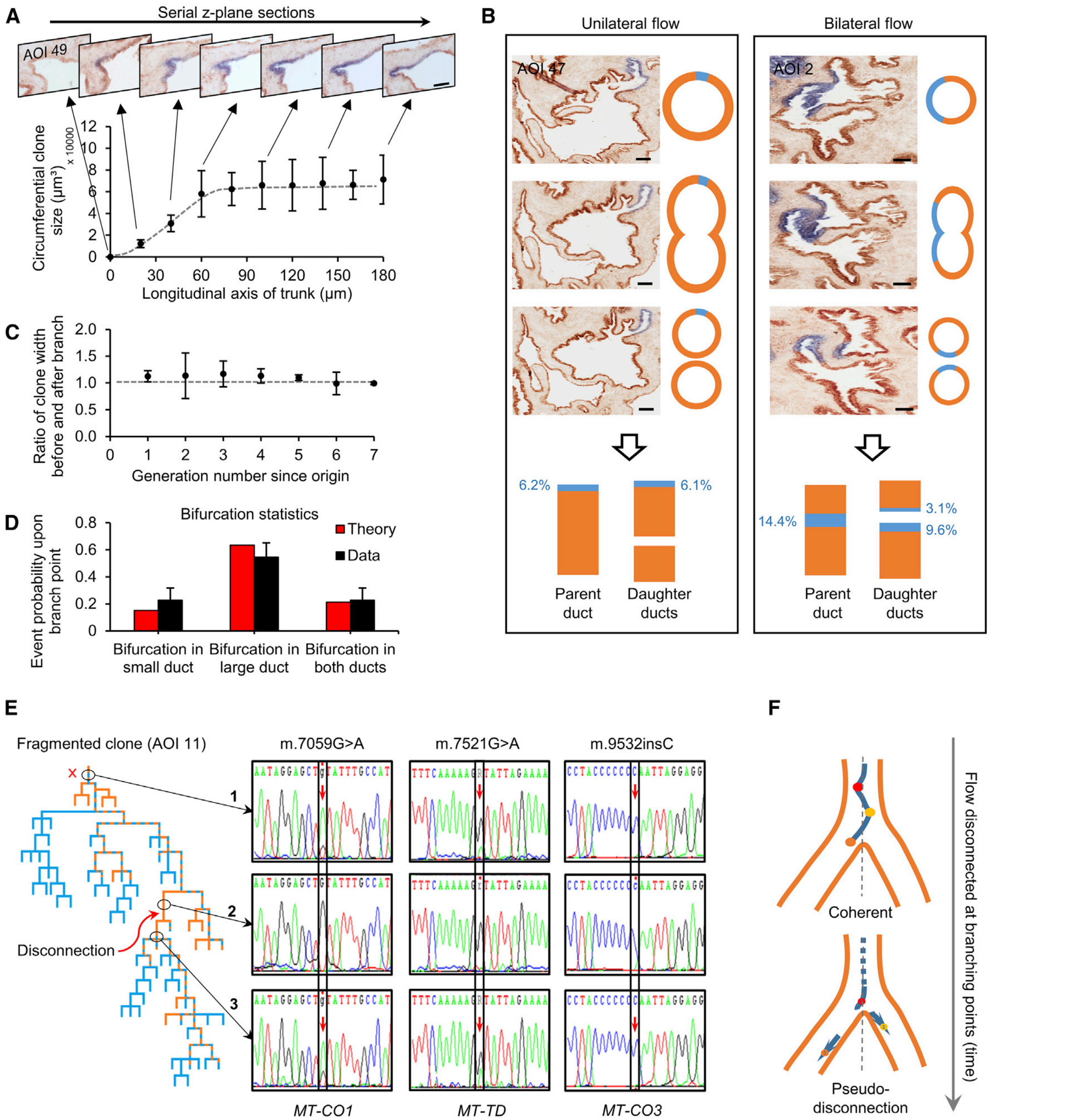

Figure 2. Directed Flow of Coherent Streams Reveals Proximal Juxta-Urethral Stem Cell Niche Domains

(A) Constant circumferential widths of individual clonal patches are generated in the common trunks following a short region of transient expansion (n = 5 clones; n = 3 prostates; error bars represent SEM). A representative histology "filmstrip" is shown, capturing the start of a CCO-deficient patch in sequential z-plane images. Scale bar, 100 µm.

(B) Two patterns of clone transmission are seen at duct branching, either unilateral or bilateral flow, with the clone width in parent duct always shared proportionately into the daughter ducts, consistent with directed flow. Scale bars, 100 µm.

(C) Proportion of clone width in parent duct compared with the proportion in daughter ducts is maintenance throughout the branching tree. A ratio of cumulative clone width before and after branching is presented. Error bars, SD.

(D) Passive and random flow of streams into daughter branches was theoretically modeled according to duct size and fraction of parent duct occupied by the duct. The predicted probabilities of outcome upon branching were consistent with observed data, showing that streams behave and branch neutrally.

(E) Laser capture microdissection of a fragmented clone (arrow marking a break in the mosaic competent of the clone) and mitochondrial genome sequencing from "disconnected" CCO-deficient regions (areas 1 and 3) show identical mtDNA mutations affecting components of the respiratory chain that would be

(legend continued on next page)

Although, in most instances, patches of CCO-deficient cells remained contiguous along the length of the branching glandular unit, occasional neighboring patches appeared to be disconnected along the proximal-distal axis (20%, n = 4/21) (Figure 1E). With stem cells localized at the proximal region, we posited that these disconnected long-ranging patches were clonally related, with disconnections resulting from small rotational adjustments or drifts causing streams to switch their course transiently at more distal branching points (Movie S2). To test this hypothesis, we performed mtDNA sequencing and confirmed that disconnected CCO-deficient patches were indeed clonally related (Figures 2E and 2F). Furthermore, we found no evidence of disconnected CCO-deficient patches in the main proximal trunk (Figure S2), suggesting that the observed clonal fragmentations seen further along the network are not a reflection of stem cells switching in and out of quiescence. However, we did observe a single example of a distal-only CCO-deficient patch (5%) (Figure 1F), explained potentially by the infrequent loss of stem cell activity related to an irreversible detachment of the clone from the niche.

Multiplicity and Fate Behavior of Human Prostate Stem Cells

Next, we sought to determine the number of "functional" stem cells in a glandular unit and establish their mode of division. As clones exit the niche, stem cell progeny form a narrow ribbon of cells of near-constant width in proportion to the main trunk circumference of the glandular unit (Figure 3A). As other unmarked stem cells have the same dynamics and are predicted to produce streams of similar characteristics, we reasoned that the fraction of duct occupied by a given marked stream must be proportional to the fraction of proximal functional stem cells from which it is maintained (Lopez-Garcia et al., 2010). Strikingly, we found that this fraction was remarkably constant across patients of different ages and that the distribution of fractions was rather peaked around the average value ($\sim$2.4% ± 0.4%; mean ± SD) (Figure 3B). These two observations provide further strong evidence both for CCO deficiency serving as a neutral marker and for minimal neutral drift caused by stem cell loss and replacement at the niche, as both would predict an increasing clone width as a function of patient age (Supplemental Experimental Procedures).

Combined with the minimal lateral dispersion of continuous streams along the proximal-distal axis, these results thus point at an asymmetric mode of stem cell division (Blanpain and Simons, 2013). In this paradigm, each migration stream is associated with a single functional stem cell, so that each glandular subunit must be supported by $\sim$43 ± 7 (mean ± SD) such stem cells lining the circumference of each proximal duct (Figure 3C).

Interestingly, although proximal sections of clones spanned only small fractions of ducts, in the distal region, clonal streams often occupied the entire ductal circumference, indicative of a monoclonal conversion process occurring along the ductal network (Figure 3D). At first sight, this behavior may seem at odds with the width of migration streams remaining approximately constant along individual ductal segments (i.e., an absence of cell competition). However, a transition of clonal patches toward monoclonality can derive passively as a result of longitudinal flow through serial branching events (Figure 3E). Indeed, as the epithelial volume of the parent duct and its clone is shared between daughter ducts throughout the glandular tree (Figure S3A), the relative contribution of the clonal stream is prediction to rise in a logistic manner until clones fully occupy individual ducts (Figure 3F). Based on this paradigm, analysis of the clonal data showed quantitative agreement with theoretical modeling of cohesive streams bifurcating randomly at successive branch points (Figure 3G). This behavior explains why substantial segmental volumes of the distal prostate progress to monoclonality and how entire peripheral epithelial domains may be maintained by a single functional stem cell.

Identification of Bipotent Basal Stem Cells and Unipotent Luminal Stem Cells

We next sought to define the fate of stem cell progeny to study the lineage hierarchy of basal and luminal cells. In particular, since cellular streams flow from the proximal to distal end of the glandular subunits, cells located more proximally are younger in the hierarchy, allowing the conversion of spatial into temporal information. In this way, we could assess the cell of origin of the cellular streams as well as the history of cell fate decisions. In the majority of cases (n = 37/42 of all proximal clones identified), we found that the most proximal portion of clones in the main trunk is initially restricted to the basal layer and only subsequently expands into both the basal and luminal layers (within 6–8 cell diameters along the proximal-distal axis) (Figure 4A). This observation suggests that adult prostatic epithelium is maintained by multipotent basal stem cells located in the most proximal region of the main trunk, giving rise to bipotent basal progenitors that produce luminal cells only later in the stream. To verify that this organization was specific to the stem cell niche, we examined the proximal boundary of distal disconnected clones and found indeed that distal clonal fragments always began as a bilayer (100%, n = 5) containing both basal and luminal cells (Figures S3B and S3C). Furthermore, a striking feature of the long clonal patches was the tightly overlapping spatial registration of the basal and luminal compartments throughout the clonal network (Figure S3C). This argues strongly against independent self-renewal of the basal and luminal fractions by lineage-restricted progenitors. Indeed, this conclusion was further reinforced by theoretical modeling, which showed that if basal and luminal compartments were maintained independently by symmetric divisions, clones would slowly diffuse out of register due to loss-replacement dynamics (Figure S3D).

Although examples of juxta-urethral small basal-only clonal patches (n = 3/42) were seen (Figure 4B), consistent with emerging clones having not yet expanded though the full

consistent with measurable CCO: m.7059G<A (encoding MT-COI), m.7521G<A (encoding MT-TD) and C9532ins (encoding MT-CO3). These mutations were absent in the CCO-proficient region (area 2).
(F) Disconnected clones are consistent with continuous proximal to distal streams, which can generate "pseudo-disconnections" as illustrated, due to small proximal "oscillation" of the clone path, which result in different parts of the streams flowing in different branches as the clone migrates distally.

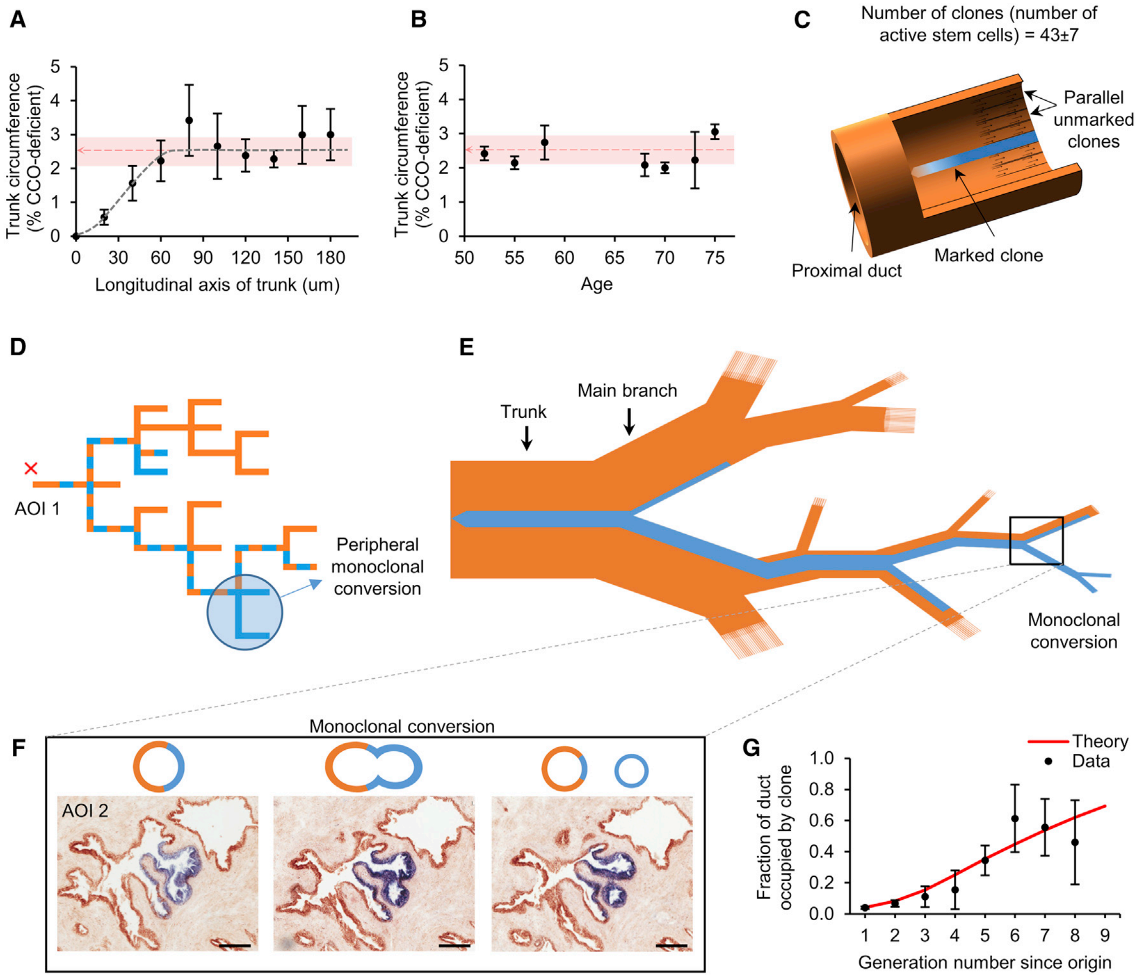

Figure 3. Multiplicity of Stem Cells and Peripheral Monoclonal Conversion

(A) The ductal fraction occupied by a clonal stream saturates to a constant value along the main trunk length (n = 5 clones; n = 3 prostates; error bars represent SD; shaded area shows combined SD across all prostates).

(B) The ductal fractions occupied by clonal streams were also relatively constant across patients of different ages (n = 7 clones; n = 7 prostates; error bars, SD).

(C) A mean clone width of 2.4% of the total duct equates to 43 (37–50) circumferentially distributed active stem cells generating epithelial streams in cohesive longitudinal "laminar flow."

(D) Despite initial narrow ribbons of clonal streams in the main trunk, peripheral parts of a prostatic subunit can show entire monoclonal conversion.

(E) Schematic describing the flow pattern of a clonal stream throughout the prostate and spatially restricted monoclonal enforcement.

(F) Total clonal fraction is maintained before and after branching, but the ever-diminishing diameter of ducts proximally causes successive rounds of clonal enrichment in individual branches and thus monoclonal conversion. Scale bars, 100 μm.

(G) Theoretic modeling of random redistribution of streams into identical daughter ducts mirrored the observed data, tending toward monoclonality peripherally as a function of increasing branch generation. Error bars, SD.

thickness of the epithelium, it was notable that basal stem cells never gave rise to luminal cells in the most proximal regions of the prostate. In a complementary fashion, rare examples of luminal-only clonal patches in proximal trunks were identified (n = 2) (Figure 4C), which were invariably short (6–8 cell diameters along the proximal-distal axis) and were consistent with the existence of unipotent proximate luminal stem cells. However, these luminal stem cells make minor contributions to the epithelium volume and argue for the supremacy of multipotent basal stem cells in maintaining the bulk of prostate homeostasis.

To further characterize cellular heterogeneity, we correlated our clonal observations with kinetic measurements. Proliferative activity (assessed via ki67 staining) was predominantly localized to basal cells (Figure 4D) and most frequent in the proximal region while decaying increasingly slowly toward the distal region. Significantly, proliferative activity did not decay entirely to zero distally, indicating the existence of active basal progenitors along the entire proximal-distal axis of the glandular units. Interestingly, we found that the spatial patterning of apoptosis (assessed via activated caspase-3 staining) showed the opposite trend, increasing steadily along the proximal-distal axis,

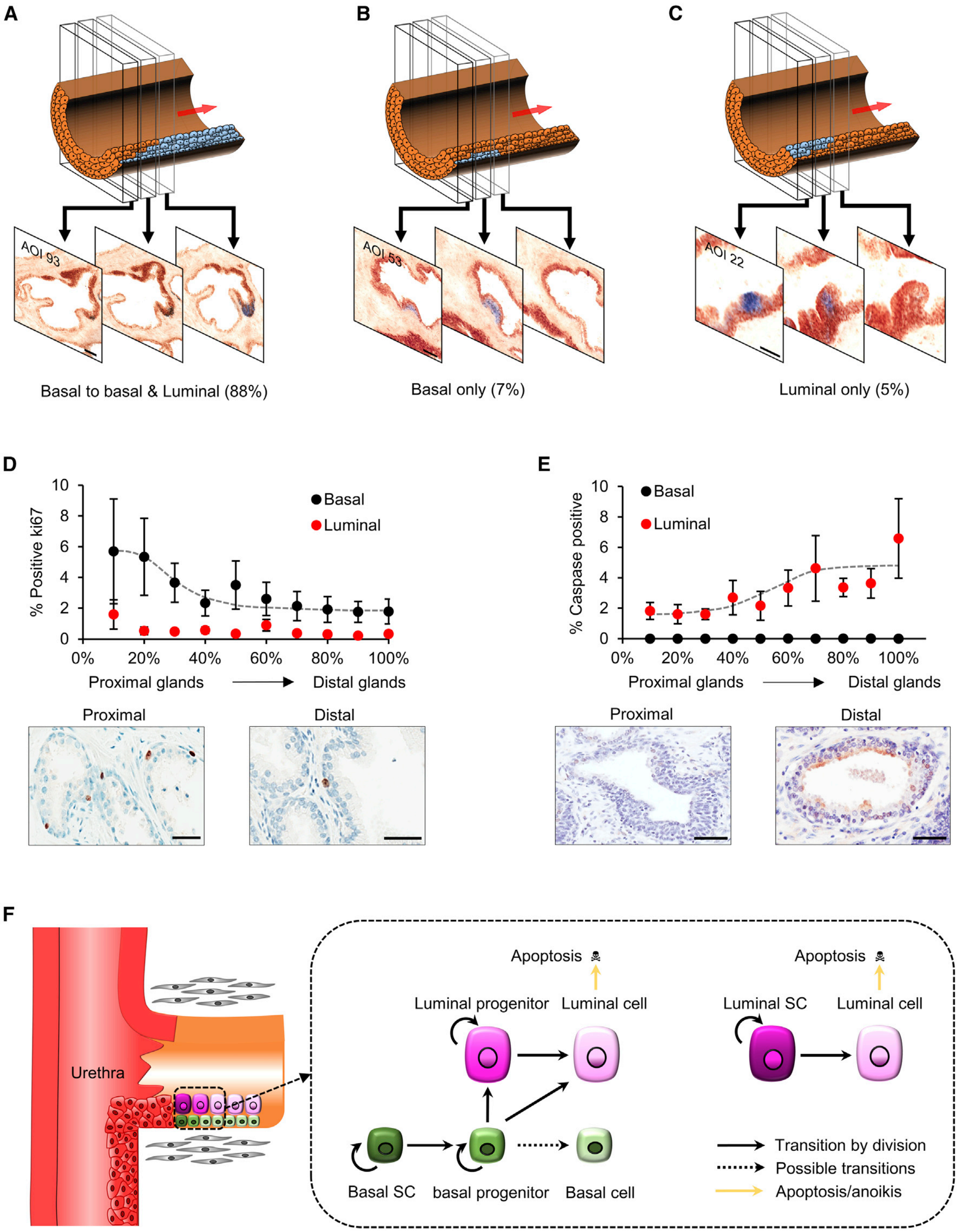

Figure 4. Clonal Mapping Reveals Proximal Multipotent Basal Stem Cells and Unipotent Luminal Stem Cells, as well as Basal Progenitors Located throughout the Prostate

(A) Proximal clones in the truck start within the basal layer and then expand to basal and luminal compartments (n = 37/42). Scale bars, 50 μm.

(B and C) Rarer patterns of clonal patch distribution exclusively in the basal (B; n = 3/42) (scale bars, 50 μm) or luminal layers (C; n = 2/42) were also observed and noted to be restricted to the proximal trunk. Scale bars, 50 μm.

(D) Proliferation (Ki76) is predominantly confined to basal cells and displays a proximodistal gradient. Some proliferative activity remained in the distal regions, indicative of progenitor activity. Error bars, SD. Scale bars, 50 μm.

(legend continued on next page)

and was predominantly confined to the luminal layer (Figure 4E). These data strongly argue that the luminal compartment is not self-sustaining but is constantly renewed by the proliferation and differentiation of bipotent distal basal progenitors, which are themselves the product of multipotent proximal basal stem cells derived through streaming (Figure 4F).

Delta Homolog 1 (DLK1) Enriches Human Prostate Basal Epithelial Stem Cells In Situ

We then hypothesized that the capture of stem cells directly from their in situ niche domain would allow the identification of candidate markers to permit functional validation. We thus performed laser capture microdissection of the most proximal boundary of CCO-deficient patches to characterize the transcriptome of putative stem cells by comparative analysis with profiles from distal differentiated epithelium (Figure 5A). mRNA sequencing revealed upregulated expression of many previously described stem cell markers at the proximal boundary of clonal patches (Figure 5B; Table S1). Among the most highly upregulated transcripts was DLK1 (Figure 5C), which was previously reported as a putative human prostate stem cell marker in situ (Ceder et al., 2008). DLK1 encodes a cell-surface protein that serves as a "dead" ligand to Notch, a known regulator of homeostasis in the prostate epithelium (Wang et al., 2006; Valdez et al., 2012). Expression of DLK1 in putative basal stem cells was validated using immunofluorescence, demonstrating that the proximal boundary of clonal patches (n = 3) displayed focal DLK1 expression, which co-localized with α6-integrin (CD49f) expression, an established basal cell marker (Höfner et al., 2015; Drost et al., 2016) and associated Notch1 receptors (Figure 5D). Notch is an established regulator of basal progenitor differentiation (Valdez et al., 2012; Zhang et al., 2016), and the histological patterning of DLK1 and Notch1 revealed a distinct spatial expression profile in the niche, consistent with DLK1 providing an inhibitory signal to oppose basal progenitor differentiation. In contrast, DLK1 was expressed in luminal cells only in peripheral acini, where we had identified the greatest apoptotic activity, consistent with its role in the inhibition of the reported Notch-regulated resistance to cell death through anoikis in the luminal compartment (Kwon et al., 2014).

As these findings underline a close geographical connection between the stem cell niche and the interface of the urethra and prostate epithelium, we sought to characterize this boundary in further detail. Focal prostate basal epithelial DLK1 expression was situated at the transition boundary between prostate epithelium and urothelium (urethral epithelium) that typically extends from the urethra into the proximal prostatic ducts in an interdigitated fashion (Figures 5E–5G). The resultant summative "zigzag" pattern of prostate epithelial clone apices around the circumference of the duct, formed from initial lateral expansions of the clonal streams, is "backfilled" by the interdigitating encroachments of urothelium to define the histological geometry of the niche (Figure 5H).

DLK1-Enriched Basal Epithelial Stem Cells Generate Fully Differentiated Prostate Gland Architecture

To challenge the identity and multipotency of the basal stem cell compartment, we turned to 3D in vitro differentiation and serial culture assays. To ensure purity of cell selections, we used a robust set of established cell-surface markers for luminal ($CD49f^{lo/-}$ $CD26^{+ve}$) and basal ($CD49f^{hi}$ $CD26^{-ve}$) cells (Liu et al., 2004; Karthaus et al., 2014) (Figures 6A, S4, and S5). We chose "spheroid" (Garraway et al., 2010) over "organoid" (Karthaus et al., 2014) culture, as the former does not support differentiation from purified luminal cells, minimizing the potential for luminal cell contamination in sorted basal cells. A modified spheroid culture was employed with androgen (dihydrotestosterone [DHT] 10 nM) supplementation to support androgen receptor (AR)-regulated luminal differentiation from basal cells (Lang et al., 2001; Heer et al., 2007; Xin et al., 2007; Lamb et al., 2010; Lukacs et al., 2010). Both $DLK1^{+ve}$ and $DLK1^{-ve}$ basal cells were equally efficient at establishing first-generation spheroids, at an average rate of 15% ± 6% (Figure 6B). However, only spheres from the basal $DLK1^{+ve}$ fraction could be serially passaged beyond 3 generations with sphere formation consistent with original founder generation in size and number (Figures 6C and 6D). In contrast, the size and numbers of spheres regenerated from the basal $DLK1^{-ve}$ fraction diminished through serial passages before finally exhausting at 6–8 weeks of culture, consistent with a more limited self-renewal capacity of a purely progenitor population (Figure S6A).

$DLK1^{+ve}$ basal cell-derived spheroids could be maintained beyond 8 weeks, mimicking both acinar-like and ductal-like structures, with visible lumens that only manifest after 6 weeks of culture (Figure 6E). Furthermore, $DLK1^{+ve}$ basal cell derived spheroids at 8 weeks maintained renewal of $DLK1^{+ve}$ cells and faithfully recapitulated the differentiated organization of prostate epithelium, in keeping with in situ histology composed of distinct polarization of epithelial layers expressing differentiation-specific markers for basal cells (CK5) and luminal cells (prostate specific antigen [PSA], AR, and CK8) (Figures 6F–6K and S6B–S6E). In common with spheroids obtained from $DLK1^{+ve}$ cells, 3D cultures from $DLK1^{-ve}$ basal cells generated amorphous spheroids up to 6 weeks but, in contrast, were unable to mature into histologically differentiated spheroids, as they became abortive with visible apoptosis (Figure 6L). At this point, spheroids derived from the $DLK1^{-ve}$ basal cells were unable to recover basal cell (CK5) DLK1 expression and lost their original basal phenotype to predominantly express luminal markers (PSA, AR, and CK8) before exhaustion (Figures 6M–6Q). Partial AR nuclear localization was noted in spheroids from both basal cell fractions, consistent with previous reports using in vitro culture (Lang et al., 2001; Xin et al., 2007; Lamb et al., 2010), and functional

(E) Cleaved caspase-3 measures showed apoptosis almost exclusively in the luminal compartment cells. Error bars, SD. Scale bars, 50 μm.

(F) Proposed lineage hierarchy of homeostasis in the prostate. The progeny of proximal juxta-urethral basal stem cells generate a "conveyer belt" of migratory bipotent basal progenitors, which divide and differentiate to compensate the continual loss of luminal cells through apoptosis. A minor luminal unipotent stem cell pool maintains the most proximal luminal cells, proving only a small contribution to the entire duct, limited by the predominating apoptotic program of daughter luminal cells.

OPEN
ACCESS
CellPress

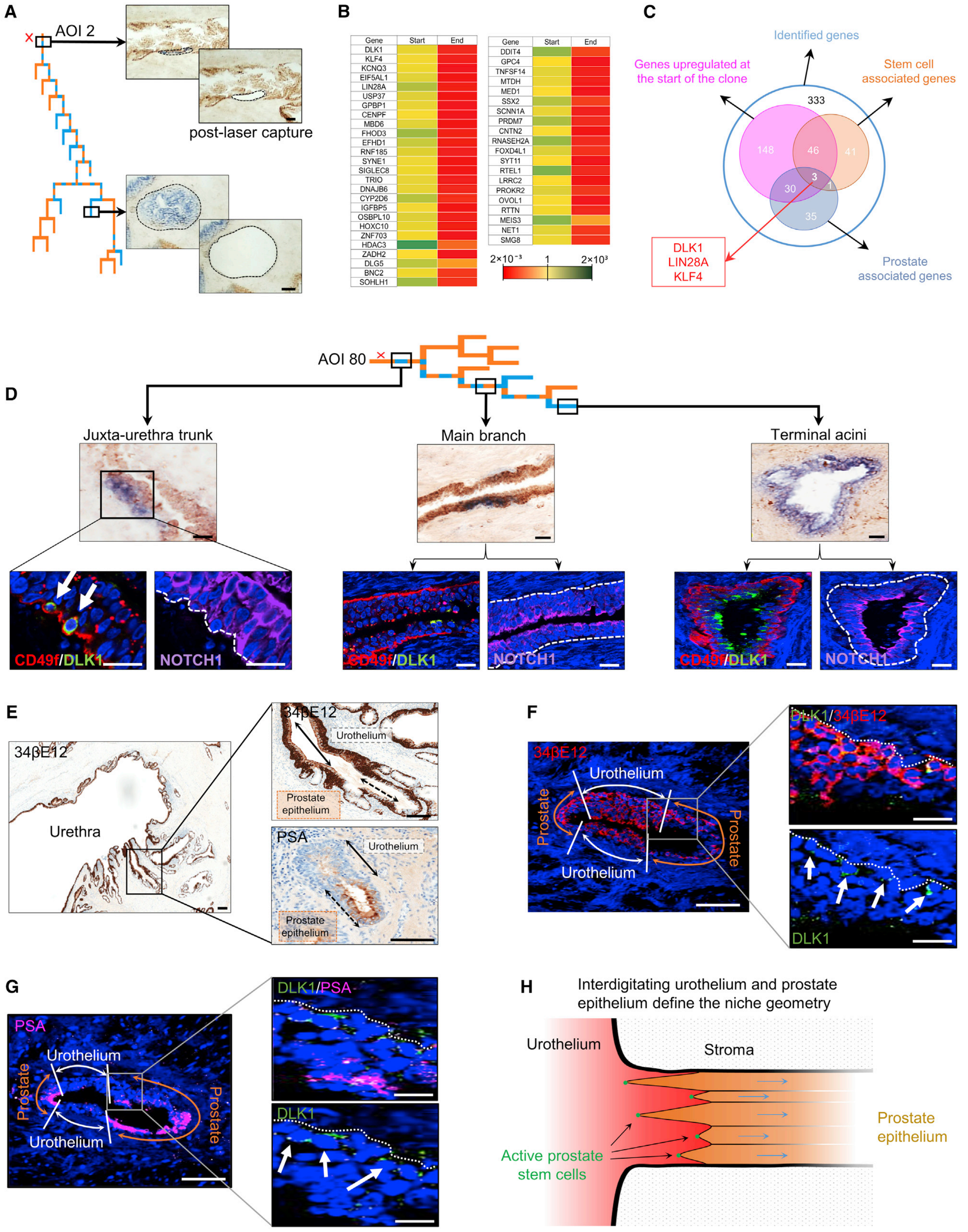

(legend on next page)

nuclear localization was further confirmed with immunohistochemistry and PSA expression, in keeping with de facto terminally differentiated luminal cells (Figures 6R, 6S, and S6F–S6H).

DISCUSSION

In situ lineage tracing shows that the maintenance of adult prostate epithelium relies upon multipotent basal stem cells that occupy discrete niche domains positioned at the junction between proximal ducts and the urethra. These basal stem cells undergo a constant process of self-renewal, giving rise to bipotent basal progenitors that flow in coherent migration streams along the proximal-distal axis of ducts. As they migrate along the ductal network, basal progenitors self-renew, giving rise to luminal cells that replenish those lost continually through apoptosis. A second minority population of lineage-restricted stem cells replaces luminal cells lost in the close vicinity of the basal stem cell niche. Molecular profiling of cells captured by in situ laser microdissection identified DLK1 as a marker that overlaps with basal stem cells, a finding supported by spheroid culture assays. These findings contrast with studies of adult mice, where luminal and basal compartments are largely lineage restricted and locally maintained (Lawson et al., 2007; Wang et al., 2009; Lawson et al., 2010; Choi et al., 2012; Ousset et al., 2012).

Although the application of regeneration assays, which form the basis of previous human prostate studies, for homeostatic characterizations requires some caution (Donati and Watt, 2015; Tetteh et al., 2015), we note that xenograft recombination assays show a privileged capacity of basal-only cells to generate prostate-like grafts (Goldstein et al., 2008) and, in common with our in situ and in vitro differentiation assays, confirm basal cell derived luminal differentiation. However, in contrast to xenograft work, our in situ data also provide evidence for lineage-restricted luminal stem cells. Recent findings show that rare luminal cells can be grown in specialized ex vivo culture conditions (Karthaus et al., 2014), but, unlike our in situ observations of unipotency, these in vitro organoid studies reveal bipotency. These findings raise the potential of plasticity in luminal cell fate, which would not have been apparent in the current basal cell-focused spheroid culture. Further work is required to identify a marker for luminal stem cells in situ to better characterize their functional role in prostate regeneration.

The discovery of long-ranging epithelial flows emanating from discrete (closed) stem cell niche domains, organized in an interdigitating pattern at the juxta-urethral boundary, echoes the small intestine, where multiple crypts support single villi (Winton and Ponder, 1990; Barker et al., 2007; Lopez-Garcia et al., 2010; Snippert et al., 2010; Baker et al., 2014). Here, the epithelium is also maintained by discrete pockets of multipotent intestinal stem cells that localize to the base of crypts. In the course of turnover, these stem cells give rise to differentiating progeny that move in cohesive cell migration streams along the axis of the crypt and onto villi. The mechanical and molecular regulatory mechanisms that support such cohesive (non-mixing), directional, and long-ranged cellular flow patterns raise intriguing questions requiring further investigation. Whether each prostatic niche mimics the organization of the intestinal niche (Ritsma et al., 2014), playing host to multiple neutrally competing cells with stem cell potential, or whether each domain is associated with just a single stem cell anchored at the proximal tip remains undefined. Indeed, stem cell-derived clones that survive competition with neighbors will rapidly expand to fully occupy the niche, erasing information on the multiplicity of functional stem cells in each domain (niche succession). However, the rate of monoclonal conversion within each prostate niche would need to be sufficiently rapid for the resulting frequency of partially labeled proximal domains to lie beyond the resolution of the current assay and would account for the rare example of a distal-only CCO-deficient patch.

Beyond the closed-niche organization, the arrangement of the prostate epithelium into discrete long-ranging flows feeding sub-tree regions in a monoclonal fashion may provide insight into the concurrent observations of two frequent but apparently divergent phenomena of field characterization and inter-tumoral heterogeneity within cases of multifocal prostate cancer (Boyd et al., 2012; Boutros et al., 2015; Cooper et al., 2015). Given that long-lived, actively dividing stem cells are the cells most likely to accumulate age-related stochastic genomic DNA mutations (Alexandrov et al., 2015), the transmission of these mutations to progeny that clonally expand to occupy entire peripheral domains of the gland tree would account for the observed field change in cancer. These field changes could provide a substrate on which further mutations can act, leading to multifocal lesions once threshold events have accumulated. In the gastrointestinal tract, the segregation of epithelia into discrete glandular niche

Figure 5. DLK1 Marks Basal Prostate Stem Cells and Defines Niche Microarchitecture In Situ

(A) An example of focal cell laser capture from the proximal and distal end of a clonal patch. Scale bars, 50 μm.

(B) Pooled RNA sequencing (n = 3) revealed that the proximal start of the clone is associated with marked upregulation of known stem cell markers, including a previously documented putative candidate marker for prostate stem cells (DLK1).

(C) A systematic review of published literature of top upregulated gene expressions highlighted stem cell-pathway-associated markers in prostate studies. DLK1 is a cell-surface marker and was therefore selected as candidate for live-cell sorting.

(D) Immunofluorescence of CD49f, DLK1, and NOTCH1 expression in juxta-urethra trunk (which co-localized with the start of CCO-deficient clone), an intermediate duct, and terminal acinus reveals distinct patterns of expression within basal and luminal cells. Dashed line indicates the epithelial basement membrane. Scale bars, 20 μm.

(E) The juxta-urethral prostate ducts show variable encroachment of urothelium along the longitudinal axis, marked by 34betaE12 (expressed in all layers of urothelium but basal only in the prostate epithelium) and, in the next sequential slide in the z-plane, PSA (prostate luminal cells only). Scale bars, 100 μm.

(F and G) Two consecutive sections of the same gland illustrate the urothelial-prostate epithelium boundary, described by (F) 34betaE12 and (G) PSA immunofluorescence in the radial axis, and demonstrate an interdigitating pattern on which DLK1^{+ve} basal prostate stem cells are positioned. Scale bars, 20 μm.

(H) Sketch of the spatial arrangement of cells types at the niche (cross-section along the longitudinal axis of the proximal truck). Prostate stem cells are localized in between urethral and prostatic epithelial interdigitation, giving rise to transiently expanding clonal streams.

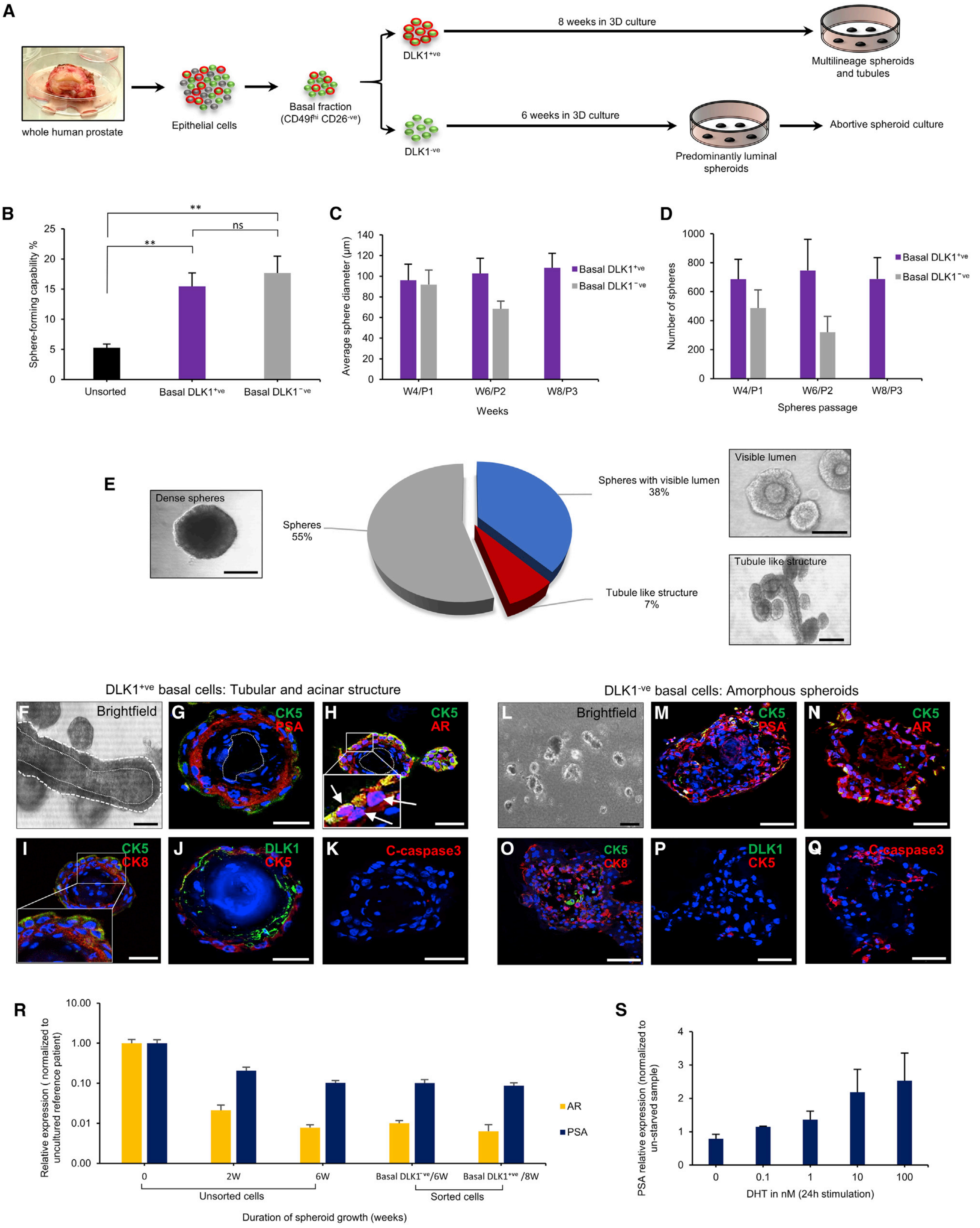

(legend on next page)

structures is thought to provide a measure of protection against the effects of deleterious mutations that confer a proliferative advantage on stem cells, inhibiting field change of the tissue. Mutant clones that derive from the transit-amplifying cell compartments may be “flushed” away through the constant turnover of tissue, while mutant clones derived from the resident stem cell compartment become restricted by the confines of the intestinal crypts. Whether the observed compartmentalization of the human prostate epithelium into discrete stem cell niches, segregated by tongues of urothelium analogous to the walls between adjacent intestinal crypts, provides the same degree of protection and, indeed, how the niche organization becomes disrupted or even subverted in the transition to neoplasia remains an interesting open question.

EXPERIMENTAL PROCEDURES

Patient Samples

Whole clinically benign prostates from cystectomy surgery for bladder cancer were collected from the Freeman Hospital, Newcastle upon Tyne with appropriate ethical review, informed consent, and regulatory approvals (Newcastle REC 2003/11 and Human Tissue Authority License 12534) (Table S2).

Whole-Prostate Sectioning and Marking of CCO-Deficient Epithelia

Whole clinically benign prostates were sectioned and every serial slide used for reconstruction of clonal patches was inspected by a clinical uropathologist to confirm normal histology (for further details, see Supplemental Experimental Procedures). Whole prostates were snap frozen and sequential CCO/succinate dehydrogenase (SDH) enzyme histochemistry was performed as previously described (Blackwood et al., 2011).

Formal 3D Reconstruction of Clonal Mapping of CCO-Deficient Clones

Sections were scanned into the Aperio virtual pathology system (Leica Microsystems, UK) and then imported into Reconstruct (v1.1.0.0; GNU General Public License) to allow formal 3D reconstruction to accurately describe proximal to distal polarity of the complex ductal systems. In total, 42 long-ranging CCO-deficient clonal patches originating in the proximal duct were identified, and 21 were fully mapped into detailed 3D reconstructions from 12 whole-prostate samples.

Laser Capture Microdissection (LCM) and RNA and mtDNA Sequencing

Areas of interest were captured using PALM MicroBeam laser micro-dissection microscope (Leica Microsystems, UK). Sequencing of the entire mitochondrial genome was undertaken using a two-stage amplification workflow, as previously described (Taylor et al., 2003). AROS Applied Biotechnology (Denmark) provided RNA sequencing using the Illumina HiSeq2500 platform. A Clontech Laboratories SMARTer (switching mechanism at 5′ end of RNA template, oligo dT primed) and PCR amplification of cDNA protocol was employed.

Whole-Prostate Epithelia Extraction and FACS

Whole human prostate samples were rapidly processed to release epithelial cells as previously described (Heer et al., 2006). Single cells were labeled with as described in Supplemental Experimental Procedures and sorted using the BD fluorescence-activated cell sorting (FACS) ARIA II cell sorter and DIVA software for analysis (BD Biosciences, UK).

3D Spheroid Culture

Sorted epithelial cells were transferred to a 1:1 mixture of Matrigel (BD, growth factor reduced, phenol-red-free) and PrEGM (Lonza) (Goldstein et al., 2011), which was modified to support differentiation by supplementation with 10 nM DHT (replenished every 48 hr).

Statistical Analyses

Data are expressed as means ± SEM. Statistical differences between experimental and control groups were determined by Student’s t test (unpaired, two tailed) unless otherwise specified. Excel was used for these statistical analyses. As further detailed in the Supplemental Experimental Procedures, least-squared fitting was used to deduce the rate of acquiring CCO-deficient mutations within prostatic sub-trees. The experimentally measured fractions of CCO-deficient epithelia per duct before and after bifurcation were used compute the probability of bifurcating into a large versus small duct or both and were compared to the analytical criteria derived in section 3.2 of the Supplemental Experimental Procedures.

SUPPLEMENTAL INFORMATION

Supplemental Information includes Supplemental Experimental Procedures, seven figures, one table, and two movies and can be found with this article online at http://dx.doi.org/10.1016/j.celrep.2017.07.061.

AUTHOR CONTRIBUTIONS

R.H., R.P., R.W.T., and D.M.T. conceived the project. M.M., E.H., S.J.B., L.W., D.S., L.G., A.E., N.W., S.C.W., C.N.R., R.W.T., D.M.T., B.D.S., and R.H. designed the experiments. M.M., L.W., L.G., and S.C.W. performed the experiments. M.M., E.H., S.J.B., L.W., A.E., B.D.S., and R.H. performed data analysis. All authors participated in manuscript writing.

ACKNOWLEDGMENTS

This work was supported by grants from The Royal College of Surgeons of England and a Cancer Research UK Clinician Scientist Fellowship (C10169/A12173). This work was also supported by the Wellcome Trust (grant 098357/Z/12/Z to B.D.S. and grant 110326/Z/15/Z to E.H.). E.H. is funded by a Junior Research Fellowship from Trinity College, Cambridge, a Sir Henry

Figure 6. DLK1 Basal Cells Demonstrate Stem Cell Function by Generating Prostate Spheroids Ex Vivo

(A) Schema outlining the 3D growth potential of selected cells from whole-prostate epithelium sorted into basal DLK1^{+ve} and DLK1^{-ve} populations. Sorted basal DLK1^{-ve} cells were viable for 6 weeks before becoming exhausted.

(B) Sphere-forming capacity of whole-prostate basal DLK1^{+ve} and DLK1^{-ve} sorted cells, error bars, SEM (**$p \leq 0.01$).

(C) Size of spheroid regeneration of basal cells through serial passage. Error bars, SEM.

(D) Number of spheres from basal cells through serial passage; P1, first-generation spheroids; P2, second-generation spheroids; P3, third-generation spheroids. Error bars, SEM.

(E) Histological patterning of DLK1^{+ve} basal cell-derived spheroids. Scale bars, 100 μm.

(F–Q) Bright-field and immunofluorescence for DLK1, basal (CK5), luminal (PSA, AR, CK8), and apoptosis (cleaved caspase-3) markers for DLK1^{+ve} basal cells at 8 weeks, scale bars, 50 μm (F–K) and DLK1^{-ve} basal cells at 6 weeks (L–Q). Scale bars, 50 μm.

(R) In the spheroids derived from unsorted cells and sorted fractions DLK1^{-ve} basal cells (6 weeks) and DLK1^{+ve} basal cells (8 weeks), AR mRNA was confirmed, albeit at low levels, and is associated with downstream readout of PSA expression. Error bars, SEM.

(S) To further validate the functionality of AR, dose-dependent induction of PSA was demonstrated in transiently androgen-starved spheroid culture. Error bars, SEM.

Wellcome Fellowship from the Wellcome Trust and acknowledges the Bettencourt-Schueller Young Researcher Prize for support. L.C.G., D.M.T., and R.W.T. are supported by the Wellcome Trust Centre Strategic Award (096919/Z/11/Z). R.W.T. is also supported by the Medical Research Council (MRC) Centre for Neuromuscular Diseases (G0601943), the Lily Foundation, and the UK National Health Service (NHS) Highly Specialised "Rare Mitochondrial Disorders of Adults and Children" Service. L.C.G. and D.M.T. receive support from the Newcastle University Centre for Ageing and Vitality funded by the Biotechnology and Biological Sciences Research Council (BBSRC), the Engineering and Physical Sciences Research Council (EPSRC), the Economic and Social Research Council (ESRC), and MRC as part of the cross-council Lifelong Health and Wellbeing Initiative.

Received: November 23, 2016
Revised: May 24, 2017
Accepted: July 21, 2017
Published: August 15, 2017

REFERENCES

Alexandrov, L.B., Jones, P.H., Wedge, D.C., Sale, J.E., Campbell, P.J., Nik-Zainal, S., and Stratton, M.R. (2015). Clock-like mutational processes in human somatic cells. Nat. Genet. *47*, 1402–1407.

Baker, A.M., Cereser, B., Melton, S., Fletcher, A.G., Rodriguez-Justo, M., Tadrous, P.J., Humphries, A., Elia, G., McDonald, S.A., Wright, N.A., et al. (2014). Quantification of crypt and stem cell evolution in the normal and neoplastic human colon. Cell Rep. *8*, 940–947.

Barker, N., van Es, J.H., Kuipers, J., Kujala, P., van den Born, M., Cozijnsen, M., Haegebarth, A., Korving, J., Begthel, H., Peters, P.J., and Clevers, H. (2007). Identification of stem cells in small intestine and colon by marker gene Lgr5. Nature *449*, 1003–1007.

Blackwood, J.K., Williamson, S.C., Greaves, L.C., Wilson, L., Rigas, A.C., Sandher, R., Pickard, R.S., Robson, C.N., Turnbull, D.M., Taylor, R.W., and Heer, R. (2011). In situ lineage tracking of human prostatic epithelial stem cell fate reveals a common clonal origin for basal and luminal cells. J. Pathol. *225*, 181–188.

Blanpain, C., and Simons, B.D. (2013). Unravelling stem cell dynamics by lineage tracing. Nat. Rev. Mol. Cell Biol. *14*, 489–502.

Boutros, P.C., Fraser, M., Harding, N.J., de Borja, R., Trudel, D., Lalonde, E., Meng, A., Hennings-Yeomans, P.H., McPherson, A., Sabelnykova, V.Y., et al. (2015). Spatial genomic heterogeneity within localized, multifocal prostate cancer. Nat. Genet. *47*, 736–745.

Boyd, L.K., Mao, X., Xue, L., Lin, D., Chaplin, T., Kudahetti, S.C., Stankiewicz, E., Yu, Y., Beltran, L., Shaw, G., et al. (2012). High-resolution genome-wide copy-number analysis suggests a monoclonal origin of multifocal prostate cancer. Genes Chromosomes Cancer *51*, 579–589.

Ceder, J.A., Jansson, L., Helczynski, L., and Abrahamsson, P.A. (2008). Delta-like 1 (Dlk-1), a novel marker of prostate basal and candidate epithelial stem cells, is downregulated by notch signalling in intermediate/transit amplifying cells of the human prostate. Eur. Urol. *54*, 1344–1353.

Choi, N., Zhang, B., Zhang, L., Ittmann, M., and Xin, L. (2012). Adult murine prostate basal and luminal cells are self-sustained lineages that can both serve as targets for prostate cancer initiation. Cancer Cell *21*, 253–265.

Chua, C.W., Shibata, M., Lei, M., Toivanen, R., Barlow, L.J., Bergren, S.K., Badani, K.K., McKiernan, J.M., Benson, M.C., Hibshoosh, H., and Shen, M.M. (2014). Single luminal epithelial progenitors can generate prostate organoids in culture. Nat. Cell Biol. *16*, 951–961.

Cooper, C.S., Eeles, R., Wedge, D.C., Van Loo, P., Gundem, G., Alexandrov, L.B., Kremeyer, B., Butler, A., Lynch, A.G., Camacho, N., et al.; ICGC Prostate Group (2015). Analysis of the genetic phylogeny of multifocal prostate cancer identifies multiple independent clonal expansions in neoplastic and morphologically normal prostate tissue. Nat. Genet. *47*, 367–372.

Cunha, G.R. (1972). Tissue interactions between epithelium and mesenchyme of urogenital and integumental origin. Anat. Rec. *172*, 529–541.

Donati, G., and Watt, F.M. (2015). Stem cell heterogeneity and plasticity in epithelia. Cell Stem Cell *16*, 465–476.

Drost, J., Karthaus, W.R., Gao, D., Driehuis, E., Sawyers, C.L., Chen, Y., and Clevers, H. (2016). Organoid culture systems for prostate epithelial and cancer tissue. Nat. Protoc. *11*, 347–358.

Gaisa, N.T., Graham, T.A., McDonald, S.A., Poulsom, R., Heidenreich, A., Jakse, G., Knuechel, R., and Wright, N.A. (2011). Clonal architecture of human prostatic epithelium in benign and malignant conditions. J. Pathol. *225*, 172–180.

Garraway, I.P., Sun, W., Tran, C.P., Perner, S., Zhang, B., Goldstein, A.S., Hahm, S.A., Haider, M., Head, C.S., Reiter, R.E., et al. (2010). Human prostate sphere-forming cells represent a subset of basal epithelial cells capable of glandular regeneration in vivo. Prostate *70*, 491–501.

Goldstein, A.S., Lawson, D.A., Cheng, D., Sun, W., Garraway, I.P., and Witte, O.N. (2008). Trop2 identifies a subpopulation of murine and human prostate basal cells with stem cell characteristics. Proc. Natl. Acad. Sci. USA *105*, 20882–20887.

Goldstein, A.S., Drake, J.M., Burnes, D.L., Finley, D.S., Zhang, H., Reiter, R.E., Huang, J., and Witte, O.N. (2011). Purification and direct transformation of epithelial progenitor cells from primary human prostate. Nat. Protoc. *6*, 656–667.

Goto, K., Salm, S.N., Coetzee, S., Xiong, X., Burger, P.E., Shapiro, E., Lepor, H., Moscatelli, D., and Wilson, E.L. (2006). Proximal prostatic stem cells are programmed to regenerate a proximal-distal ductal axis. Stem Cells *24*, 1859–1868.

Heer, R., Collins, A.T., Robson, C.N., Shenton, B.K., and Leung, H.Y. (2006). KGF suppresses alpha2beta1 integrin function and promotes differentiation of the transient amplifying population in human prostatic epithelium. J. Cell Sci. *119*, 1416–1424.

Heer, R., Robson, C.N., Shenton, B.K., and Leung, H.Y. (2007). The role of androgen in determining differentiation and regulation of androgen receptor expression in the human prostatic epithelium transient amplifying population. J. Cell. Physiol. *212*, 572–578.

Höfner, T., Eisen, C., Klein, C., Rigo-Watermeier, T., Goeppinger, S.M., Jauch, A., Schoell, B., Vogel, V., Noll, E., Weichert, W., et al. (2015). Defined conditions for the isolation and expansion of basal prostate progenitor cells of mouse and human origin. Stem Cell Reports *4*, 503–518.

Huang, J., and Witte, O.N. (2010). A seminal finding for prostate cancer? N. Engl. J. Med. *362*, 175–176.

Karthaus, W.R., Iaquinta, P.J., Drost, J., Gracanin, A., van Boxtel, R., Wongvipat, J., Dowling, C.M., Gao, D., Begthel, H., Sachs, N., et al. (2014). Identification of multipotent luminal progenitor cells in human prostate organoid cultures. Cell *159*, 163–175.

Kwon, O.J., Valdez, J.M., Zhang, L., Zhang, B., Wei, X., Su, Q., Ittmann, M.M., Creighton, C.J., and Xin, L. (2014). Increased Notch signalling inhibits anoikis and stimulates proliferation of prostate luminal epithelial cells. Nat. Commun. *5*, 4416.

Lamb, L.E., Knudsen, B.S., and Miranti, C.K. (2010). E-cadherin-mediated survival of androgen-receptor-expressing secretory prostate epithelial cells derived from a stratified in vitro differentiation model. J. Cell Sci. *123*, 266–276.

Lang, S.H., Stark, M., Collins, A., Paul, A.B., Stower, M.J., and Maitland, N.J. (2001). Experimental prostate epithelial morphogenesis in response to stroma and three-dimensional matrigel culture. Cell Growth Differ. *12*, 631–640.

Lawson, D.A., Xin, L., Lukacs, R.U., Cheng, D., and Witte, O.N. (2007). Isolation and functional characterization of murine prostate stem cells. Proc. Natl. Acad. Sci. USA *104*, 181–186.

Lawson, D.A., Zong, Y., Memarzadeh, S., Xin, L., Huang, J., and Witte, O.N. (2010). Basal epithelial stem cells are efficient targets for prostate cancer initiation. Proc. Natl. Acad. Sci. USA *107*, 2610–2615.

Leong, K.G., Wang, B.E., Johnson, L., and Gao, W.Q. (2008). Generation of a prostate from a single adult stem cell. Nature *456*, 804–808.

Liu, A.Y., Roudier, M.P., and True, L.D. (2004). Heterogeneity in primary and metastatic prostate cancer as defined by cell surface CD profile. Am. J. Pathol. *165*, 1543–1556.

Lopez-Garcia, C., Klein, A.M., Simons, B.D., and Winton, D.J. (2010). Intestinal stem cell replacement follows a pattern of neutral drift. Science *330*, 822–825.

Lu, T.L., Huang, Y.F., You, L.R., Chao, N.C., Su, F.Y., Chang, J.L., and Chen, C.M. (2013). Conditionally ablated Pten in prostate basal cells promotes basal-to-luminal differentiation and causes invasive prostate cancer in mice. Am. J. Pathol. *182*, 975–991.

Lukacs, R.U., Goldstein, A.S., Lawson, D.A., Cheng, D., and Witte, O.N. (2010). Isolation, cultivation and characterization of adult murine prostate stem cells. Nat. Protoc. *5*, 702–713.

McNeal, J.E. (1968). Regional morphology and pathology of the prostate. Am. J. Clin. Pathol. *49*, 347–357.

Ousset, M., Van Keymeulen, A., Bouvencourt, G., Sharma, N., Achouri, Y., Simons, B.D., and Blanpain, C. (2012). Multipotent and unipotent progenitors contribute to prostate postnatal development. Nat. Cell Biol. *14*, 1131–1138.

Payne, C.M., Holubec, H., Bernstein, C., Bernstein, H., Dvorak, K., Green, S.B., Wilson, M., Dall'Agnol, M., Dvorakova, B., Warneke, J., and Garewal, H. (2005). Crypt-restricted loss and decreased protein expression of cytochrome C oxidase subunit I as potential hypothesis-driven biomarkers of colon cancer risk. Cancer Epidemiol. Biomarkers Prev. *14*, 2066–2075.

Richardson, G.D., Robson, C.N., Lang, S.H., Neal, D.E., Maitland, N.J., and Collins, A.T. (2004). CD133, a novel marker for human prostatic epithelial stem cells. J. Cell Sci. *117*, 3539–3545.

Ritsma, L., Ellenbroek, S.I.J., Zomer, A., Snippert, H.J., de Sauvage, F.J., Simons, B.D., Clevers, H., and van Rheenen, J. (2014). Intestinal crypt homeostasis revealed at single-stem-cell level by in vivo live imaging. Nature *507*, 362–365.

Shen, M.M., and Abate-Shen, C. (2010). Molecular genetics of prostate cancer: new prospects for old challenges. Genes Dev. *24*, 1967–2000.

Snippert, H.J., van der Flier, L.G., Sato, T., van Es, J.H., van den Born, M., Kroon-Veenboer, C., Barker, N., Klein, A.M., van Rheenen, J., Simons, B.D., and Clevers, H. (2010). Intestinal crypt homeostasis results from neutral competition between symmetrically dividing Lgr5 stem cells. Cell *143*, 134–144.

Taylor, R.W., Barron, M.J., Borthwick, G.M., Gospel, A., Chinnery, P.F., Samuels, D.C., Taylor, G.A., Plusa, S.M., Needham, S.J., Greaves, L.C., et al. (2003). Mitochondrial DNA mutations in human colonic crypt stem cells. J. Clin. Invest. *112*, 1351–1360.

Tetteh, P.W., Farin, H.F., and Clevers, H. (2015). Plasticity within stem cell hierarchies in mammalian epithelia. Trends Cell Biol. *25*, 100–108.

Tsujimura, A., Koikawa, Y., Salm, S., Takao, T., Coetzee, S., Moscatelli, D., Shapiro, E., Lepor, H., Sun, T.T., and Wilson, E.L. (2002). Proximal location of mouse prostate epithelial stem cells: a model of prostatic homeostasis. J. Cell Biol. *157*, 1257–1265.

Valdez, J.M., Zhang, L., Su, Q., Dakhova, O., Zhang, Y., Shahi, P., Spencer, D.M., Creighton, C.J., Ittmann, M.M., and Xin, L. (2012). Notch and TGFβ form a reciprocal positive regulatory loop that suppresses murine prostate basal stem/progenitor cell activity. Cell Stem Cell *11*, 676–688.

Wang, X.D., Leow, C.C., Zha, J., Tang, Z., Modrusan, Z., Radtke, F., Aguet, M., de Sauvage, F.J., and Gao, W.Q. (2006). Notch signaling is required for normal prostatic epithelial cell proliferation and differentiation. Dev. Biol. *290*, 66–80.

Wang, X., Kruithof-de Julio, M., Economides, K.D., Walker, D., Yu, H., Halili, M.V., Hu, Y.P., Price, S.M., Abate-Shen, C., and Shen, M.M. (2009). A luminal epithelial stem cell that is a cell of origin for prostate cancer. Nature *461*, 495–500.

Wang, Z.A., Mitrofanova, A., Bergren, S.K., Abate-Shen, C., Cardiff, R.D., Califano, A., and Shen, M.M. (2013). Lineage analysis of basal epithelial cells reveals their unexpected plasticity and supports a cell-of-origin model for prostate cancer heterogeneity. Nat. Cell Biol. *15*, 274–283.

Winton, D.J., and Ponder, B.A. (1990). Stem-cell organization in mouse small intestine. Proc. Biol. Sci. *241*, 13–18.

Xin, L., Ide, H., Kim, Y., Dubey, P., and Witte, O.N. (2003). In vivo regeneration of murine prostate from dissociated cell populations of postnatal epithelia and urogenital sinus mesenchyme. Proc. Natl. Acad. Sci. USA *100* (*Suppl 1*), 11896–11903.

Xin, L., Lukacs, R.U., Lawson, D.A., Cheng, D., and Witte, O.N. (2007). Self-renewal and multilineage differentiation in vitro from murine prostate stem cells. Stem Cells *25*, 2760–2769.

Zhang, D., Park, D., Zhong, Y., Lu, Y., Rycaj, K., Gong, S., Chen, X., Liu, X., Chao, H.P., Whitney, P., et al. (2016). Stem cell and neurogenic gene-expression profiles link prostate basal cells to aggressive prostate cancer. Nat. Commun. *7*, 10798.

Cell Reports, Volume *20*

Supplemental Information

Multipotent Basal Stem Cells, Maintained in Localized Proximal Niches, Support Directed Long-Ranging Epithelial Flows in Human Prostates

Mohammad Moad, Edouard Hannezo, Simon J. Buczacki, Laura Wilson, Amira El-Sherif, David Sims, Robert Pickard, Nicholas A. Wright, Stuart C. Williamson, Doug M. Turnbull, Robert W. Taylor, Laura Greaves, Craig N. Robson, Benjamin D. Simons, and Rakesh Heer

Supplemental Figures and Experimental Procedures

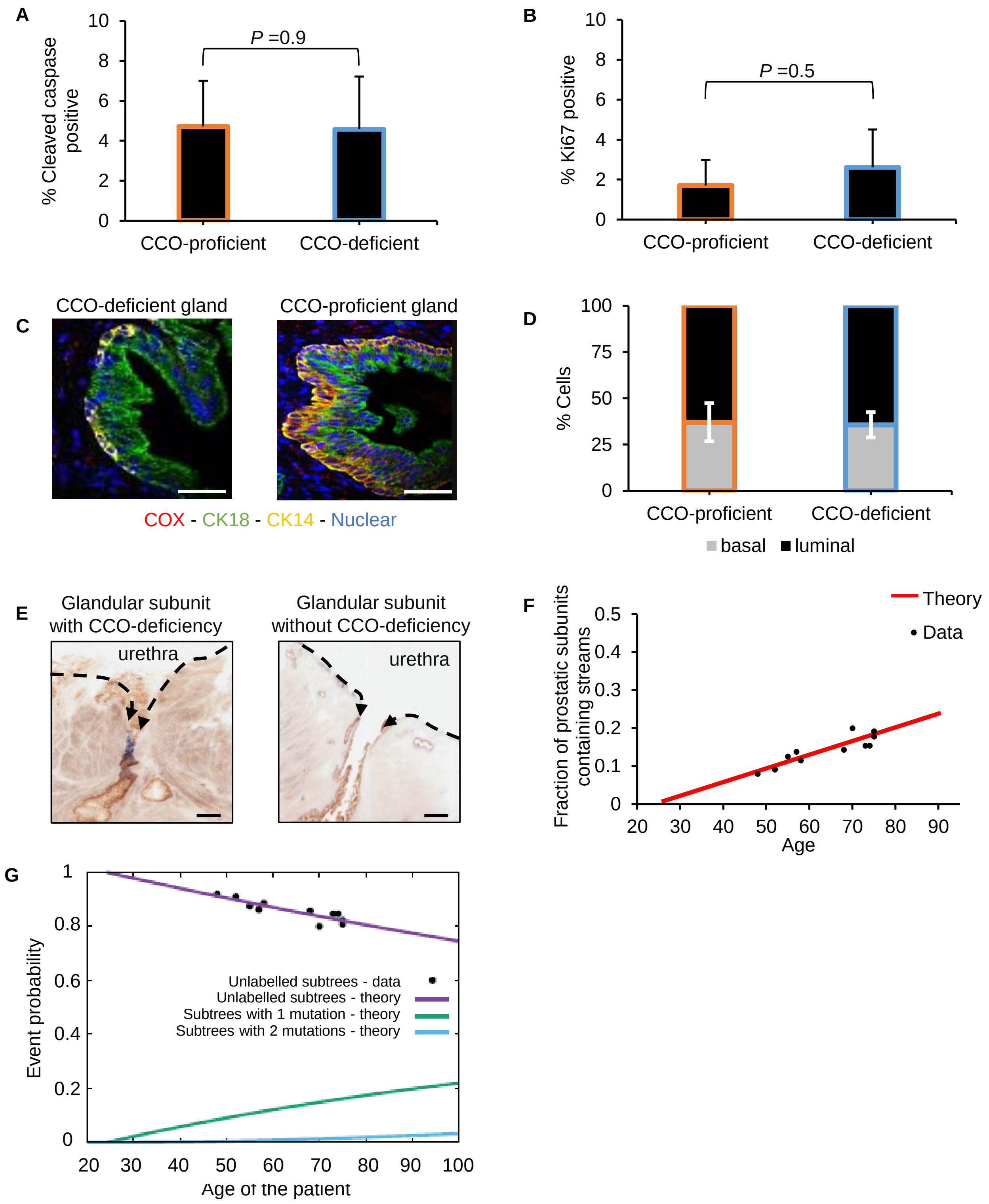

Figure S1: mtDNA mutation serves as a neutral marker in prostate epithelium. Related to Figures 1 and 2. (A) The apoptosis rates (cleaved caspase-3) and **(B)** the proliferation rates (Ki67 positive cells) showed no significant difference in CCO-proficient and deficient epithelial cells (n = 3 prostates; 12 random regions; ca. 10,000 cells for each measure), confirming that cells within CCO-deficient clones behave identically to cells from the rest of the CCO-proficient epithelium. **(C)** Immunofluorescence staining of anti-complex IV subunit I (COX-I) allow identification of CCO-deficient areas and co-staining with basal and luminal specific markers, CK5 and CK8 respectively. Scale bars, 50 µm. **(D)** Basal and luminal cell ratio were statistically equivalent in both CCO-deficient and CCO-proficient regions (n = 3 prostates; 12 regions of interest; ca. 13,500 cells). **(E)** Examples of CCO-proficiency and CCO-deficiency in the most-proximal trunk of the glandular subunit, draining into prostatic urethra (arrows marking duct openings). Scale bars, 100 µm. **(F)** The fraction of most-proximal trunks containing CCO-deficient streams was low, consistent with mtDNA mutations being acquired at extremely low rates. An increasing trend with age was observed, fitting a Poisson process (red curve) describing the rate of mutation being constant throughout the lifetime of the patient. This implies that a chance of a double occurrence of CCO-deficiency within the same gland subunit can be neglected and that a long sustained clonal stream is derived from a single clonal event, i.e. from a single stem cell. **(G)** Comparative probabilities of one and two separate clonal inductions (green and blue curves respectively) are shown, each generating long-ranging clonal marks and were superposed to the theoretical and experimental probability of unlabeled subunits with age (purple curve and black dots respectively).

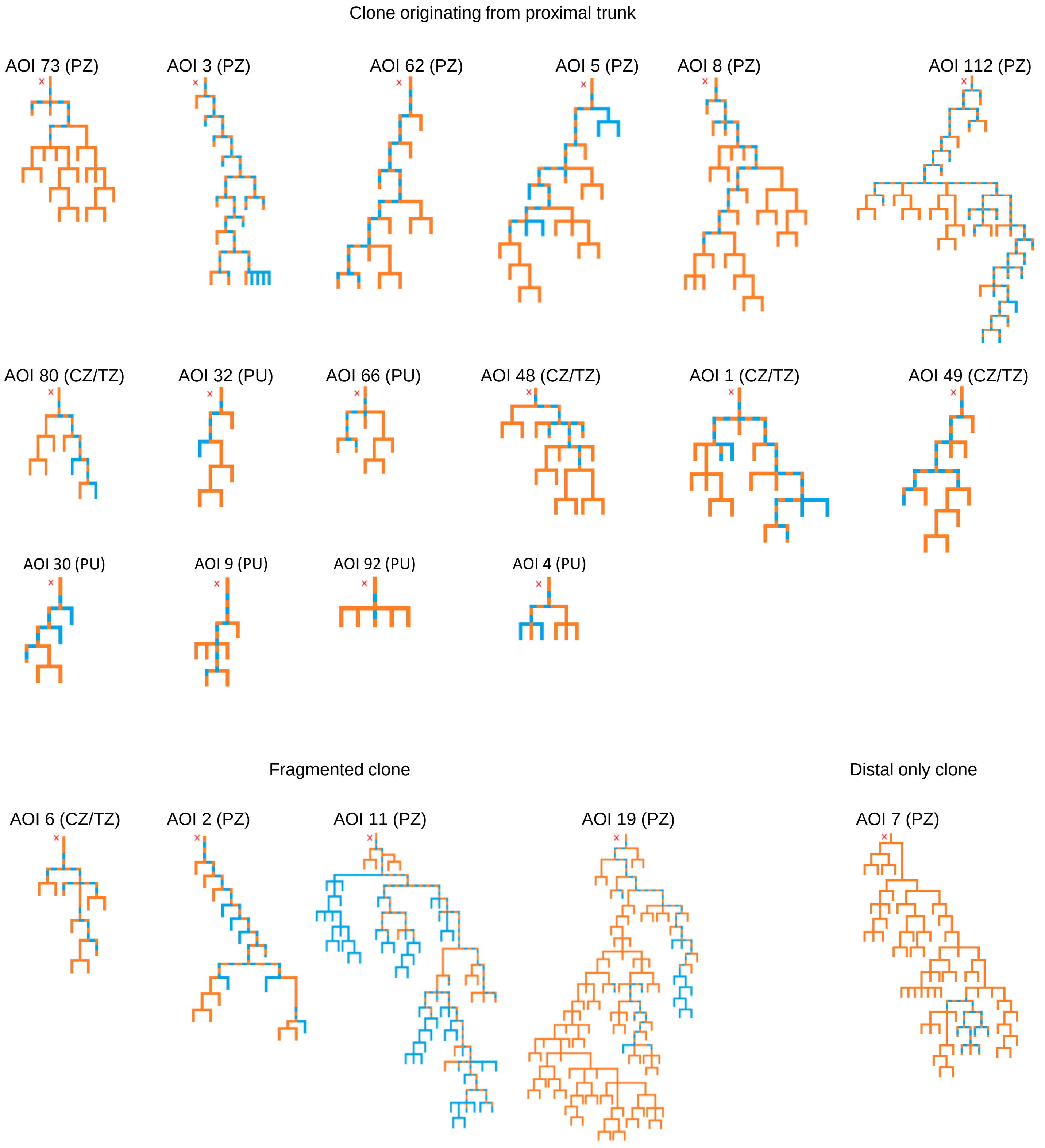

Figure S2: Summary topography of all 3-D reconstructions. **Related to Figures 1, 2, and 3.** Data is summerised as described in Supplementary Movie 1, showing 3D reconstructions from 12 patients, including annotations of anatomical zones. Clonal patches were observed in subunits arising along the entire length of the urethra. The mapped subunits also have varying reach through the stroma to the periphery of the prostate. These spatial distributions allows annotations of peri-urethral (PU), central (CZ)/transitional (TZ) and peripheral (PZ) zonal anatomy of the prostate (McNeal, 1968). The full extent of the marked clone is mapped and adjacent areas of unmarked tree are only partially resolved.

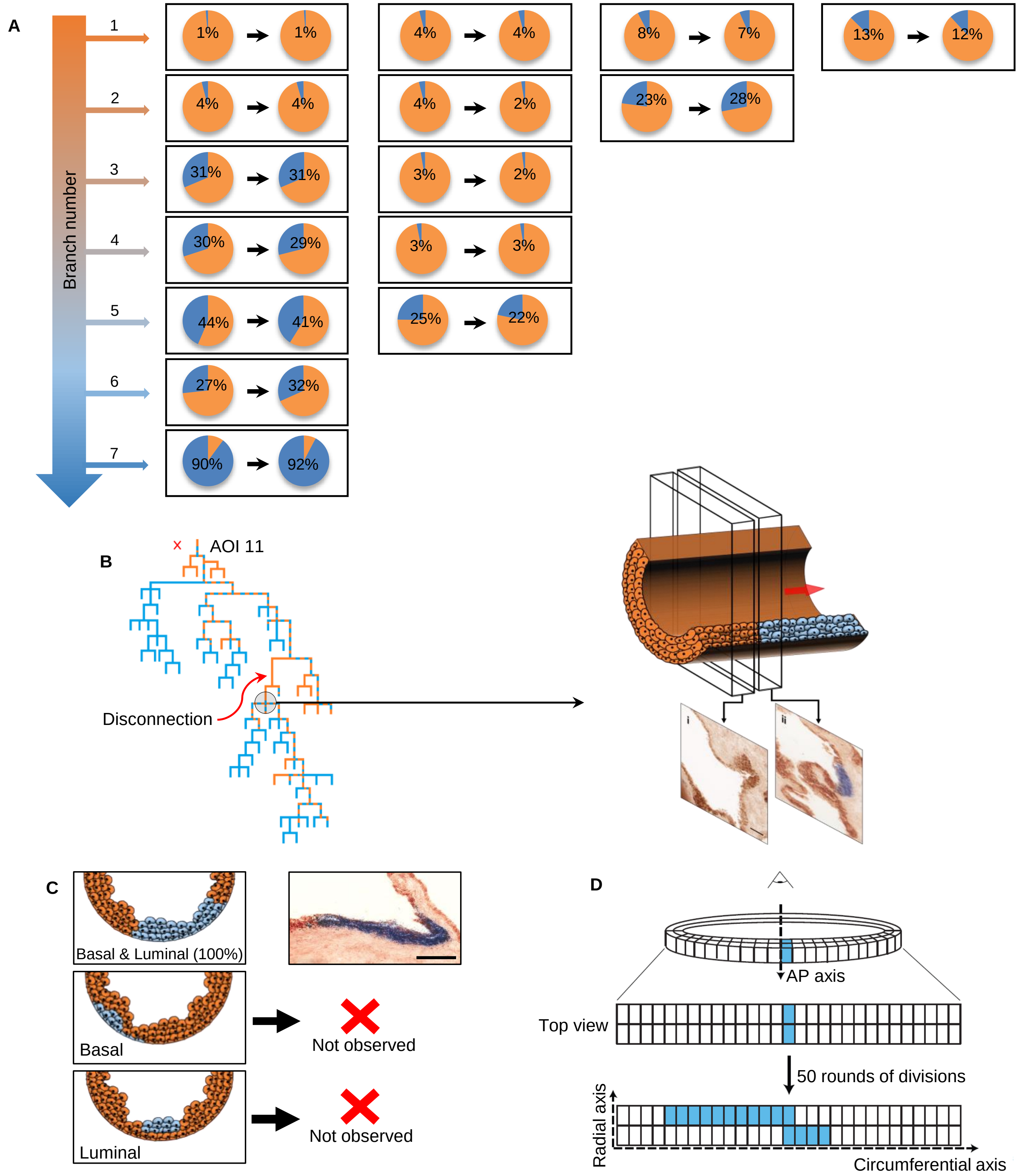

Figure S3: Proportional distribution of the clonal patches and the microanatomy at the start of distant clones. Related to Figures 3 and 4. (A) The ductal fraction occupied by clonal streams in the parent ducts is preserved in the daughter ducts at all levels of branch generation measured (n = 15 clones; n = 3 prostates). These findings also illustrate the neutrality of the CCO-deficient clonal mark across branch points and confirm a pattern of directional proximodistal flow throughout the gland. **(B)** Tree-representation of a disconnected clone and an associated schema demonstrating the histological pattern at the onset of a clonal stream – The distal stream contains both basal and luminal cells from the first slide where it is present (i and ii represent consecutive slides from serial z-stacks). **(C)** In contrast to the start of proximal clones, the distal disconnected clonal patches invariably demonstrate synchronous basal and luminal cell overlay, with no examples of luminal only or basal only clones. **(D)** A sketch of a numerical simulation of independently maintained luminal and basal compartments based on loss-replacement dynamics. As a function of time, division events accumulate and luminal and basal clone sizes drift independently, in stark contrast to the synchrony actually observed in the clonal patches. This is consistent with luminal cells being constantly lost and replenished through the asymmetrical division of local underlying basal progenitors. Scale bars, 100 µm

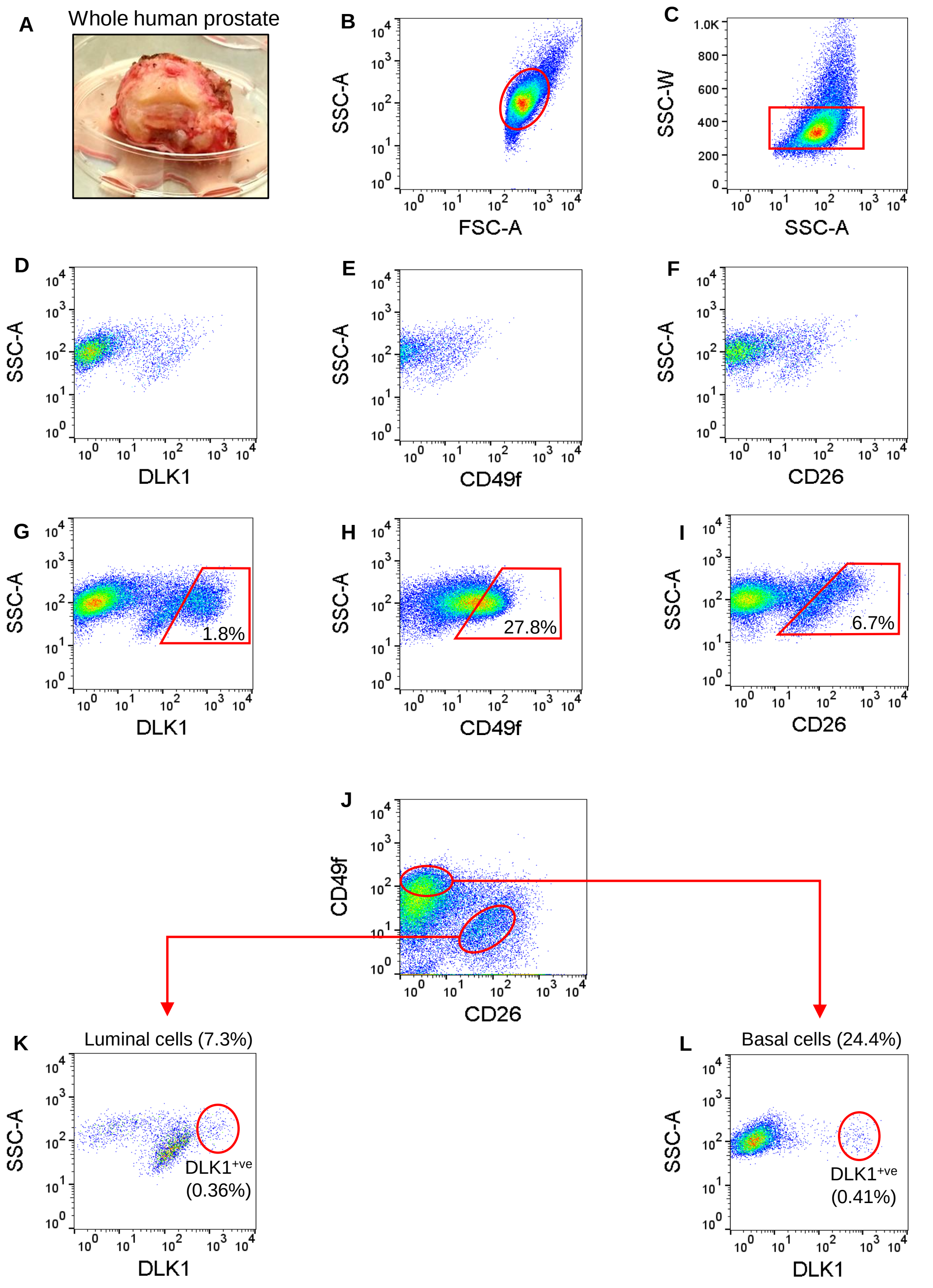

Figure S4: FACS selection strategy. Related to Figure 6. (A) Whole prostates were processed to release EpCAM^{+ve} epithelial cells as described in the Supplementary Methods. **(B)** Size gating was applied to enrich for whole cells. **(C)** Doublet discrimination was undertaken to avoid false positive measures. **(D-F)** Isotope antibody controls were used to mark boundaries for positive measures for DLK1, CD49f (basal) and CD26 (luminal prostate epithelial marker). **(G-I)** Illustration of positive gates for capture illustrated for DLK1, CD49f and CD26; including fractions of cells positive. **(J)** Dual staining for CD49f and CD26 to discriminate basal and luminal cells shown. **(K-L)** Gating of DLK1 shown in basal and luminal cells.

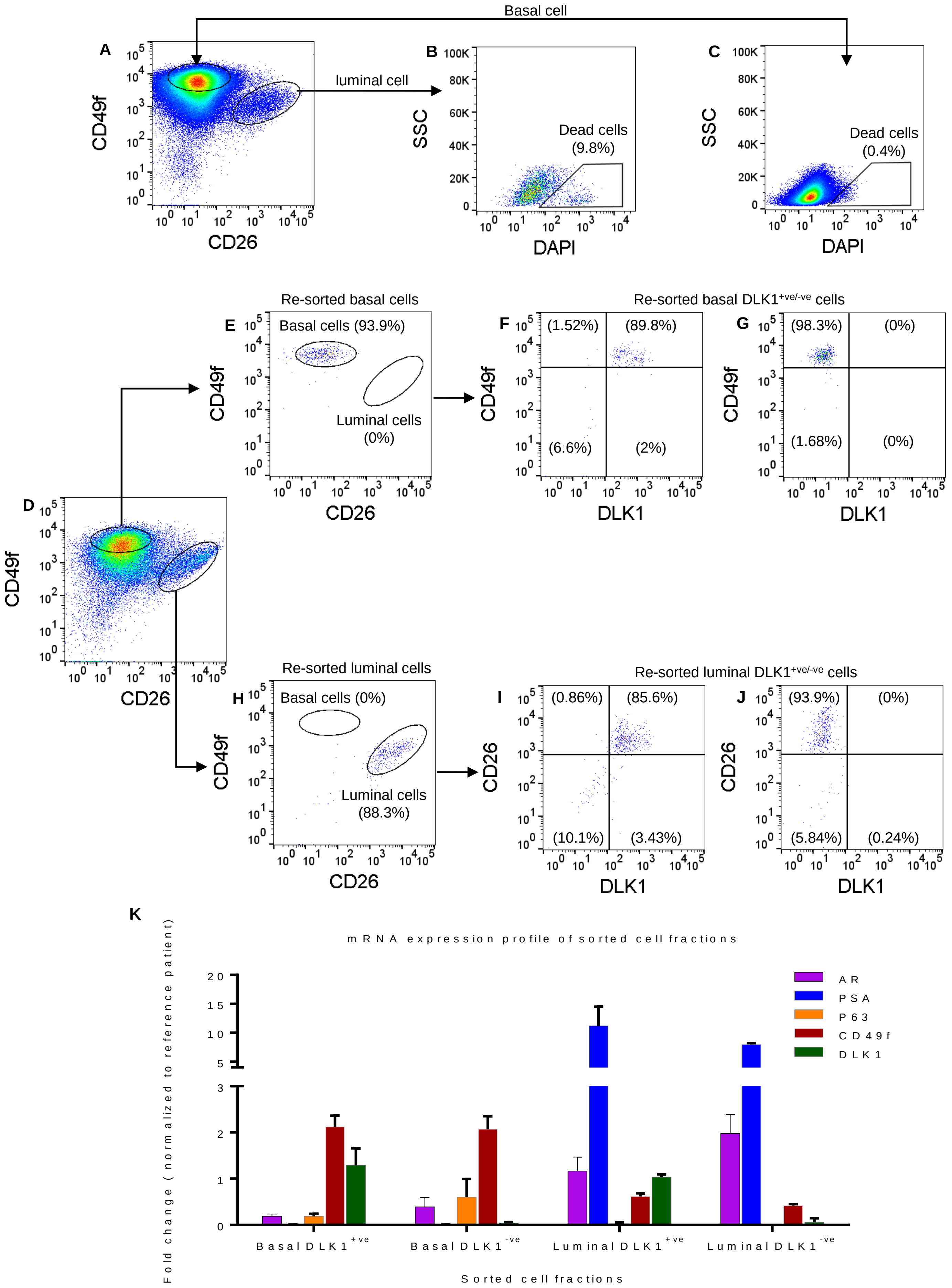

Figure S5: Purity of sorted cells. Related to Figure 6. (A-C) Freshly extracted whole prostate basal and luminal epithelial cells have low rates of dead cells determined by DAPI uptake live/dead discrimination. **(D-J)** Flow re-sorting based on DLK1 expression was performed and confirmed minimal cross-contaminations of basal DLK1^{+ve} and DLK1^{-ve} selections. **(K)** PCRs show that DLK1 mRNA expressions correlated with DLK1 sorted cells and that basal sorted cells enrich for basal markers (CD49hi and p63) and luminal sorted cells enrich for luminal markers (AR and PSA).

A

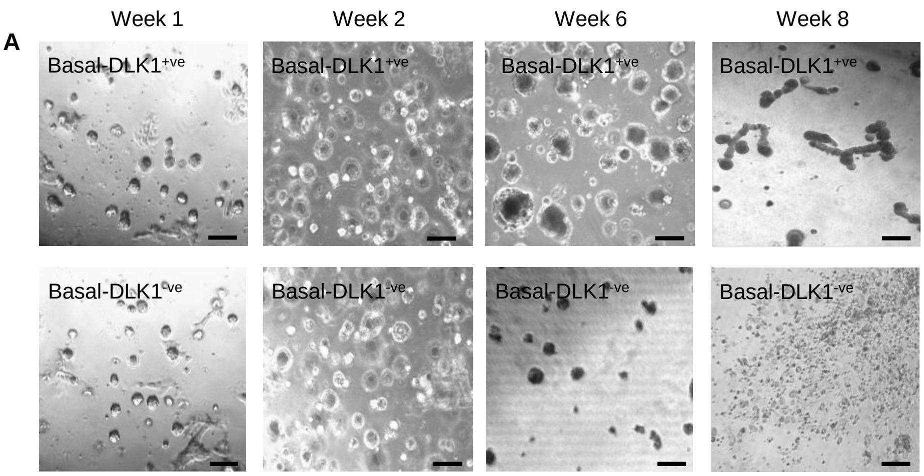

B

Immunofluorescent phenotype of basal DLK1^{+ve} derived spheroids at week 8			
	CK5 cells	CK8 cells	CK5/8 cells
% of spheres	100%	100%	100%
% of cells*	24.4%	42.2%	27.6%

*<5% of cells showed absent expression of CK5, CK8 or CK5/8

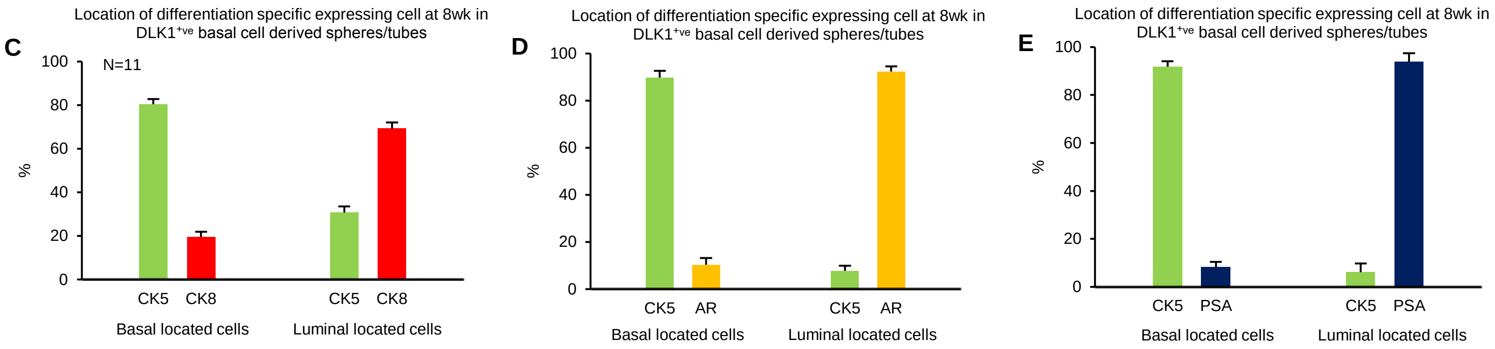

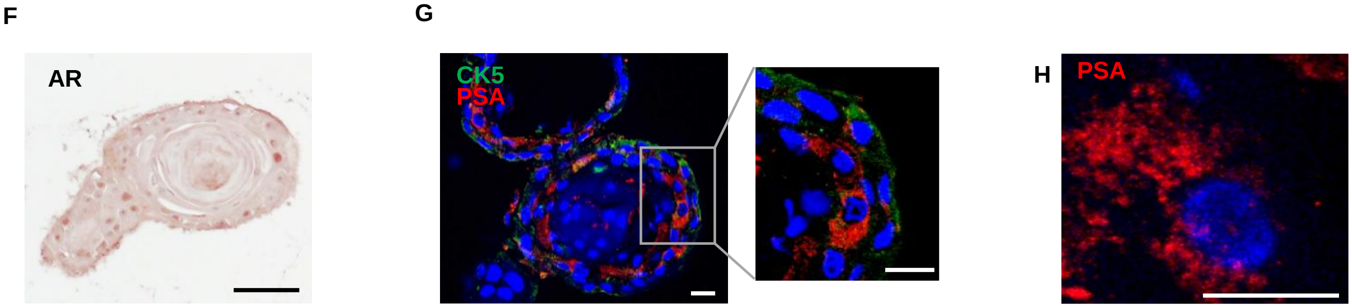

Figure S6: Quantification of differentiation in sphere cultures. Related to Figure 6. (A) Brightfield views of DLK1^{+ve} and DLK1^{-ve} sorted basal cells reveal similar 3-D morphology up to 6 weeks, but thereafter a divergent phenotype emerged. DLK1^{-ve} cell growth exhausted and marked apoptosis occurred by 8 weeks, whereas sustained growth in the DLK1^{+ve} cells was associated with ductal growth and pronounced lumen formation. Scale bars, 100 µm. **(B)** At week 8 all the basal DLK1^{+ve} derived spheroids contain cells that are positive for basal (CK5) marker, luminal (CK8) marker, or CK5/CK8 (double positive). Also, within each spheroid, proportion of CK5 positive, CK8 positive, and CK5/CK8 double positive cells are shown. **(C, D, E)** The spatial localisations of CK5 vs. CK8, CK5 vs. AR, and CK5 vs. PSA within hollow DLK1+ve basal cell derived spheres/tubes show appropriate histological distributions of differentiation marks at week 8. **(F)** IHC staining of basal DLK1^{+ve} derived prostate spheroids at 8 weeks showing AR nuclear localisation. Scale bars, 100 µm. **(G, H)** Immunofluorescence is shown for basal (CK5) marker and luminal (PSA) marker for basal DLK1^{+ve} derived prostate spheroids at week 8. Including higher magnification showing classical vesicular patterning for PSA. Scale bars, 20 µm.

Table S2: Clinical characteristics for the patient samples in this study.

Patient ID	Patient age (yrs)	Prostate volume	Patient diagnosis	Treatment
12490	48	64.4 cm^3	Neuropathic bladder	Cystoprostatectomy with ileal conduit diversion
12526	52	42.0 cm^3	TCC of the bladder- Stage T1 grade G3	Cystoprostatectomy with ileal conduit diversion
12636	68	107.9 cm^3	TCC of the bladder- Stage T1 grade G2 HG	Cystoprostatectomy with ileal conduit diversion
12367	74	64.8 cm^3	TCC with mixed differentiation cancer of the bladder – Stage T1 HG	Cystoprostatectomy with ileal conduit diversion
12524	74	180.0 cm^3	TCC of the bladder- Stage T2 grade G3	Cystoprostatectomy with ileal conduit diversion
12368	75	68.3 cm^3	TCC of the bladder –Stage T1 grade G3	Cystoprostatectomy with ileal conduit diversion
12505	55	n/a	Refractory overactive bladder symptoms	Cystoprostatectomy with ileal conduit diversion
12942	64	n/a	pT1 + CIS, refactory to BCG	Cystoprostatectomy with ileal conduit diversion
12990	57	54.1 cm^3	CIS of the bladder	Cystoprostatectomy with ileal conduit diversion
13175	58	109.8 cm^3	TCC of the bladder –Stage T2 grade G3	Cystoprostatectomy with ileal conduit diversion
13214	73	n/a	TCC of the bladder –Stage T2 grade G3	Cystoprostatectomy with ileal conduit diversion
13683	70	58.0 cm^3	TCC of the bladder –Stage T1 grade G3	Cystoprostatectomy with ileal conduit diversion
13826	54	50.8 cm^3	TCC of the bladder - Stage T1 grade G3	Cystoprostatectomy + orthotopic neobladder
13874	49	n/a	Neurogenic bladder dysfunction	Cystoprostatectomy with ileal conduit diversion
13888	52	55.8 cm^3	TCC of the bladder - stage pT2b High grade	Cystoprostatectomy with ileal conduit diversion and right nephrectomy for non-functioning kidney
13905	75	n/a	TCC of the bladder - Stage pT1 + CIS; refractory to BCG	Cystoprostatectomy with ileal conduit diversion
13914	71	25.7 cm^3	TCC of the bladder - pT3a	Cystoprostatectomy with ileal conduit diversion
13973	74	37.8 cm^3	TCC of the bladder - pT1 High grade	Cystoprostatectomy with ileal conduit diversion
14026	84	174.5 cm^3	TCC of the bladder - pT2 High grade	Cystoprostatectomy with ileal conduit diversion
14136	57	109.1 cm^3	TCC of the bladder - pT4 (into rectum)	Cystoprostatectomy with ileal conduit diversion
14146	68	126.9 cm^3	TCC of the bladder - pT1 sacromatoid TCC differentiation	Cystoprostatectomy with ileal conduit diversion
14147	62	53.0 cm^3	SCC of the bladder - pT2 SCC bladder	Cystoprostatectomy with ileal conduit diversion
14231	56	75.0 cm^3	TCC of the bladder - stage T4 (rectal invasion) N1	Cystoprostatectomy with ileal conduit diversion
14249	54	35.5 cm^3	Overactive bladder with marked lower urinary tract symptoms	Cystoprostatectomy with ileal conduit diversion
14250	73	88.8 cm^3	Focal CIS of the bladder - refractory to BCG	Cystoprostatectomy with ileal conduit diversion

Abbreviations: HG-High Grade; **TCC**-Transitional Cell Carcinoma; **SCC**-Squamous Cell Carcinoma; **CIS**-Carcinoma in situ

Supplementary experimental procedures

Patient samples

Whole clinically benign prostates from cystectomy surgery for bladder cancer were collected from the Freeman Hospital, Newcastle upon Tyne with appropriate ethical review, informed consent and regulatory approvals (Newcastle REC 2003/11 and Human Tissue Authority Licence 12534). Whole prostates were collected from 25 patients aged between the ages of 48 and 75 years. Patients underwent routine clinical assessment with digital rectal examination and PSA biomarker screening of prostate cancer in haematuria clinics.

Pathological assessment

In cases with clinical suspicion of prostate cancer at the time of cystectomy (overt local extension or palpable prostate node), the whole prostate was sent for routine clinical pathology and was not included in this study. In those prostates included in this paper, the histology was formally assessed by a clinical uropathologist (AE) and confirmed normal aging prostate biology, the absence of prostate cancer and gross prostatitis. In brief, a detailed workflow comprised of formal histological assessment of every slide that contained clonal marks as part of the extensive gland reconstruction and confirmed no cancer or premalignant changes such HG PIN, ASAP or overt prostatitis. To support this histological assessment, an immunostaing cocktail of AMACR and CK5/14 was undertaken. Although, focal prostatitis is commonly found in ageing prostates and occult foci of cancer are reported in up to 25% of prostates from cystoprostatectomy, the absence of these pathologies in the clonal patches was not surprising as it reflected: (1) PSA biochemical screening for cancer, (2) cases of gross incidental palpable masses indicative for cancer were sent for formal clinical histology as part of the cystectomy specimen and (3) of the remaining screened prostates, the infrequent nature of the clonal mark meant that the lack of coincidence with a foci of cancer or overt inflammation was expected.

Cytochrome c oxidase (CCO) labelling

Infrequent stochastic loss of CCO activity (CCO-deficient) is attributed to underlying somatic mitochondrial DNA (mtDNA) mutations that accumulate in long-lived progenitor/stem cells as part of normal ageing. Essentially, age-related mtDNA mutations clonally expand through stochastic intracellular drift(Coller *et al.*, 2001; Elson *et al.*, 2001; Greaves *et al.*, 2014). Multiple mtDNA mutations arise, with individual mutations expanding and regressing within the total pool of heteroplasmic mtDNA. However, as the mutational burden for a specific mutation rises, it becomes decreasingly likely to regress, and the power of drift increases. When a heteroplasmic threshold of 70-80% is reached, this results in a loss of normal CCO function. These mtDNA mutations are transmitted to daughter cells and allow clonal mapping of stem cell fate *in situ.* Two-colour enzyme histochemistry can simultaneously detect activity of the mtDNA-encoded cytochrome *c* oxidase and nuclear DNA-encoded succinate dehydrogenase (SDH) with cells lacking in cytochrome *c* oxidase activity appearing blue (CCO-deficient) and cells with active cytochrome *c* oxidase appear brown (CCO-proficient). We have previously used this method to trace stem cell fate in epithelial tissues from colon, stomach, liver and the prostate(Taylor *et al.*, 2003; Greaves *et al.*, 2006; Fellous *et al.*, 2009; Blackwood *et al.*, 2011).

Prostates were incised into the urethra, approximately at the lateral margins of the anterior fibromuscular stroma. This generated a gland cross-section compatible with a standard slide and illustrated the vast majority of the glandular sub-trees arising from the posteriolateral aspect of the urethra into the peri-urethral, central, transitional and as far as the peripheral zone can be captured if sectioned along the longitudinal axis of the urethra. Prostate tissues were rapidly snap frozen in isopentane cooled with liquid nitrogen and sectioned into 20 microns slices. CCO activity was measured by incubating sections with CCO solution (cytochrome C and DAB) at 37°C for 90mins. The sections were then washed and incubated with SDH solution (sodium succinate, NitroBlue tetrazolium, phenazine methosulphate and sodium azide) at 37°C for 30mins, as previously described(Blackwood *et al.*, 2011). Sections were then dehydrated and mounted using DPX for scanning and visualisation on the Aperio (Leica Microsystems, UK) virtual pathology system.

Mitochondrial DNA sequencing

Areas of interest were captured directly from glass slides on a PALM MicroBeam laser micro-dissection microscope (Leica Microsystems, UK). Whole mitochondrial DNA sequencing was undertaken using a two-stage amplification workflow. Long-range mtDNA amplification was followed by PCR using 36 pairs of M13-tagged oligodeoxynucleotide primers, as previously described (Taylor *et al.*, 2003). Amplified PCR products were sequenced using BigDye® Terminator v3.1 chemistries (Life Technologies, Paisley, UK) on an ABI3130 Genetic Analyser. The published Cambridge reference dataset (GenBank Accession number NC_012920.1) was used for sequencing comparison to confirm that mtDNA variants identified were somatic mutations and responsible for CCO-deficiency, by comparing against mtDNA sequences in CCO-proficient areas.

Automated histology scanning archive and 3D Reconstruction

Stained sections were scanned into the Aperio virtual pathology system (Leica Microsystems, UK). Images were captured at various magnifications dependent on the size of the gland, with a maximum magnification of 20x. Serial z-stack images were imported into the 3D reconstruction programme Reconstruct (v1.1.0.0; GNU General Public License) to describe proximal to distal polarity of the complex ductal systems. Scanned images were initially manually aligned and the reconstruction programme generated 3D models of the branching structures of entire glandular subunits that were presented as z-traces. Videos were created to inform the spatial orientation of these complex and convoluted gland structures and to aid subsequent topographical deconstructions to illustrate in more basic and workable terms the key organisational relationships of the branching structures and clonal maps in the entire prostate glandular subunit. CCO-deficient clonal mapping was tracked from the proximal urethral breach points to the end of individual glandular subunit structures in the periphery of the prostate, which were then spatial rendered, as described above, into topographical maps to aid analysis according to branch level. In total, 21 CCO-deficient clonal patches were identified and tracked through serially stained sections from 12 whole prostate samples.

Immunofluorescence

For *in situ* tissue staining, serially cut prostate frozen sections were alternatively stained for CCO/SDH. Areas of CCO deficiency (blue) were identified and serial sections underwent 3-D reconstruction to characterise areas of interest (AOI). The immediately adjacent unstained slides were assayed using dual immunofluorescence. For organoid cultures, serial frozen sections (7 μm) were prepared for multiplex immunofluorescence. For formalin-fixed paraffin-embedded prostates, immunofluorescence was undertaken using an anti-complex IV subunit I (CCO-1) antibody (1:200; Mito-sciences, USA), TSA goat anti-mouse IgG kit and Alexa Fluor546 tyramide (Invitrogen, UK). Luminal cell co-staining was performed with an anti-cytokeratin 18 FITC-conjugated antibody (1:100; DC-10, Abcam, Cambridge, UK) and for basal cells we used anti-cytokeratin 14 antibody (1:100; Novocastra, UK) detected with Biotin-SP-AffiniPure goat anti-mouse IgG3 antibody (1:100; Jackson Immunoresearch, USA) and streptavidin Alexa Fluor 647 conjugate (1:100; Invitrogen, UK). For frozen tissue, sections were fixed with 4% paraformaldehyde, permeabilised using 0.1% triton and blocked in 4% BSA before incubation with primary antibody antibodies. The antibodies used were DLK1 (1:50; C-19; sc-8624; Santa Cruz Biotechnology, USA), Notch1 (1:100; C-20; sc-6014-R; Santa Cruz Biotechnology, USA), PSA (1:100; A0562; Dako, USA), AR (1:50; N-20; sc-816; Santa Cruz Biotechnology, USA), the basal marker CD49f-APC (1:50; GoH3; 313616; BioLegend, UK) and CK5 (1:50; RCK103; sc-32721; Santa Cruz Biotechnology, USA), CK5/14 (1:100; 16.4; ab16570; Abcam, UK) and the luminal marker CK8/18 (1:100; 5D3; ab17139; Abcam, UK). Secondary antibody Alexa Fluor 488 (1:100; ab150129; Abcam, UK), Alexa Fluor-568 (1:100; A-10042; Invirtogen, USA), Alexa Fluor-546 (1:100; A-11030; Life Technologies, USA) was used to detect the bound unconjugated primary antibody. Sections were washed and mounted using Vectashield with DAPI mountant (Vector Laboratories, Peterborough, UK) before being visualised on the confocal.

Immunohistochemistry

Immunohistochemistry using Ki67 (1:100; M7240, Dako, Denmark), 34BE12 (790-4373; Ventana, USA), PSA (760-2506; Ventana, USA) and Cleaved Caspase 3 (1:200, Antibody 9661, New England Biolabs, US) was performed on FFPE sections (4 μm) of a selected region of whole prostate mega blocks that were initially deparaffinised and hydrated. Ki67 and c-caspase-3 immunohistochemistry were performed on FFPE and correlated with serial section COX (Complex IV subunit 1) staining, to identify CCO deficiency

Microwave Antigen retrieval preformed with citrate buffer pH6 to unmask surface antigens. Endogenous peroxidase activity was removed by blocking with 3% H_2O_2. Sections were then blocked in horse serum and incubated in primary antibody overnight 4°C. Sections were washed and incubated with anti-rabbit secondary (vector ImmPRESS HRP Anti-Rabbit IgG (Peroxidase) Polymer Detection Kit (Vector Laboratories, UK). Antibody detected with DAB solution (ImmPACT DAB Substrate Kit, Vector Laboratories, UK) and counterstained with haematoxylin, dehydrated and mounted using DPX. Slides were then visualised on the

Aperio and scored using automated analysis. Using IHC on 4 µm sequential sections we were able to define the basal layer with 34BE12 staining and luminal layer with PSA staining. Using the Aperio software, we were able to overlay 34BE12 and PSA masks to accurately count basal and luminal Ki67 and caspase-3 staining.

Automated histology scoring system

Aperio automated analysis was performed on CCO/SDH slides to generate the percentage of CCO deficiency (blue) present in a gland. Aperio *Genie* recognition software was used to discriminate between epithelia and stroma and verified by uropathologist (AE). Algorithm used for CCO/SDH scoring was based on the positive pixel count. Automated capture of pixel area measurement of CCO-deficient staining was undertaken using Aperio *Genie*, and where appropriate, extrapolated to cell volume calculations based on solid section thickness of serial slides at 20µm. Similarly, Aperio automated analysis performed on cleaved-Caspase 3 and Ki67 staining for measures of voxel based intensity and thresholds were corroborated by an expert uropathologist (AE). Positive staining was detected by Aperio *cytoplasmic v2 algorithm*, detecting both nuclear and cytoplasmic staining and presented the data as the percentage of cells that are negative, weak, moderate or strong positive in the nucleus and the cytoplasm. Automated Aperio *Genie* software used to distinguish between prostatic epithelia and urothelium, again verified by an expert uropathologist (AE). To define crude patterns in staining along the proximal to distal axis whole prostates were divided into 10 equal regions in a radial fashion from the urethra to the peripheral prostate (see figure to the right).

Primary prostate tissue processing and FACS for luminal and DLK$^{+ve/-ve}$ basal cells

Whole human prostate samples (from cystectomy surgery) were immediately transferred to culture media (RPMI-1640 Media supplemented with 10% Fetal Bovine Sera, Sigma-Aldrich, UK), rapidly transferred to the laboratory. The tissue was finely cut into small chunks and incubated for 8-12hrs at 37°C in collagenase/dispase digestion solution (collagenase (17018-029, type I, Thermo Fisher Scientific, USA) and dispase (17105-041, Thermo Fisher Scientific, USA) in DMEM with 10% FBS, 1% L-glutamine (2mM) (Sigma) and 1% Penicillin-Streptomycin (Sigma) to a final concentration of 1 mg/ml for each enzyme) with continuous rocking action to separate epithelia from stroma. Epithelia were then washed with PBS, and incubated in 1 ml of TrypLE (12605-010; Life Technologies, USA) for 15 min at 37 °C with occasional shaking to release single cells followed by passing several times through a 21-gauge syringe. After adding FBS to the cell suspension to inactivate TrypLE, the cells were passed through a 40-µm cell strainer and washed with PBS.

MACS selection was preformed using EpCaM beads and a magnetic column to further enrich for epithelia. The positive fraction was then blocked in BSA before incubating in Primary antibody, DLK1 (5µl per 1×10^6 cells per 100 ml, MM0514-9D8; Alexa Fluor® 488, Novus Biologicals, UK), basal marker CD49f-PECY7, (3µl per 1×10^6 cells per 100 ml; 313616 BioLegend, UK) and luminal marker CD26-APC (5µl per 1×10^6 cells per 100 ml; 563670 BD Bioscience, UK). Samples were washed and re-suspended in 1 ml of PrEGM medium (CC-3166, Lonza, USA) before being sorted using the BD FACS ARIA II cell sorter and DIVA software for analysis (BD Biosciences, UK). 4',6-diamidino-2-phenylindole (DAPI) was used for life/ dead cell discrimination at final concentration of 500 ng/ml.

Directly extracted fresh prostate epithelium from whole human prostates was used to sort and culture cells of interest and also to validate the purity of these selections. Freshly extracted whole prostate basal and luminal epithelial cells have low rates of dead cells determined by DAPI uptake live/dead discrimination, making the dissociation process unlikely to account for the marked differential viability demonstrated in vitro. The selection of smaller number of luminal cells instead reflects a very stringent protocol where either luminal or basal marker exclusivity is required, and therefore many intermediate cells (expressing both markers) are excluded. Our selection fractions described above are comparable with the fractions described by a number of publications, including those that lead to sustained luminal cell growth (Garraway *et al.*, 2010; Karthaus *et al.*, 2014; Drost *et al.*, 2016).

3-D spheroid culture

10,000 sorted cells were resuspended in 80µl of a 50:50 mixture of Matrigel (356231; BD Biosciences) and PrEGM and plated in the middle of one well of a 24-well tissue culture plate. After incubation at 37°C for 20 minutes to solidify the Matrigel, 500µl of pre-warmed PrEGM plus 10nM Dihydrotestosterone (DHT) and 10 µM Y-27632 dihydrochloride (04-0012; Stemgent, USA) was added to each well, and the plates were

transferred to a 5% CO_2 tissue culture incubator. The cells were then cultured at 37°C for 2-3 weeks with the medium replaced every 2 days until spheres reached ~100 µm in diameter. For subculture, the Matrigel was digested by incubation in 1 ml of 1 mg/ml dispase solution (07923, STEMCELL Technologies) at 37°C for 30 minutes. Spheroids were collected into 15-ml tube and dissociated enzymatically using 0.5 ml of TrypLE for 5 min at 37 °C followed by inactivation of TrypLE by FBS. The cells were passed through a 40-µm cell strainer and resuspended in 80µl of a 50:50 mixture of Matrigel (BD) and PrEGM and plated in the middle of one well of a 24-well tissue culture plate at a 1:2 ratio. Spheroids grown from FACS sorted cells were snap frozen in isopentane cooled with liquid nitrogen and serial sectioned for immunofluorescence studies described above.

Laser capture microdissection and RNA-sequencing

Laser capture microdissection (LCM) of approximately 10-20 cells was undertaken of discrete regions marking the start of the proximal CCO-deficient clone in 3 reconstructed examples (n=3 prostates). Complementary LCM was performed of the distal regions of these marked clones. Captured cells were pooled as part of a hypothesis-generating approach to identify putative stem cell markers by differential gene expression. Formal RNA sequencing was preformed by AROS Applied Biotechnology (Denmark). A Clontech SMARTer (Switching Mechanism at 5' End of RNA template, oligo dT primed) and PCR amplification of cDNA protocol was employed. Sequencing was undertaken using the Illumina HiSeq2500 platform. In total, 65 million reads were mapped (Illlumina CASAVA software) and a strategy to identify on/off patterning of gene expressions associated with the stem cell enriched LCM was employed. An arbitrary threshold set at 45 fold gave a long list of 578 gene expressions associated with the stem cell enriched area. Thereafter a formal systematic review was undertaken of all these putative stem cell genes and a shortlist of 46 candidate markers is presented (Figure 5) based on previous publications of stem cell markers. DLK1 was a previously documented candidate stem cell marker and its spatial expression at the start of clonal patches was validated with *in situ* immunofluorescence.

RNA Isolation, cDNA Preparation, RT-PCR

RNA was isolated from freshly sorted cells and spheroids using QIAGEN RNeasy kits according to manufacturers protocol. 100-1,000 ng RNA was reverse transcribed to cDNA using SuperScript™ III Reverse Transcriptase (invirtogen) according to manufacturers protocol. RT-PCR was performed according to the Taqman method (Applied Biosystems). All probes were synthesized by Applied Biosystems.

Details on the theoretical approaches used

In this Supplementary Section, we provide further details on our modelling approach to the homeostatic renewal of human prostate, as described via the lineage tracing of CCO-deficient clones. We also provide additional details on the data analysis and statistics used.

1 Assessment of clonality

1.1 Theoretical considerations

We first address the possibility that clonal streams result from multiple CCO-deficient mutations in different cells of the same prostatic subtree. Importantly, as noted in the main text (and sketched in Fig. 1A), a prostatic main duct branches out in 26 ± 2 distinct branching structures, which we will denote as subtree here and in the rest of the Supplementary Section. If we assume that CCO-deficient mutations in a given subtree appear (i.e. rise above the threshold of detection) at a constant rate λ throughout the life of an individual, the probability of n independent mutations in a given subtree after a time t follows a Poisson distribution

$$P_n(t) = \frac{(\lambda t)^n e^{-\lambda t}}{n!} \tag{1}$$

Over time, we thus expect the fraction of subtrees not marked by CCO-deficiency to decreases exponentially in time. Before using this dependence to assess clonality of the data, we first define the general statistical procedure to judge the quality of fits to the data for this measure and others.

1.2 Statistical approach to data modeling

To challenge quantitatively the goodness of fits throughout this Note, as well as their explanatory power, we each time verified the model predictions by calculating the coefficient of determination R^2, which is the simplest indicator of the goodness of a fit. In addition, we calculate the standard error of the fit S, which is an absolute measure of the residuals of the fit, and has been shown to be more adapted for non-linear fitting procedures (Brown *et al.*, 2011). For a good fit, R^2 should be as close to 1 as possible, whereas S should be as close to 0 as possible.

Specifically, defining n as the number of the points being fitted, $\bar{y} = \frac{1}{n}\sum_i^n y_{\text{obs}}^i$ the average of the observable, $S_{\text{tot}} = \sum_i^n (y_{\text{obs}}^i - \bar{y})^2$, and $S_{\text{res}} = \sum_i^n (y_{\text{obs}}^i - y_{\text{model}}^i)^2$, the coefficient of determination is defined as $R^2 = 1 - \frac{S_{\text{res}}}{S_{\text{tot}}}$, while the standard error of the fit is given by $S = \sqrt{\frac{S_{\text{res}}}{n}}$.

1.3 Model validation

Therefore, to assess clone accumulation rate, λ, we inferred the probability of that a given tree is unlabelled at time t, $P_0(t) = e^{-\lambda t}$, by measuring the fraction of prostatic subtree that do not display a CCO-deficient clonal stream, as a function of the age of the patient. A first consistency check is that P_0 is indeed a decreasing function of patient age, as expected. Simply fitting an exponential with $t = 0$ representing the birth date of subjects resulted in a poor fit ($1/\lambda = 410y \pm 20$, coefficient of determination $R^2 = 0.07$, standard error of the fit $S = 9.3\%$). However, incorporating an additional parameter t_0, representing the age at which CCO-deficient mutations start to appear and propagate allowed for a much better fit of the data ($1/\lambda = 260y \pm 45$, $t_0 = 25y \pm 7$, $R^2 = 0.72$, $S = 1.7\%$). This fit validates the idea of random mutations occurring at a very low rate in the stem cell compartment, throughout the adult life of individuals, and that this rate is near-constant both in time and across patients.

Timing of mutation acquisition Importantly, this finding argues that the streams that are observed cannot be explained statistically as a developmental or pubertal artifact. If this was so, one would not expect patients of increasing age to display more of these events. On the contrary, the roughly constant rate of mutation inferred from above indicates that the overwhelming majority of the clonal labelling events must occur during the homeostatic renewal of the prostate. Note that this is largely independent of the exact timing at which mutation start to accumulate (i.e. t_0): a possibility for the existence of this threshold could be the time/number of mutation it takes for CCO-deficiency to become visible.

Clonality of the data This then allows for a quantification of the probability of double hits $P_2(t)$ in a given subtree. We find that at the maximal age recorded (75 years), this probability is only of 1.5%, whereas in the youngest subjects this probability is as low as 0.4%. In other words, the probability that a marked subtree received 2 mutations in different stem cells is always below 10% for the oldest patients. This means *a fortiori* that the possibility of triple or more hits is completely negligible.

Moreover, one should note that the calculation presented above is a very conservative one. Indeed, it assumes that one does not have any spatial information on the structure of the clonal streams. In fact, as discussed in the main text, as the clonal streams are very coherent, and only occupy a small fraction of the most-proximal duct (on average 2.4%), one would notice double hits by seeing two streams running parallel to each other, something that we never observed experimentally. The only possibility of missing a double hit would be for the two streams to have fused, because the two mutations occurred in an adjacent region of the stem cell niche. However, this only has a probability proportional to the ductal fraction occupied by streams, and is therefore exceedingly rare. One should note that the qualitative result of this analysis does not depend on using a value of t_0 different from 0.

Altogether, the analysis and experimental data support the notion that clonal streams result from a single CCO-deficiency mutation. This was probed further experimentally by performing mtDNA sequencing (Fig. 2E) of different loci of streams (proximally and distally), and showing that they were indeed clonally related.

2 Clonal dynamics in the stem cell niche

2.1 Theoretical modelling of stem cell turnover

We first assume that the stem cell niche contains an equipotent pool of N stem cells, dividing stochastically with a division fate λ_S, producing differentiated cells of both basal and luminal lineages, which we collectively denote as D, undergoing constant and stochastic cell death and shedding with rate Γ. The dynamics of such a population follows a classical birth-death process (Athreya *et al.*, 2012, Bailey, 1964), and was shown to describe well a large number of epithelial tissues, both in mouse models at homeostasis, such as prostate (Ousset *et al.*, 2012), skin (Mascre *et al.*, 2012) or gut (Snippert *et al.*, 2010), or during tumorigenesis (Driessens *et al.*, 2012), and in human epithelia at homeostasis (Simons, 2016) and during tumorigenesis (Williams *et al.*, 2016):

$$S \xrightarrow{\lambda_S} \begin{cases} S+S & \text{Pr. } r+\Delta \\ S+D & \text{Pr. } 1-2r \\ D+D & \text{Pr. } r-\Delta \end{cases} \qquad D \xrightarrow{\Gamma} \text{loss}$$

Here the parameters r and Δ quantify, respectively, the frequency of symmetric division/differentiation events, and the imbalance towards symmetric divisions. $\Delta = 0$ corresponds to homeostasis, while $\Delta > 0$ corresponds to net growth of the stem cell population. As CCO-deficiency could *a priori* be a non-neutral marker of an expanding population, we include for now the possibility of strictly positive Δ in the model, a hypothesis which will be cross-checked *a posteriori* in the data. One should note that at homeostasis ($\Delta = 0$), the limit $r = 0$ corresponds to the classical paradigm of a purely asymmetrically dividing, immortal stem cell whereas the limit $r = 1/2$ corresponds to the limit of a purely symmetrically dividing and differentiating stem cell.

Formally, defining $P_{n_S,n_D}(t)$ as the probability to find a clone at time t post-induction containing n_S stem cells and n_D differentiated cells is described by the Master equation:

$$\begin{aligned}\frac{dP_{n_S,n_D}}{dt} = \lambda_S \big((r+\Delta)(n_S-1)P_{n_S-1,n_D} + (r-\Delta)(n_S+1)P_{n_S+1,n_D-2} \\ + (1-2r)n_S P_{n_S,n_D-1} - n_S P_{n_S,n_D-1} + \Gamma((n_D+1)P_{n_S,n_D+1} - n_D P_{n_S,n_D}) \quad (2)\end{aligned}$$

Although the solution for this Master equation can not be obtained in closed form (Antal *et al.*, 2011), the long-term distribution of the differentiated cells is proportional to the distribution of the stem cells, so that one can consider the stem cell distribution alone.

2.2 Case of a single induction at a controlled initial time

Dynamics at homeostasis: In the case of a single induction event at an initial time $P_{n_P,n_D}(0) = \delta_{n_P,1}\delta_{n_D,0}$, the distribution is predicted at long times to converge to a scaling behavior where the chance of finding a clone with n cells at time t post-labelling takes the form $P_n(t) = \frac{1}{\langle n(t)\rangle} f(\frac{n}{\langle n(t)\rangle})$, where $\langle n(t)\rangle$ denotes the average clone size, and the function $f(x) = e^{-x}$.

Two quantities of outstanding interest are the mean surviving clone size and the clonal persistence. For $\Delta = 0$, the process is critical, and previous studies have shown that the average number of basal progenitors in surviving clones is given by (Bailey, 1964, Antal *et al.*, 2011, Klein *et al.*, 2007)

$$\langle n_P(t)\rangle = 1 + r\lambda_S t \quad (3)$$

This linear increase in average size compensates for the continuous chance loss of clones through differentiation. The increase in average basal clone size is simply the inverse of the persisting clone fraction, so that their product, i.e. the labelled cell fraction, is constant in time:

$$1 - P_0(t) = \sum_{i=1}^{\infty} P_i(t) = \frac{1}{1 + r\lambda_S t} \tag{4}$$

At steady-state, the ratio of total suprabasal to basal cell number is dictated by the ratio of the division and shedding rates: $\frac{n_P^\infty}{n_D^\infty} = \frac{\Gamma}{\lambda_S}$.

Dynamics for an out-of-homeostasis system: For super-critical Watson-Galton process ($\Delta > 0$), again for a single induction event at an initial time $P_{n_P,n_D}(0) = \delta_{n_P,1}\delta_{n_D,0}$, the mean clone size is expected to grow exponentially at longer times, the persistence plateaus long-term to a non-zero value, equal to $1 - P_0 \to \frac{2\Delta}{1+\Delta}$, and the clonal distributions have been calculated explicitly in (Antal *et al.*, 2011, Klein *et al.*, 2007).

Continuous labelling: The paragraphs above dealt with the classical case of a single clonal induction event, at a controlled time, which is relevant for lineage tracing experiments based on pulse-labelling. However, in the case of CCO-deficiency in the prostate, mutations can arise at any timepoint during the life of the individual, so that there is fundamental indeterminacy in the initial condition. However, as discussed in the first section, we know that the data we consider is clonal, so that multiple induction events in the stem cell compartment of a given prostatic subtree are very rare.

2.3 Analysis of the data

When turning to the data, we must first note that assessing total clone size is exceedingly difficult, for at least two reasons. Firstly, the very large spatial extent of the clonal streams makes it hard to get a reliable estimate of cell number. Secondly, the prostatic subtrees are very irregular in nature, both in total size and in geometry, which introduces an additional source of variability in the system.

However, as discussed in the main text, one can obtain a proxy for stem cell numbers by considering the ductal fraction f_c occupied by the clonal stream in the main, most-proximal trunk of prostatic subtrees. This allows us to make abstraction of the subsequent branching pattern of the prostate, and infer the distribution of labelled stem cells $P_{n_P}(t)$ for patients of various age t. This bears strong similarities with the approach originally used in intestinal crypts, where the width of ductal ribbons imprinted on villi was used as a proxy of stem cell clone size (Lopez-Garcia *et al.*, 2010).

Neutrality of the labelling Accumulations of mtDNA mutations, studied in a wide range of tissues, suggest that these age-related changes are largely neutral in their functional effects on cells (Ju *et al.*, 2014). Using specific assays of CCO-deficient clonal marks, findings in human colonic crypts appear grossly neutral (Baker *et al.*, 2014).

Strickingly, we could find no correlation between the age of the patient and the ductal fraction occupied by the CCO-deficient stream. This striking feature excludes both the possibility of CCO-deficiency being a non-neutral marker, and of neutral drift due to cell competition occurring in the system. Indeed, in the former case, one would expect an exponential increase in the fraction f_c as a function of time, which should become evident on time scales of decades even for very small imbalances Δ. This confirms the findings discussed in the main text (Extended Data Fig. 1A,B) that the division and apoptosis rates of CCO-deficient cells are statistically undistinguishable from those of normal cells, throughout the prostate. In the latter case, one would expect a linear increase of f_c with patient age, which again would be noticeable on these timescales. In principle, this could occur because of very low division rates λ_S, or because of the more likely solution of nearly-perfectly asymmetrically dividing stem cells ($r \approx 0$). Further confirming this lack of neutral competition, we noted that the probability distribution on f_c is relatively narrow around its average value ($\langle f_c \rangle = 0.024 \pm 0.004$), instead of the broad exponential distribution expected for neutrally competing stem cells. Finally, a last evidence for neutrality, as discussed in the main text, is the fact that the total width of clonal ribbons remains approximately constant throughout the gland, again something that would not be expected in CCO-deficient progenitors outcompeted their CCO-proficient neighbours.

Estimate of functional stem cell number Moreover, given that CCO-deficiency is a neutral marker, one can estimate the average number of functional stem cells in a given prostatic subtree as $1/f_c \approx 43$. This number is very low given the size of the prostatic subtrees. Given that the prostatic epithelium at its most proximal (i.e. where the stem cell niche is located) is intertwined with urothelium in a "backgammon"-type of interdigitated structure, we reasoned that this number of functional stem cells could correspond to the number of independent niche-like structures formed at the boundary. Stem cell competition could then occur within these structures, resulting in rapid monoclonal conversion, without extending further because of the spatially segregated nature of these niches. Similarly, we cannot exclude a slight proliferative advantage of CCO-deficient basal progenitors as long as they continue dividing asymmetrically, as this would only reflect in higher luminal cell production, without competing with neighbouring streams. Of note, although the fraction f_c of labelled epithelia was well defined across patients, the width of the main duct could vary up to a factor 10, meaning that the width of a clonal stream scaled with the duct size (correlation coefficient $r = 0.57$). This could be linked to the structure of the stem cell niche, characterised by a series of inter-digitation between prostatic and urethral epithelia, as discussed in the main text and Fig. 5D-G.

3.1 Clonal dynamics along the tree structure

As stated in the main text, a key feature of the clonal data is that large clones consist of coherent streams spanning an entire prostatic subunit. As prostate consists of a series of branched ducts, we define three axes for cellular movement in a cylindrical geometry (see Fig. 1A for a schematics):

- a radial direction t_r perpendicular to the basal membrane, and along which basal cells differentiate into luminal cells, which are in turn constantly shed into the lumen.
- an orthoradial direction t_θ, parallel to the basement membrane and contained within a ductal section, along which cells can potentially diffuse, leading to pseudo-disconnections of clones (see Figure 2F)
- an axial direction t_z, running from the proximal end of a duct towards the distal end, along which clonal streams flow in a directional manner

As discussed in the main text, there are cellular movements in all three of these directions during normal prostate homeostasis, and we will therefore in the following explore how each movement impacts on the observed spatial clonal dynamics.

The first question one can ask is how the cellular streams are divided at each branching bifurcation. Assuming that a clonal stream consists, at a given ductal section of coordinate z_0 of n labelled cells out of a total of N cells, we can ask what is the probability of getting two equal streams of cell number n_1 and n_2 (subject to the constraint $n_1 + n_2 = n$), upon branch bifurcation into two new ducts of sections N_1 and N_2 (subject to the constraint $N_1 + N_2 = N$). This last contraint is equivalent to the Leonardo da Vinci's area-persevering rule for tree branching with the sum of the cross-sectional area of daughter branches equals that of the parent branch (Richter, 1939).

Clearly, the overall dynamics depends on the amount of cellular mixing, and how cohesive is the clonal stream. Two limits that can be explored analytically are the limit of perfect mixing (well-mixed regime, cells can rearrange freely) and the limit of zero mixing (cells follow perfectly cohesive streams that bifurcate following laminar flow). Clearly, the prostate data belong to the second class, given how cohesive streams are in vivo, so that we only explore this option. Although we see in the data that the sum of the perimeters of two offspring ducts after a bifurcation is typically 120% of the original duct perimeter, we can still use the perimeter ratio of both ducts as a proxy of how the flow was segregated, whereas the global increase to total perimeter upon branching will be simply resolved, for an incompressible epithelium, in a global decrease in the velocity of the flows. One therefore expects the dynamics of the flows to slow down geometrically as they pass an increasing number of bifurcations in the t_z axis. Therefore, as cells migrate along the t_z axis, they would spend more and more time residing in each branch.

3.1 Zero-dispersion, spatially structured regime

In the zero-dispersion regime, no clonal fragmentation occurs, so spatial correlations are key to understand the dynamics of flows upon bifurcation. We make the same assumption of n labelled stem cells in a total N, so that each stem cell occupies a position $i \in [1,N]$ on a torus. We define an indicator function $n_i(t)$, such that $n_i = 1$ if a cell is labelled, and $n_i = 0$ otherwise. When the flow reaches a bifurcation point, which we assume to seggregate the flow equally, $N/2$ contiguous cells are chosen, starting at a random position j, so

that

$$\begin{cases} n_{2k+1}(t+1) = n_{j+k}(t) \\ n_{2k+2}(t+1) = n_{j+k}(t) \end{cases} \quad \text{for } k \in [0, N/2-1]. \tag{5}$$

Although streams at the first bifurcation point typically span many cells, they are sufficiently small initially that we can assume they consist of a single effective cell, hence the initial condition $n_i(0) = \delta_{i,1}$, with $N = 43$ being the total number of effective stem cells. Moreover, from a numerical integration point of view, it is easier to consider that N is constant upon bifurcation, while clones double when all going into one duct, instead of the converse of considering clones constant and a value of N halved for each bifurcation. From a simple combinatorics analysis, one can arrive to simple expression for the transition rates $\Pi_{n\to k}$ under the condition $k < N/2$, depending on the value of k relative to n:

$$\begin{cases} \Pi_{n\to 2k>2n} = 0 \\ \Pi_{n\to 2n} = \frac{N/2-n+1}{N} \\ \Pi_{n\to 2k<2n} = \frac{2}{N} \end{cases} \tag{6}$$

The first line stems from the obvious fact that stem cell number cannot increase by more than a factor 2 per bifurcation event. The second line deals with the case when all labelled cells flow in a single duct. The third line deals with boundary events, which can only occur 2 times out of N, i.e. when the selected position of bifurcation is k positions away from the boundary of the clone. The master equation for $P_n(t)$ can then be written

$$\begin{cases} P_0(t+1) = P_0(t) + \sum_{k=1}^{N/2} \frac{N/2-k+1}{N} P_{2k}(t) \\ P_m(t+1) = \frac{N/2-m/2+1}{N} P_{m/2}(t) + \frac{2}{N} \sum_{k=m/2+1}^{N/2} P_{2k}(t) \end{cases} \tag{7}$$

so that the evolution of $P_0(t)$ is dictated by a very simple equation, as in the continuous limit:

$$\tau \frac{dP_0}{dt} = \frac{N/2+1}{N}(1 - P_0(t)) - \frac{1}{2N} \tag{8}$$

3.1.1 Case of very large number of stem cells $N \gg 1$

For $N \to \infty$, one expects an exponential clonal growth, as the only possible choices are either not selecting any labelled cells (probability 1/2) in a given duct, or selecting all of them (probability 1/2). The mean surviving clonal size and clonal persistence in the scaling regime are indeed

$$m(t) = e^{kt} \tag{9}$$

and

$$P_0(t) = 1 - e^{-kt} \tag{10}$$

where $k = ln(2)/\tau$ is the rate of bifurcation. As expected, the persistence converges at very long times to $1 - P_0(t) \to 1/N$, as the experiment marked initially 1 cell out of N. This means that CCO-deficient streams will occupy an ever-increasing fraction of labelled branches, but that the fraction of labelled branches themselves will go continuously down in time, until plateauing at the onset of full monoclonal conversion.

3.1.2 General case of finite N

For finite N, we need to also calculate the transition probabilities for $n > N/2$. The same combinatorics argument as before can be used to calculate the transition probabilities:

$$\begin{cases} \Pi_{n\to 2k<2n-N} = 0 \\ \Pi_{n\to 2k=2n-N} = \frac{n-N/2+1}{N} \\ \Pi_{n\to 2k>2n-N} = \frac{2}{N} \\ \Pi_{n\to N} = \frac{n-N/2+1}{N} \end{cases} \tag{11}$$

These equations can be readily solved numerically, yielding a prediction on how the clonal fraction of CCO-deficient cells evolve as a function of generation number along the t_z axis.

3.2 Comparison to the data

We performed a numerical integration of these equations for $N = 42$, and showed in Fig. 3G the predicted increase in surviving clone size (i.e. the fraction of a duct labelled), as a function of bifurcation number.

Overlaying this prediction with the real data from spatial reconstructions revealed a good quantitative agreement ($R^2 = 0.82$, $S = 0.09$), arguing that although ducts do not always bifurcate equally, our simple theory captures the essence of monoclonal conversion. Unfortunately, we did not have a sufficient amount of data to plot the clone size distribution at a given bifurcation level in a meaningful statistical way, to check that it conformed to the expected exponential distribution.

However, we wanted to prove in further quantitative details that clonal streams are indeed neutrally segregated at branching points (rather for instance than being biased in a given region of the subtree, or towards smaller terminal acini structure). We therefore went back to our dataset, and calculated the clone fractions and duct perimeters before and after each bifurcation (Fig. 2D) as well as scored which duct the clonal stream went in.

This allowed us to probe in more details whether the system conforms statistically to our transition probabilities, which we recalculated for ducts of different sizes. Noting f_d the share of total perimeter of the bigger duct after bifurcation and f_c the fraction occupied by the clonal stream before bifurcation, we could predict a probability of bifurcating either in the small, the big, or both ducts (respectively noted P_s, P_b and P_d such as $P_s + P_b + P_d = 1$). We can calculate that for $f_c < 1/2$: $P_d = 1 - 2f_c$ and $P_s = 1 - f_d - f_c$, while for $f_c > 1/2$: $P_s = 0$ and $P_d = f_c - f_d$. Importantly, we first performed the control that total fraction marked by a stream is conserved before and after bifurcation, providing a first confirmation of the neutrality of the process (Fig. 2C and Extended Data Fig. 2). Although the outcome of a bifurcation depends on the initial clonal fraction f_c and the relative size of the bigger duct f_d, we did not have enough data to investigate this functional relationship. We therefore calculated the predicted probabilities P_s, P_b and P_d for each individual event, using the real value of f_c and f_d, and averaged those across the 22 bifurcation points of the dataset. We then compared these computed probabilities to the actual probabilities, revealing a good quantitative agreement again (Fig. 2D). This provides an additional test of our model and of our mechanism of segregation upon bifurcation.

3.3 Lineage hierarchy between basal and luminal cells distally

Estimating the typical velocity of the flows In principle, the presence of proximal stem cells, along with possible transit amplifying progenitors in the most-proximal duct, would be enough to fuel the renewal of the entire distal tree, as is the case for the intestinal epithelium. However, two lines of arguments make this model unlikely. First, a given prostatic tree branches out multiple times, with the total perimeter of the resulting ducts being larger than the original duct (by a factor $f > 1$). Even if cellular loss was negligible, this means that the velocity of the flows would decrease geometrically by a factor f^n after n bifurcation events, resulting in extremely low rates of prostate renewal. Although the prostate epithelium is known to be slow-cycling, it should be noted that the vast majority of the clones observed spanned the entire tree, meaning that they had the time to reach the distal part of the subtree between clonal induction and patient death. As labelling occurs throughout the lifetime of the patient T, the probability of finding a clone that did not reach the distal part of the prostate scales is T_r/T, where T_r is the characteristic time for a cell to migrate from the proximal stem cell niche to the distal end of the tree. Note that this makes the simplifying assumption that clones spans similar length. In fact, a more detailed calculation of the probability is made difficult by the fact that the tree topology is stochastic and therefore that some streams will randomly bifurcate and terminate in less distal parts of the tree than others. Moreover, because of rearrangements that tend to cause pseudo-disconnection of clones, a possible origin for the clones which did not reach the distal end of the tree could be that they only recently underwent rearrangement, and not that they were recently acquired proximal mutations.

Nevertheless, given the rarity of clones not spanning the full proximal-distal axis of a tree (2 out of 20), we can still put an upper bound on the typical time of renewal, to $T_r < 10 years$. A lower bound for T_r is harder to get, as this requires, as discussed above, to make assumptions on whether distal proliferation and apoptosis are exactly balanced or not. However, given the relatively slow fraction of Ki67+ cells, even in the proximal basal layer, one can estimate a division rate of basal progenitors to around a week, consistent with BrdU pulse and contemporary heavy water (^2H_2O) tracing experiments in live patients are available in the literature (Nemoto *et al.*, 1990, Borre *et al.*, 1998, Hayes *et al.*, 2012). Therefore, even in the extreme case of having the proximal 10% of the basal cells dividing symmetrically to renew the 90% more distal, one would still require 10 rounds of division, i.e. several months, to renew the full prostate (whereas estimates based on exclusive asymmetric division would require at least a year). Therefore, we can estimate that the typical turnover time for long-range flows ranging between a few months and a few years.

Inference of the lineage hierarchy between basal and luminal progenitors Secondly, however, this conservative estimate does not take into account the large amount of cellular loss via apoptosis that we observe experimentally (Fig. 4E). If this was not compensated by distal proliferation, the cellular streams arising from the proximal niche would decay exponentially, and be unable to fuel the entire prostatic epithelium. Indeed, when staining for Ki67, we observe a persistently non-zero rate of cell division in the distal region, although not as high as in the proximal part of the tree. This is fully consistent with progenitors compensating for cellular loss, and serving as distal relays for the proximal-source of streaming.

We next wondered about the lineage hierarchy underpinning these distal progenitors. Interestingly, we noticed that distal apoptosis was almost entirely restricted to the luminal epithelium, whereas distal proliferation was largely restricted to the basal epithelium. Given that the proportions of basal and luminal cells does not change significantly along the proximo-distal axis, this means that basal progenitors must sit at the top of the distal hierarchy, and give rise to luminal cells that have very limited proliferative capacity and are periodically shed.

This conclusion can also be reached from independent considerations on the cohesiveness of the basal and luminal streams. Given the propensity of streams to slightly diffuse along the orthoradial axis, one would expect independent luminal and basal compartments to diffuse out of register, and have a finite probability to adopt different paths distally in the epithelium. However, we scored 10 distal patches (n=5 from fully reconstructed clonal trees and n=5 from disconnected clones from proximal patches that were partially reconstructed) and always found (100%) very cohesive patches, where basal and luminal cells were perfectly registered. Similarly, if basal and luminal cells were independently maintained by loss-replacement dynamics, one would expect both compartments to undergo independent neutral drifts, resulting in independent sizes (for a sketch of a simulation output with periodic boundary conditions along the orthoradial axis, see Extended Data Fig. 4C). Again, this is never observed.

4. Conclusion

Thus, taken together, these data uncover an unexpected mode of homeostatic renewal of the adult human prostate. Instead of being renewed by progenitors dispersed throughout the epithelium, each prostatic subtree is maintained by a very small number of functional basal stem cells, that are located proximally on the boundary between the prostate and the urothelial epithelium. These basal stem cells thus maintain the entire subtree by cohesive proximal to distal streams, that flow and get neutrally segregated at branch points, resulting in monoclonal conversion of entire distal parts of the subtree. These flows would rapidly exhaust if they were not relayed by a distal population, which gives rise to luminal cells that are rapidly shed away. Within the resolution of the data, it is not possible to deduce whether basal progenitors can directly produce luminal cells, or if they do so via luminally-committed basal cells that mature and migrate to the luminal cell layer. However, there must be a small population of proximal self-renewing luminal stem cells that maintain the luminal cells in the most proximal part of the subtree, where the basal streams have not yet converted luminally. An intriguing feature of these streams is their cohesiveness over very large distances, even though CCO-deficiency seems to behave as a neutral marker. One option would be that each stream has a memory of the stem cell from which is originates, and thus has a surface tension relative to other streams from other stem cells. Other options would be that epithelial cells are tightly adhered to each other, resulting in minimal dispersion, and/or undergo coupled, collective cell migration, analogous to flocking mechanisms, that have already been proposed in a variety of biological systems (Szabo *et al.*, 2006). Further investigations will be necessary to answer these questions, both on human prostate and on possible other model systems displaying similar dynamics.

References

Blackwood, J.K., Williamson, S.C., Greaves, L.C., Wilson, L., Rigas, A.C., Sandher, R., Pickard, R.S., Robson, C.N., Turnbull, D.M., Taylor, R.W. and Heer, R. (2011) 'In situ lineage tracking of human prostatic epithelial stem cell fate reveals a common clonal origin for basal and luminal cells', *J Pathol*, 225(2), pp. 181-8.

Coller, H.A., Khrapko, K., Bodyak, N.D., Nekhaeva, E., Herrero-Jimenez, P. and Thilly, W.G. (2001) 'High frequency of homoplasmic mitochondrial DNA mutations in human tumors can be explained without selection', *Nat Genet*, 28(2), pp. 147-50.

Drost, J., Karthaus, W.R., Gao, D., Driehuis, E., Sawyers, C.L., Chen, Y. and Clevers, H. (2016) 'Organoid culture systems for prostate epithelial and cancer tissue', *Nat Protoc*, 11(2), pp. 347-58.

Elson, J.L., Samuels, D.C., Turnbull, D.M. and Chinnery, P.F. (2001) 'Random intracellular drift explains the clonal expansion of mitochondrial DNA mutations with age', *Am J Hum Genet*, 68(3), pp. 802-6.

Fellous, T.G., Islam, S., Tadrous, P.J., Elia, G., Kocher, H.M., Bhattacharya, S., Mears, L., Turnbull, D.M., Taylor, R.W., Greaves, L.C., Chinnery, P.F., Taylor, G., McDonald, S.A., Wright, N.A. and Alison, M.R. (2009) 'Locating the stem cell niche and tracing hepatocyte lineages in human liver', *Hepatology*, 49(5), pp. 1655-63.

Garraway, I.P., Sun, W., Tran, C.P., Perner, S., Zhang, B., Goldstein, A.S., Hahm, S.A., Haider, M., Head, C.S., Reiter, R.E., Rubin, M.A. and Witte, O.N. (2010) 'Human prostate sphere-forming cells represent a subset of basal epithelial cells capable of glandular regeneration in vivo', *Prostate*, 70(5), pp. 491-501.

Greaves, L.C., Nooteboom, M., Elson, J.L., Tuppen, H.A., Taylor, G.A., Commane, D.M., Arasaradnam, R.P., Khrapko, K., Taylor, R.W., Kirkwood, T.B., Mathers, J.C. and Turnbull, D.M. (2014) 'Clonal expansion of early to mid-life mitochondrial DNA point mutations drives mitochondrial dysfunction during human ageing', *PLoS Genet*, 10(9), p. e1004620.

Greaves, L.C., Preston, S.L., Tadrous, P.J., Taylor, R.W., Barron, M.J., Oukrif, D., Leedham, S.J., Deheragoda, M., Sasieni, P., Novelli, M.R., Jankowski, J.A., Turnbull, D.M., Wright, N.A. and McDonald, S.A. (2006) 'Mitochondrial DNA mutations are established in human colonic stem cells, and mutated clones expand by crypt fission', *Proc Natl Acad Sci U S A*, 103(3), pp. 714-9.

Karthaus, W.R., Iaquinta, P.J., Drost, J., Gracanin, A., van Boxtel, R., Wongvipat, J., Dowling, C.M., Gao, D., Begthel, H., Sachs, N., Vries, R.G., Cuppen, E., Chen, Y., Sawyers, C.L. and Clevers, H.C. (2014) 'Identification of multipotent luminal progenitor cells in human prostate organoid cultures', *Cell*, 159(1), pp. 163-75.

McNeal, J.E. (1968) 'Regional morphology and pathology of the prostate', *Am J Clin Pathol*, 49(3), pp. 347-57.

Taylor, R.W., Barron, M.J., Borthwick, G.M., Gospel, A., Chinnery, P.F., Samuels, D.C., Taylor, G.A., Plusa, S.M., Needham, S.J., Greaves, L.C., Kirkwood, T.B. and Turnbull, D.M. (2003) 'Mitochondrial DNA mutations in human colonic crypt stem cells', *J Clin Invest*, 112(9), pp. 1351-60.
